# Antiviral and anti-inflammatory activities of chemical constituents from twigs of *Mosla chinensis* Maxim

**DOI:** 10.1007/s13659-024-00448-w

**Published:** 2024-05-01

**Authors:** Shi-Yan Feng, Na Jiang, Jia-Ying Yang, Lin-Yao Yang, Jiang-Chao Du, Xuan-Qin Chen, Dan Liu, Rong-Tao Li, Jin-Dong Zhong

**Affiliations:** Faculty of Life Science and Technology, Kunming University of Science and Technology, Kunming, 650500 Yunnan People’s Republic of China

**Keywords:** *Mosla chinensis* Maxim, Flavonoids, Phenolic structure, Anti-H1N1 virus activity, Anti-inflammatory activity

## Abstract

Seven undescribed compounds, including three flavones (**1–3**), one phenylpropanoid (**19**), three monoaromatic hydrocarbons (**27–29**), were isolated from the twigs of *Mosla chinensis* Maxim together with twenty-eight known compounds. The structures were characterized by HRESIMS, 1D and 2D NMR, and ECD spectroscopic techniques. Compound **20** displayed the most significant activity against A/WSN/33/2009 (H1N1) virus (IC_50_ = 20.47 *μ*M) compared to the positive control oseltamivir (IC_50_ = 6.85 *µ*M). Further research on the anti-influenza mechanism showed that compound **20** could bind to H1N1 virus surface antigen HA1 and inhibit the early attachment stage of the virus. Furthermore, compounds **9**, **22**, **23**, and **25** displayed moderate inhibitory effects on the NO expression in LPS inducing Raw 264.7 cells with IC_50_ values of 22.78, 20.47, 27.66, and 30.14 *µ*M, respectively.

**Graphical Abstract:**

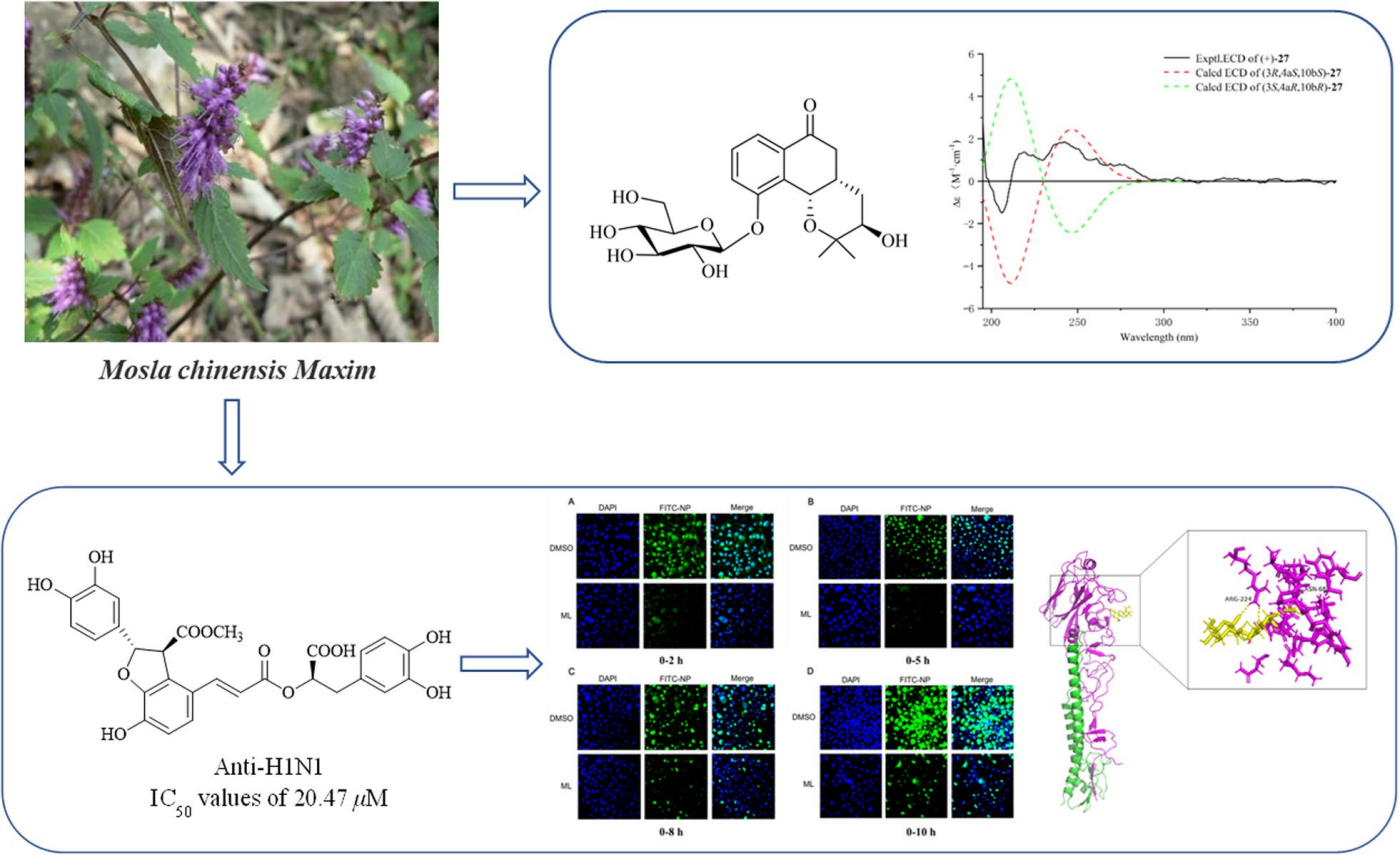

**Supplementary Information:**

The online version contains supplementary material available at 10.1007/s13659-024-00448-w.

## Introduction

Influenza viruses had high pathogenicity and infectiousness, and is an important risk factor for human health. It had been exhibited the ability to invade the epithelial cells of the respiratory tract for the happening of the inflammation, and thereby result in influenza with the symptom such as fever, headache, and muscle pain. Influenza is one of the most common respiratory diseases. If the patients had not effective medical interventions, it could induce serious complications such as pneumonia, acute lung injury and even pulmonary fibrosis [1, 2]. Influenza viruses induced diseases had been become a worldwide public health problem and the main treatment is vaccine or drug. However, because of the extraordinary high rate of virus mutation and the side effects of existing drugs, it’s essential to find ingredients with high effect and low toxicity from natural food. Phenolic compounds containing multiple phenolic hydroxyl groups, which can bind with targeting proteins of disease and possess significant activities of antioxidant, antiviral, and anti-inflammatory.

*Mosla chinensis* Maxim, recorded in Chinese Pharmacopoeia, is a medicinal and edible plant, which mainly distributed in southern China [3]. It belongs to the Labiatae family, a tomentose and aromatic plant that traditionally has been used as an herbal drug to treat colds in wet summers and aversion to cold with fever [4]. The leaves of *M*. *chinensis* are widely used as vegetable, herbal tea, beverage or food additives because of its human beneficial properties in China. Furthermore, *M*. *chinensis* is a productive source of essential oil and flavonoids. Several investigations have shown that essential oil of *M*. *chinensis* have the activities of antioxidant and antimicrobial [5–9] and the flavonoids exhibited the activities of anti-influenza A virus [10]. In our continuous search for compound of anti-influenza virus [11, 12], we found that few studies on the antiviral activity of other compounds isolated from *M. chinensis* were carried out. So, this paper was focus on exploring the activity of phenolic compounds. In this study, we investigated the extraction, structural analysis, biological activities and their possible mechanism researches of 35 compounds (covering 7 undescribed compounds and 28 known compounds) in the *M. chinensis* twigs.

## Results and discussion

### Structure characterization of the isolated compounds from *M. chinensis*

A comprehensive phytochemical investigation resulted in the isolation and identification of 35 compounds including seven new compounds (**1–3, 19,** and **27–29)** and twenty-eight known compounds. The known compounds were listed as follows: 8-(4ʹʹ-hydroxyphenyl)-5,7,4ʹ-trihydroxyflavone (**4**) [13], apigenin-7-*O*-glucuronide methyl ester (**5**) [14], acacetin-7-*O*-glucuronide methyl ester (**6**) [15], Isolinariin B (**7**) [16], acacetin 7-*O*-[6ʹʹʹ-*O*-acetyl-*β*-*D*-galactopyranosyl-(1 → 3)]-*β*-*d*-xylopyranoside (**8**) [17], luteolin (**9**) [18], apigenin 7-*O*-*β*-*D*-glucopyranoside (**10**) [19], apigenin 4ʹ-*O*-*β*-*D*-glucopyranoside (**11**) [19], acacetin 7-*O*-*β*-*D*-xylopyranoside (**12**) [20], 4ʹ,5,7-trihydroxy-3ʹ,5ʹ-dimethoxyflavone 7-*O*-[*β*-*D*-apiofuranosyl (1ʹʹʹ → 2ʹʹ)]-*β*-*D*-glucopyranoside (**13**) [21], acacetin 7-*O*-*β*-*D*-apiofuranosyl-(1ʹʹʹ → 2ʹʹ)-*β*-*D*-glucopyranoside (**14**) [22], Diosmetin 7-*O*-*β*-*D*-xylopyranoside (**15**) [23], acacetin 7-*O*-[4ʹʹʹ-*O*-acetyl-*β*-*D*-apiofuransyl-(1ʹʹʹ → 3ʹʹ)]-*β*-*D*-xylopyranoside (**16**) [24], Sakuranetin (**17**) [25], Pyrroside A (**18**) [26], methyl lithospermate (**20**) [27], dimethy lithospermate (**21**) [27], hyprhombins A (**22**) [28], 3-(3ʹʹʹ,4ʹʹʹ-dihydroxyphenyl)-acrylic acid 1-(3ʹʹ,4ʹʹ-dihydroxyphenyl)-2-methoxycarbonylethyl ester (**23**) [29], sebestenoids C (**24**) [30], methyl salvianol acid C (**25**) [31], agrimonolide 6*-O-β-D-*glucopyranoside (**26**) [32], 3ʹ-hydroxyphenyl-3,4,5-trimethylgallate (**30**) [33], 4-[[(4-hydroxybenzoyl)oxy]methyl]phenyl-*β-D*-glucopyranoside (**31**) [34], 4-*O-β-D*-glucopyranosylbenzyl-3ʹ-hydroxyl-4ʹ-methoxybenzoate (**32**) [34], 4-[[(2ʹ,5ʹ-dihydroxybenzoyl)oxy]methyl]phenyl*-O-β-D-*glucopyranoside (**33**) [19], amburoside A (**34**) [35], 4-hydroxybenzyl alcohol 4-*O*-[5-*O*-(4-hydroxy)benzoyl]- *β-D-*apiofuranosyl (1 → 2)*-β-D*-glucopyranoside (**35**) [36].

Compound **1**, yellow amorphous powder, gave the molecular formula of C_26_H_28_O_13_ based on its HR-ESI–MS ([M + Na] ^+^ *m/z* 571.1408, calcd. 571.1428). The ^1^H NMR spectroscopic data (Table 1) of **1** showed the signals for six aromatic protons at *δ*_H_ 8.09 (2H, d, *J* = 8.6 Hz, H-2ʹ/6ʹ), 7.14 (2H, d, *J* = 8.6 Hz, H-3ʹ/5ʹ), 6.83 (1H, s, H-8), 6.41 (1H, s, H-6), an olefinic proton at *δ*_H_ 6.97 (1H, s, H-3), a hydroxyl at *δ*_H_ 12.96 (1H, s), and one methyl group at *δ*_H_ 3.87 (3H, s, H-OMe). Two anomeric protons *δ*_H_ 5.35 (1H, d, *J* = 6.7 Hz, H-1ʹʹʹ), 5.18 (1H, d, *J* = 7.5 Hz, H-1ʹʹ) were observed, which imply the presence of two aglycons. Acid hydrolysis afforded two sugar components as detected by the coupling constant values (*J*_H-1ʹʹ, H-2ʹʹ_) and the GC analysis as *β-D-*xylose and *β-D-*apiose (Additional file 1: Fig. S60). The ^13^C NMR spectrum of **1** (Table 1) showed characteristic signals for the flavonoid skeleton at *δ*_C_ 182.5 (C-4), 164.2 (C-2), 99.8 (C-6), and 95.0 (C-8). The pyranose from of the sugars was also revealed from the ^13^C-NMR chemical shift values (Table 1) [37–39]. Based on ^1^H, ^13^C NMR and HSQC data, the signals at *δ*_C_ 163.2 (C-4ʹ), 162.9 (C-7), 161.3 (C-5), *δ*_H_ 6.83 (1H, s, H-8), and 6.41 (1H, s, H-6) shows that **1** is an 5,7,4ʹ-trisubstituted flavonoid.

**Table 1:** ^1^H (600 MHz) and ^13^C (150 MHz) NMR data of compounds **1–3** (*δ* in ppm, *J* in Hz)

Position	**1** (DMSO-*d*_6_)			**2** (DMSO-*d*_6_)			**3** (DMSO-*d*_6_)	
	*δ*_C_	*δ*_H_		*δ*_C_	*δ*_H_		*δ*_C_	*δ*_H_
2	164.2			164.3			164.1	
3	104.2	6.97 (s)		104.3	6.99 (s)		104.0	6.73 (s)
4	182.5			182.5			182.3	
5	161.3			161.6			161.6	
6	99.8	6.41 (s) 1.29 (m)		97.9	6.40 (s) 1.47 (m)		99.3	6.41 (s)
7	162.9			163.0			162.6	
8	95.0	6.83 (s)		95.0	6.79 (s)		94.8	6.83 (s)
9	157.5			157.4			157.3	
10	105.9			105.9			105.7	
5-OH		12.96 (s)			12.97 (s)			12.79 (s)
1ʹ	123.1			123.1			123.1	
2ʹ	129.0	8.09 (d, 8.6)		129.0	8.08 (d, 8.5)		114.5	6.81 (s)
3ʹ	115.1	7.14 (d, 8.6)		115.1	7.14 (d, 8.5)		147.6	
4ʹ	163.2			162.8			150.6	
5ʹ	115.1	7.14 (d, 8.6)		115.1	7.14 (d, 8.5)		119.9	7.10 (d, 8.4)
6ʹ	129.0	8.09 (d, 8.6)		129.0	8.08 (d, 8.5)		128.8	7.98 (d, 8.4)
Xyl/Glc								
1ʹʹ	98.9	5.18 (d, 7.5)		101.6	5.25 (d, 7.8)		98.5	5.18 (d, 7.5)
2ʹʹ	76.5	3.52 (t, 8.1)		76.9	4.04 (m)		77.1	3.52 (t, 8.1)
3ʹʹ	79.7	3.66 (d, 9.4)		79.8	4.35 (m)		78.0	3.66 (d, 9.4)
4ʹʹ	69.9	3.43 (m)		70.4	4.01 (m)		69.9	3.43 (m)
5ʹʹ	64.6	3.77 (m)		75.5	3.96 (m)		67.9	3.77 (m)
		3.29 (m)						3.29 (m)
6ʹʹ				63.7	3.79 (m)			
					3.20 (m)			
7ʹʹ				170.5				
8ʹʹ				20.7	1.85 (s)			
Api								
1ʹʹ	109.3	5.35 (d, 6.7)		108.5	5.29 (d, 6.8)		108.7	5.35 (d, 6.7)
2ʹʹʹ	77.1	3.88 (m)		77.8	3.75 (m)		78.1	3.88 (m)
3ʹʹʹ	76.0			76.9			75.7	
4ʹʹʹ	74.4	3.79 (m)		74.4	3.56 (m)		74.2	3.79 (m)
		3.42 (m)			3.50 (m)			3.42 (m)
5ʹʹʹ	66.1	3.86 (m)		67.6	3.66 (m)		66.2	3.86 (m)
		3.46 (m)			3.52 (m)			3.46 (m)
6ʹʹʹ				170.7				
7ʹʹʹ				21.1	2.01 (s)			
OMe	56.1	3.87 (s)		56.3	3.87 (s)		56.1	3.87 (s)
							56.1	3.67 (s)

The ^1^H and ^13^C NMR spectroscopic data of **1** (Table 1) is highly similar to acacetin [40], except for the additional presence of sugar moiety in **1**. The above observation indicated **1** was glycoside derivative of acacetin. The position of glycosyl junction was identified by HMBC map. A series of HMBC correlations from H_Xyl_-1 (*δ*_H_ 5.18) to C-7 (*δ*_C_ 162.9), from H_Api_-1 (*δ*_H_ 5.35) to C_Xyl_-4 (*δ*_C_ 69.9), from H_Xyl_-4 (*δ*_H_ 3.43) to C_Api_-1 (*δ*_C_ 109.3), enable the sugar chain of C-7 to be assigned as 7-*O*-[*β-D-*apiofuransyl-(1ʹʹʹ → 4ʹʹ)]-*β-D*-xylopyranoside. Thus, the structure of **1** was elucidated as acacetin 7*-O*-[*β-D*-apiofuransyl-(1ʹʹʹ → 4ʹʹ)]-*β-D*-xylopyranoside.

**Fig. 1:**
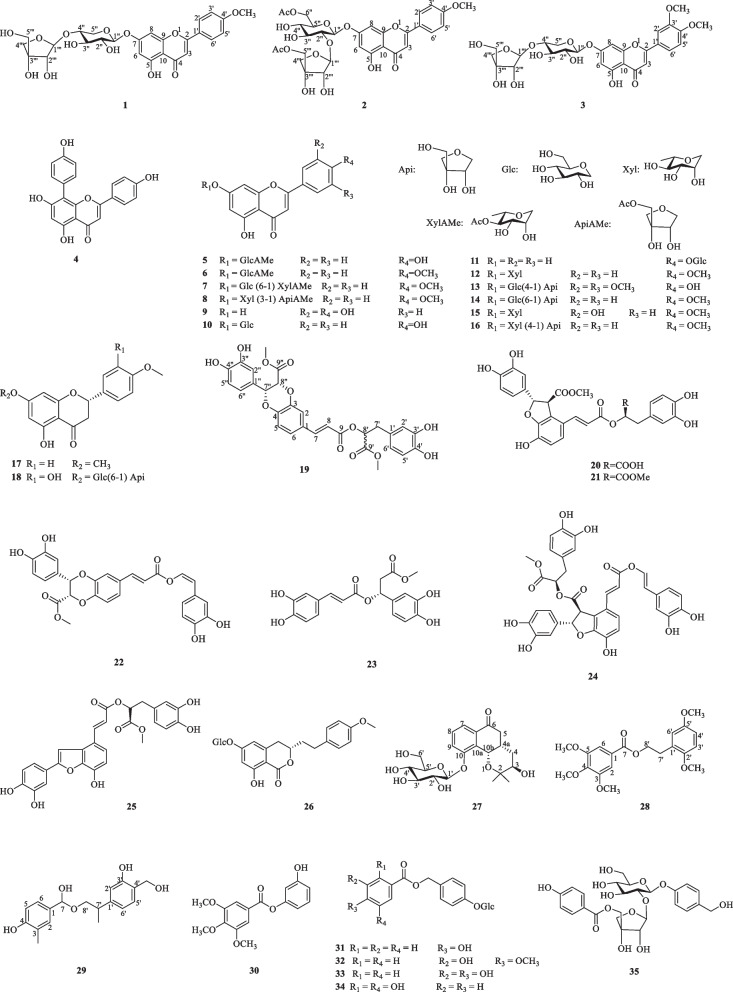
Structures of compounds **1–35** isolated from *M. chinensis* Maxim

Compound **2** was purified as a yellow amorphous powder with the molecular formula of C_31_H_34_O_16_ according to the HR-ESI–MS spectrum ([M + H] ^+^ *m/z* 663.1910, calcd. 663.1920). The ^1^H and ^13^C NMR spectroscopic data of **2** (Table 1) were highly analogue to those of **1** except the presence of two acetyl groups at *δ*_C_ 170.5, 170.7, 20.7, and 21.1 in **2**, as well as the minor change of chemical shifts in two sugars. Hence, **2** was deduced to be the acylated derivative of **1**. Acid hydrolysis demonstrated the glycosidic nature of **2,** which was identified as the *β-D-*glucose and *β-D-*apiose by the GC analysis and coupling constant values (*J*_H-1ʹʹ, H-2ʹʹ_) (Additional file 1: Fig. S61). The position of glycosyl junction was identified by the HMBC correlations from H_Glc_-1 (*δ*_H_ 5.25) to C-7 (*δ*_C_ 163.0), from H_Api_-1 (*δ*_H_ 5.29) to C_Glc_-2 (*δ*_C_ 76.9), from H_Glc_-2 (*δ*_H_ 4.04) to C_Api_-1 (*δ*_C_ 108.5). Furthermore, the sequence of the acetyl groups was deduced to be connected to C_Glc_-6 and C_Api_-5 due to the HMBC correlations from H_Glc_-6 (*δ*_H_ 3.79/3.20) to C-7ʹʹ (*δ*_C_ 170.5), from H_Api_-5 (*δ*_H_ 3.66/3.52) to C-6ʹʹʹ (*δ*_C_ 170.7). Therefore, compound **2** was identified as acacetin 7*-O*-[5ʹʹʹ-*O*-acetyl-*β-D*-apiofuransyl-(1ʹʹʹ → 2ʹʹ)]-6ʹʹ-*O*-acetyl- *β-D*-glucoside (Fig. 1).

**Fig. 2.**
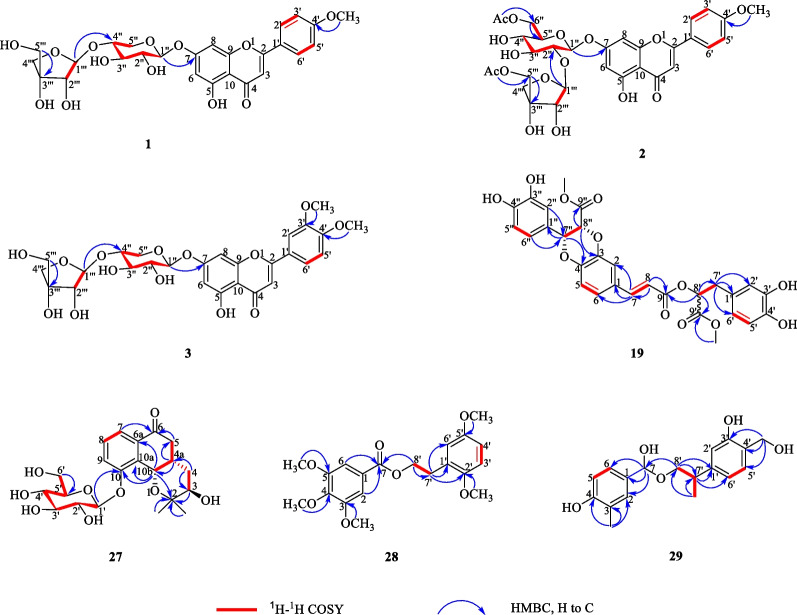
^1^H-^1^H COSY and key HMBC correlations of compounds **1–3**, **19** and **27–29**

Compound **3** was obtained as a yellow amorphous powder. It showed a quasi-molecular ion peak at *m/z* 579.1717 [M + H] ^+^ (calcd. 579.1714) in the HR-ESI–MS data, suggesting a molecular formula C_27_H_30_O_14_. The ^1^H and ^13^C NMR spectroscopic data of **3** (Table 1) were also very similar to those of** 1**, apart from the extra methoxy group at C-3ʹ in **3** rather than hydrogen group at C-3ʹ in **1**, which was supported by HMBC correlation between H-3ʹ-OMe (*δ*_H_ 3.67, s) and C-3ʹ (147.6) (Fig. 2). The anomeric configuration of *D*-xylose and *D*-apiose was confirmed to be *β-*configuration, according to the *J* value (*J* = 7.5 and 6.7 Hz) of the anomeric proton in the two sugar units (Additional file 1: Fig. S62). Thus, the structure of **3** was elucidated to be 3ʹ,4ʹ-dimethoxyluteolin-7-*O*-[*β*-*D*-apiofuransyl-(1ʹʹʹ → 4ʹʹ)]-*β*-*D*-xylopyranoside.

**Fig. 3:**
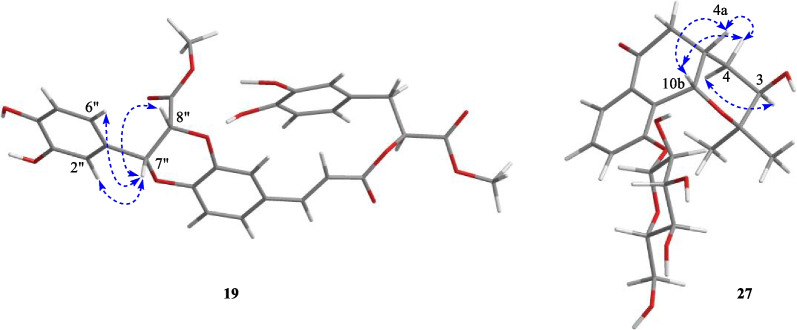
Key ROESY correlations of compounds **19** and **27**

**Fig. 4:**
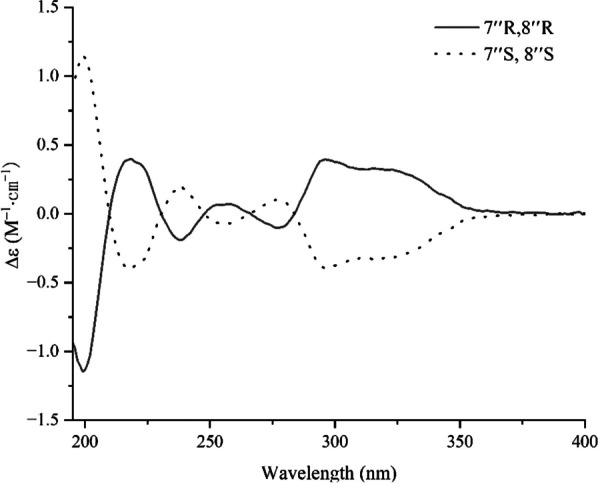
Experimental CD spectra of **19**

Compound **19**, white amorphous powder, gave molecular formula of C_29_H_26_O_12_ deduce from the HRESIMS spectrum (*m/z* 589.1293 [M + Na] ^+^, calcd. 589.1322). The ^1^H NMR spectroscopic data (Table 2) of **19** showed signals for two olefinic methine protons at *δ*_H_ 7.58 (1H, d, *J* = 16.0 Hz, H-7), 6.37 (1H, d, *J* = 16.0 Hz, H-8), three oxymethine protons at *δ*_H_ 5.19 (1H, dd, *J* = 8.0, 5.0 Hz, H-8ʹ), 5.14 (1H, d, *J* = 5.0 Hz, H-7ʹʹ), and 5.07 (1H, d, *J* = 5.0 Hz, H-8ʹʹ), two methoxy protons at *δ*_H_ 3.69 (3H, s, H-9ʹʹ-OMe) and 3.62 (3H, s, H-9ʹ-OMe), two methylene protons at *δ*_H_ 3.03 (1H, dd, *J* = 13.5, 8.0 Hz)/3.31 (1H, dd, *J* = 13.5, 5.8 Hz), and nine phenyl protons. The spectra of ^13^C NMR and DEPT displayed twenty-nine carbon signals, covering twelve quaternary carbons [containing three carbonyls at *δ*_C_ 170.7 (C-9ʹʹ), 168.1 (C-9ʹ), 166.5 (C-9)], fourteen methines (including three oxymethines at *δ*_C_ 76.6, 75.8, and 73.4, two olefinic carbons at *δ*_C_ 145.9 and 116.1, and nine phenyl carbons), one methylene, and two methyls. A comprehensive analysis of the ^1^H and ^13^C NMR spectral data (Table 2) revealed that **19** was a lignin, structurally comparable to clinopodic acid C [41] except for the lack of signals for two methoxy groups at C-9ʹ and C-9ʹʹ in clinopodic acid C (Fig. 2). The two methoxy groups were assumed to be connected to C-9ʹ and C-9ʹʹ, respectively, according to the HMBC correlations of H-9ʹʹ-OMe (*δ*_H_ 3.69) with C-9ʹʹ (*δ*_C_ 170.7) and H-9ʹ-OMe (*δ*_H_ 3.62) with C-9ʹ (*δ*_C_ 168.1). According to biogenetic considerations and key ROESY correlations (Fig. 3) observed between H-7ʹʹ/H-8ʹʹ, H-7ʹʹ/H-6ʹʹ and H-7ʹʹ/H-2ʹʹ suggested that H-7ʹʹ and H-8ʹʹ were located on the same side of the ring. The CD spectrum showed a negative Cotton effect at 244 nm (Fig. 4), suggesting that the absolute configuration of the benzodioxane moiety was established as 7ʹʹ*R*, 8ʹʹ*R* [41]. Therefore, compound **19** is identified as 9ʹ,9ʹʹ-dimethyl clinopodic acid C.

**Table 2:** ^1^H (600 MHz) and ^13^C (150 MHz) NMR data of compounds **19**, **27–29** (in ppm, *J* in Hz)

Position	**19** (CD_3_OD)			**27** (CD_3_OD)			**28** (CD_3_OD)			**29** (CD_3_OD)	
	*δ*_C_	*δ*_H_		*δ*_C_	*δ*_H_		*δ*_C_	*δ*_H_		*δ*_C_	*δ*_H_
1	128.4						126.8			133.5	
2	117.1	7.91 (d, 2.0)		73.5			107.8	7.25 (s)		128.5	6.33 (s)
3	143.2			76.4	3.40 (m)		154.4	6.73 (s)		122.1	
4	144.4			34.0	1.40 (ddd, 14.2, 10.8, 3.6)		143.5			154.0	
					1.32 (ddd, 14.2, 10.8, 2.1)						
4a				38.8	2.68 (m)						
5	116.7	6.96 (d, 8.0)		37.9	3.16 (dd, 16.3, 12.8)		154.4			114.5	7.09 (d, 8.1)
					2.61 (dd, 16.3, 3.8)						
6	122.4	7.14 (dd, 8.0, 2.0)		199.7			107.8	7.25 (s)		130.0	7.10 (d, 8.1)
6a				133.5							
7	145.9	7.58 (d, 16.0)		121.2	7.67 (d, 8.0)		167.5			79.4	5.50 (s)
8	116.1	6.37 (d, 16.0)		130.5	7.45 (t, 8.0)						
9	166.5			122.9	7.54 (d, 8.0)						
10				157.8							
10a				133.7							
10b				67.0	5.17 (d, 3.1)						
11				25.9	1.10 (s)						
12				24.6	1.04 (s)						
1ʹ	127.3			103.1	5.01 (d, 7.7)		132.3			137.4	
2ʹ	117.1	6.82 (d, 2.0)		75.1	3.60 (dd, 9.2, 7.7)		149.2			111.8	6.58 (s)
3ʹ	144.0			78.4	3.50 (m)		114.0	6.90 (d, 8.2)		157.1	
4ʹ	144.8			71.2	3.42 (m)		122.5	6.85 (d, 8.2)		127.4	
5ʹ	115.0	6.69 (d, 8.0)		77.9	3.50 (m)		150.4			130.0	7.10 (d, 8.1)
6ʹ	120.4	6.56(dd, 8.0, 2.0)		62.4	3.92 (dd, 12.1, 2.2)		113.0	6.88 (s)		114.5	7.09 (d, 8.1)
					3.73 (dd, 12.1, 5.6)						
7ʹ	36.5	3.03 (dd, 13.5, 8.0)					37.9	3.00 (t, 6.8)		31.6	2.75 (qt, 7.1, 3.2)
		3.31 (dd, 13.5, 5.8)									
8ʹ	73.4	5.19 (dd, 8.0, 5.0)					66.9	4.48 (t, 6.6)		69.1	3.92 (dd, 11.2, 3.7)
											3.78 (dd, 11.2, 2.9)
9ʹ	168.1										
1ʹʹ	126.5										
2ʹʹ	114.9	6.71 (d, 2.0)									
3ʹʹ	145.2										
4ʹʹ	145.4										
5ʹʹ	113.8	6.76 (d, 8.0)									
6ʹʹ	118.6	6.85 (dd, 8.0, 2.0)									
7ʹʹ	76.6	5.14 (d, 5.0)									
8ʹʹ	75.8	5.07 (d, 5.0)									
9ʹʹ	170.7										
3-Me										14.5	2.00 (s)
3-OMe							56.6	3.85 (s)			
4-OMe							56.4	3.79 (s)			
5-OMe							56.6	3.85 (s)			
2ʹ-OMe							61.1	3.81 (s)			
4ʹ-CH_2_										62.9	3.34 (s)
5ʹ-OMe							56.5	3.78 (s)			
7ʹ-Me										16.9	1.38 (d, 7.1)
9ʹ-OMe	51.6	3.62 (s)									
9ʹʹ-OMe	51.3	3.69 (s)

Compound **27** was isolated as a colorless oil. The molecular formula of **27** was assigned as C_21_H_28_O_9_ based on the HR-ESI–MS at *m/z* 447.1638 [M + Na] ^+^ (calcd. 447.1631). Analyses of the 1D NMR data (Table 2) of **27** revealed signals for one ketone (*δ*_C_ 199.7), one trisubstituted benzene ring [*δ*_C_ 157.8, 133.7 and 133.5; *δ*_H_ 7.67 (d), 7.54 (d) and 7.45 (t)], and two methyls [*δ*_C_ 25.9 and 24.6; *δ*_H_ 1.10 (s) and 1.04 (s)]. In addition, one anomeric proton at *δ*_H_ 5.01 (1H, d, *J* = 7.7 Hz, H-1ʹ) was observed, which implies the presence of one aglycon. Acid hydrolysis of **27** afforded one sugar moiety, which was identified as the *β-D-*glucose by the GC analysis and coupling constant values (*J*_H-1ʹ, H-2ʹ_) (Additional file 1: Fig. S63). The 1D NMR spectroscopic data of **27** (Table 2) were virtually identical to those of (3*R*,4a*R*,10b*R*)-3,10-dihydroxy-2,2-dimethyl-3,4,4a,10b-tetrahydro-2H-naphtho[1,2-b]-pyran-5H-6-one [42]. The major difference is the presence glycosyl group of located at C-10 in **27** as reinforced by the HMBC correlations from H_Glc_-1 (*δ*_H_ 5.01) to C-10 (*δ*_C_ 157.8).

**Fig. 5:**
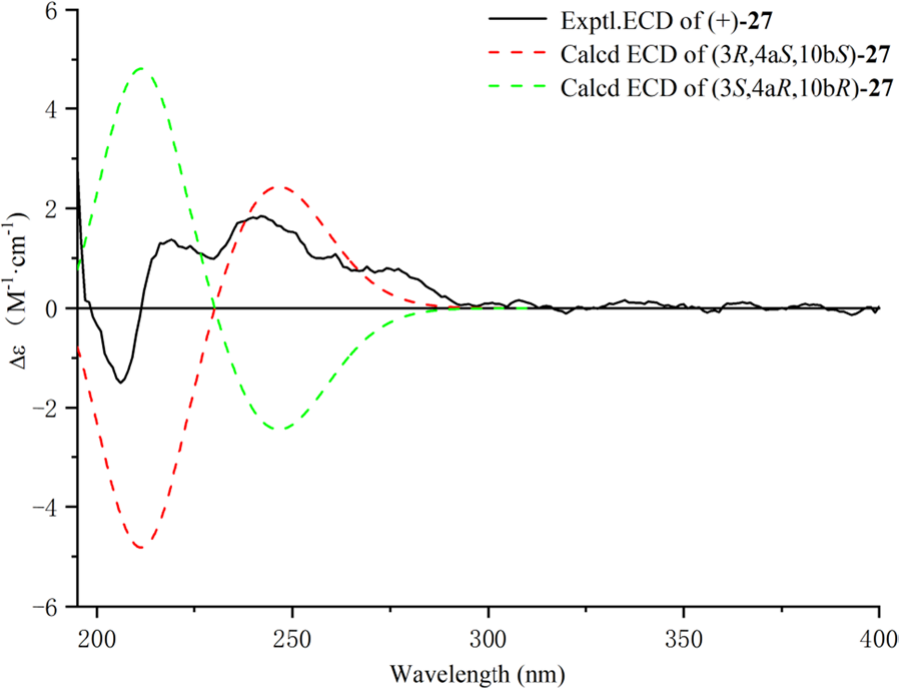
Calculated and experimental ECD spectra of **27**; σ = 0.20 eV; UV shift = − 30 nm

The relative configuration of **27** was determined by ROESY correlations (Fig. 3) of H-10b/H-4a, H-4a/H-4*β*, H-10b/H-4*β*, H-4*α*/H-3 observed, determined that the junction of B/C ring adopted a cis configuration, suggesting H-10b and H-4a were located on the same side of the ring C, and H-3 was the opposite. Hence, two stereoisomers were conceivable at that point, namely 3*R*, 4a*S*, 10b*S* and 3*S*, 4a*R*, 10b*R.* Subsequently, the absolute configuration of **27** was assigned as 3*R*, 4a*S*, and 10b*S* by comparison of the calculated and experimental ECD data in Fig. 5. Therefore, compound **27** was identified as a (3*R*, 4a*S*, 10b*S*)-2,2-dimethyl-3-hydroxy-10-*O*-*β-D*-glucoside-3,4,4a,10b-tetrahydro-2H-naphtho[1,2-b]-pyran-5H-6-one, named *Mosla chinensis* glycoside B1.

Compound **28**, white amorphous powder, possesses a molecular formula of C_20_H_24_O_7_ concluded form the HR-ESI–MS spectrum ([M + H] ^+^ *m/z* 377.1592, calcd. 377.1595). The ^1^H NMR spectrum (Table 2) of **28** revealed the signal for five aromatic protons at *δ*_H_ 7.25 (2H, s, H-2/6), 6.90 (1H, d, *J* = 8.2 Hz, H-3ʹ), 6.88 (1H, s, H-6ʹ), and 6.85 (1H, d, *J* = 8.2 Hz, H-4ʹ), two methylene groups at 4.48 (2H, t, *J* = 6.8 Hz, H-8ʹ) and 3.00 (2H, t, *J* = 6.8 Hz, H-7ʹ), and five methoxy protons at *δ*_H_ 3.85 (6H, s, H-3/5-OMe), 3.81 (3H, s, H-2ʹ-OMe), 3.79 (3H, s, H-4-OMe), and 3.78 (3H, s, H-5ʹ-OMe). Twenty carbon signals were totally observed in the spectra of ^13^C NMR and DEPT, covering eight quaternary carbons (including one ketone carbonyl at *δ*_C_ 167.5), five methyls, two methylenes (including one oxymethylene at *δ*_C_ 66.9), and five methines (Table 2). High similarity in the spectra of 1D NMR was found between **28** (Table 2) and 2-phenylethyl 2,4-dihydroxy-3-methylbenzoate [43]. The main difference between them is the presence of five methoxy groups at C-3,4,5,2ʹ,5ʹ in **28** rather than the two hydroxy groups at C-3,5 and one methyl group at C-4 in 2-phenylethyl 2,4-dihydroxy-3-methylbenzoate. The five methoxy groups were assumed to be connected to C-3,4,5,2ʹ,5ʹ, respectively, according to the HMBC correlations of C-3-OMe (*δ*_H_ 3.85) with C-3 (*δ*_C_ 56.6), C-4-OMe (*δ*_H_ 3.79) with C-4 (*δ*_C_ 56.4), C-5-OMe (*δ*_H_ 3.85) with C-5 (*δ*_C_ 56.6), and C-2ʹ-OMe (*δ*_H_ 3.81) with C-2ʹ (*δ*_C_ 61.1). Therefore, the structure of **28** was assigned as 2ʹ,5ʹ-dimethoxyphenethyl 3,4,5-trimethoxybenzoate.

Compound **29,** white amorphous powder, protonated a molecule peak at *m/z* 319.1537 [M + H] ^+^ (calcd 319.1540) corresponding to the molecular formula of C_18_H_22_O_5_. The ^1^H NMR spectrum of **29** exhibited six aromatic protons at *δ*_H_ 7.10 (2H, d, *J* = 8.1 Hz, H-6,5ʹ), 7.09 (2H, d, *J* = 8.1 Hz, H-5,6ʹ), 6.58 (1H, s, H-2ʹ), 6.33 (1H, s, H-2) and aliphatic protons at *δ*_H_ 5.50 (1H, s, H-7), 3.92 (1H, dd,* J* = 11.2, 3.7 Hz, H-8ʹa), 3.78 (1H, dd, *J* = 11.2, 2.9 Hz, H-8ʹb), 3.34 (2H, s, H-4ʹ-CH_2_), 2.57 (1H, qt, *J* = 7.1, 3.2 Hz, H-7ʹ), 2.00 (3H, s) and 1.38 (3H, d, *J* = 7.1 Hz). The ^13^C NMR spectrum of **29** exhibited 18 carbon signals, which including six quaternary carbons [covering three oxygen-bearing sp^3^ carbons at 79.4 (C-7), 69.1 (C-8ʹ), 62.9 (C-4ʹ-CH_2_)], eight methine, two methyl, and two methylene. Three ^1^H-^1^H COSY correlated systems of H-5/H-6; H-7ʹ/ H-8ʹ, and H-5ʹ /H-6ʹ were observed (Fig. 2). The results indicated that compound **29** was deduced to be a monoaromatic hydrocarbon and was structurally similar to that of 2-phenylethyl 2,4-dihydroxy-3-methylbenzoate [44]. The significant difference between them were substituent groups at C-2, 7, 3ʹ, 4ʹ, 7ʹ, there were hydrogen, hydroxy, hydroxy, hydroxymethyl and methyl at C-2, 7, 3ʹ, 4ʹ, 7ʹ of **29** rather than hydroxy, carbonyl, hydrogen, hydrogen and methyl. The deduction can be verified by the HMBC correlations of C-7ʹ (*δ*_H_ 2.75) with C-1ʹ (*δ*_C_ 138.8), C-7 (*δ*_H_ 5.50) with C-1 (*δ*_C_ 134.9), C-8ʹ (*δ*_H_ 3.92, 3.78) with C-7 (*δ*_C_ 79.4) and C-7ʹ (*δ*_C_ 31.6), C-7ʹ-Me (*δ*_H_ 1.38) with C-7ʹ (*δ*_C_ 36.1), C-4ʹ-CH_2_ (*δ*_H_ 3.34) with C-4ʹ (*δ*_C_ 127.4). Therefore, compound **29** was identified as 4-(hydroxy(2-(3-hydroxy-4-(hydroxymethyl) phenyl) propoxy) methyl)-2-methylphenol.

### Biological evaluation

#### Anti-influenza A virus activity

**Fig. 6:**
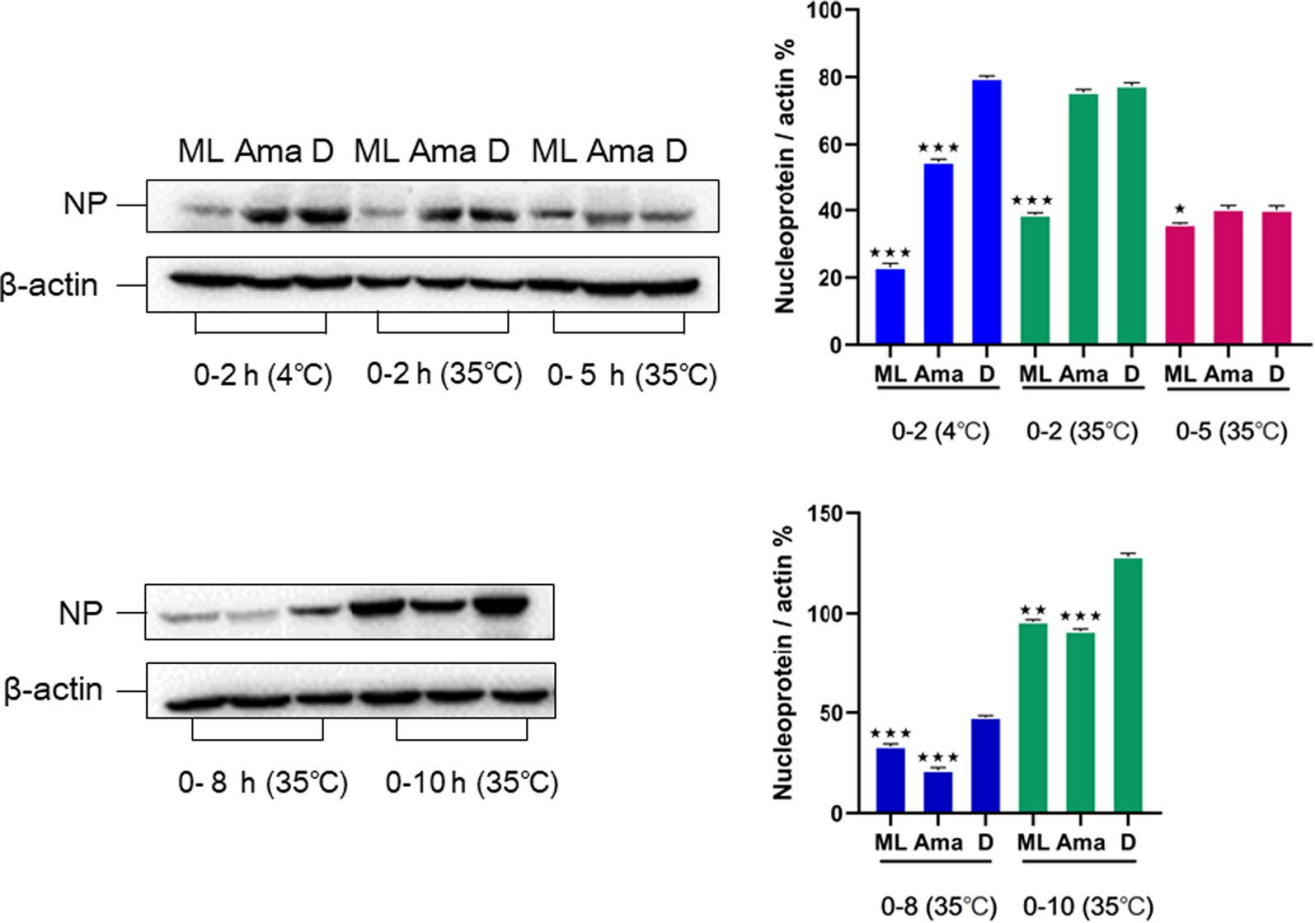
Effect of **20** (50 *μ*M) on the expression of nucleoprotein in MDCK cells. Aam was amantadine, and D stand for DMSO control. Protein expression level and gray percentage of nucleoprotein and *β*-actin in 0–2 h (4 ℃), 0–2 h, 0–5 h, 0–8 h and 0–10 h (35 ℃). (*P < 0.05, **P < 0.01, ***P < 0.001 vs IAV group, using one-way ANOVA method)

**Fig. 7:**
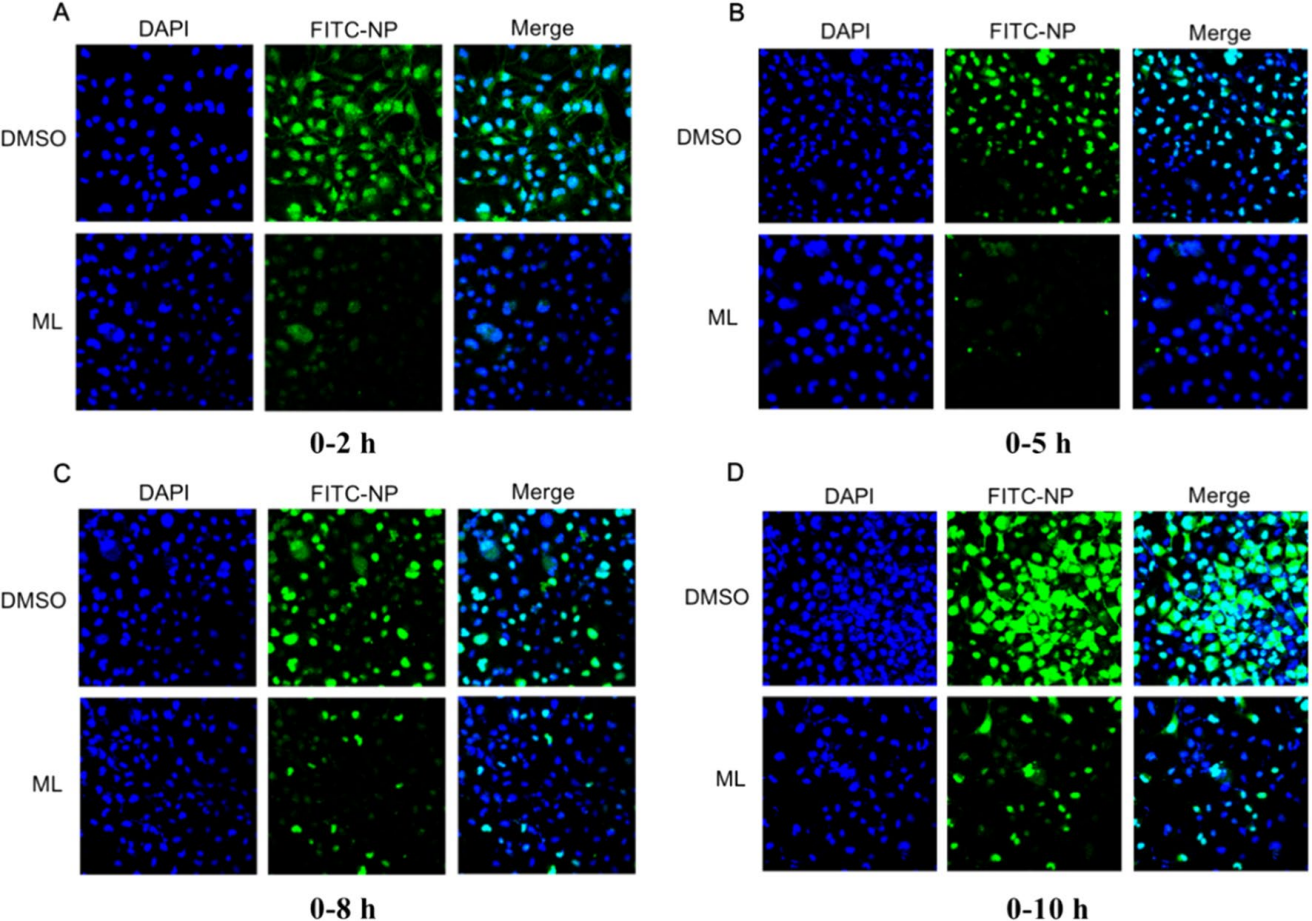
Indirect immunofluorescence microscopy. MDCK cells were infected with A/WSN/33/2009 (H1N1) and treated with **20** (50* μ*M). After 2, 5, 8, and 10 h post infection, the cells were fixed for 30 min at 4 ℃ (**A**–**D**). Cell nuclei were stained with DAPI (blue) and viewed using a fluorescence microscopy (Magnification 400 ×)

The activity of compounds (**6**–**18**, **20**–**26**, **31**–**35**) against the influenza virus was evaluated by using A/WSN/33/2009 (H1N1) infected MDCK cells. In comparison with the positive control oseltamivir with IC_50_ = 6.85 *µ*M, compound **20** exhibited significant inhibition effects of H1N1 (IC_50_ = 20.47 *µ*M); However, other compounds had no anti-influenza activity. The results of western blot analysis showed that **20** could dramatically reduce the nucleoprotein protein expression at 2, 5, and 8 h, indicating that **20** inhibits influenza virus infection by interfering with the beginning phase in the viral life cycle (Fig. 6). Furthermore, the nucleoprotein distribution in infected cells was observed by fluorescence microscopy (Fig. 7). It showed that after virus infection for 2 and 5 h, the virus population in the MDCK cells of the DMSO group was dramatically higher than that of the experimental group. This result further indicated that the influenza virus could be inhibited by compound **20**.

**Fig. 8:**
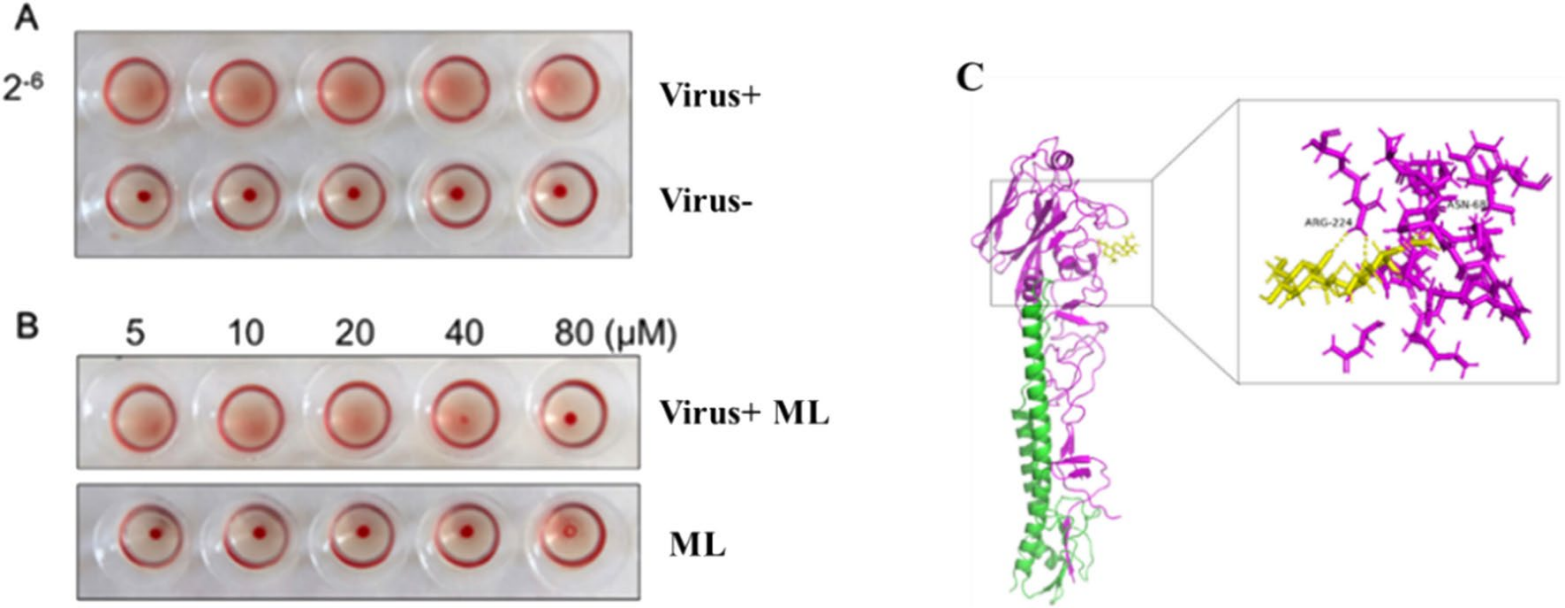
Effects of **20** on HA1. **A** The hemagglutination titer of WSN was 2^–6^, and red blood cells mixed with virus could not agglutinate. Normal red blood cells produce cell agglutination at room temperature. **B** The compound could effectively promote erythrocyte agglutination at 40 *μ*Μ and 80 *μ*Μ, the compound had no effect on red blood cells. **C** The HA1 polypeptide is colored purple, HA2 is green, and **20** is yellow. **20** can bind with HA1 residues

Glycoprotein hemagglutinin (HA) of the influenza virus has been used as a potentially important target for developing anti-influenza drugs [45]. A hemagglutinin inhibition (HI) assay was designed to check if **20** could prevent virus attachment to the cells through disturbing the connection between HA and cellular receptors. The results showed that **20** might effectively promote erythrocyte agglutination at 40 *μ*M (Fig. 8A and B). The results indicated that **20** could bind to influenza virus surface antigen HA1, inhibiting the early adsorption process of WSN. In addition, it was revealed that the docking sites with high-affinity underlying interaction could be intently related to residues ASN 68 and ARG 224 through Molecular docking (Fig. 8C). However, no binding sites between **20** and HA were observed on the receptor binding domain (RBD) of HA1 sialic acids. Therefore, it could be concluded that **20** exhibited the antiviral influence on A/WSN/33/2009 (H1N1) virus by targeting the hemagglutinin fusion machinery.

#### Anti-inflammatory activity

The influenza virus can lead to an excessive immune response and induce the production of inflammatory cytokines, such as IL-1 and IL-6 [46]. So, it would be valuable if the drugs had both antiviral and anti-inflammatory activities. Hence, we used the cells (LPS-activated RAW 264.7) to evaluate the impact of compounds (**6**–**18**, **20**–**26**, **31**–**35**) on preventing NO production. It was found that compounds **9, 22, 23**, and **25** rendered moderate activity in preventing NO production (IC_50_ = 22.78, 20.47, 27.66, and 30.14 *µ*M, respectively), in comparison with the positive control L-NMMA (IC_50_ = 21.80 *µ*M) (Table 3).

**Table 3:** Inhibitory effect of **9, 22, 23** and **25** on LSP-induced NO production in macrophages

Compound	IC_50_^b^ (*µ*M)	CC_50_^c^ (*µ*M)
9	22.78	> 50
22	20.47	> 50
23	27.66	> 50
25	30.14	> 50
L-NMMA^a^	21.80	> 50

^a^L-NMMA was used as positive control

^b^IC_50_: 50% inhibitory concentration

^c^CC_50_: 50% cytotoxic concentration

## Experimental procedures

### General experimental procedures

A Jasco digital polarimeter (DIP-370, purchased from JASCO Corporation, Tokyo, Japan) was employed to examine optical rotations. NMR spectra were monitored by a Bruker AV 600 MHz spectrometer using an internal standard (tetramethylsilane) (Bruker BioSpin Group, Germany). An API-QSTAR Pulsar (Applied Biosystem Corporation, Canada) was hired to achieve HR-ESI–MS and ESI–MS. A Shimadzu UV-2401 spectrometer (Beckman, Brea, USA) was implemented to obtain the UV spectra. Column chromatography with various gels was conducted, including 75 μM of ODS-C_18_ (YMC Co., Ltd., Japan), 75–150 μM of MCI gel (GHP20P, Mitsubishi Chemical Corporation, Tokyo, Japan), 43–63 mm of LiChroprep RP-18 (Merck), 80–100 & 200–300 mesh of silica gels (Qingdao Marine Chemical Co., Ltd., China), and Sephadex LH-20 (Amersham Biosciences AB, Uppsala, Sweden). An Agilent 1260 liquid chromatography system (Agilent, USA) was implemented for semipreparative and analytical HPLC analysis on a semipreparative Zorbax SB-C_18_ column (5 μm, 250 × 9.4 mm, 3 ml/min) and an analytical Zorbax SB-C_18_ column (5 μm, 250 × 4.6 mm, 1 ml/min), respectively. TLC was run for monitoring collected fractions on silica gel GF_254_ plates (Qingdao Marine Chemical Co., Ltd., China). The visualization of spots on the plates were conducted by using an ultraviolet lamp at the wavelength of 254 nm or by heating with H_2_SO_4_-EtOH (5%).

### Plant material

The *M. chinensis* twigs were harvested in August 2019 from Honghe Hani and Yi Autonomous Prefecture (Yunnan, China), with the authentication of Dr. Jindong Zhong (Kunming University of Science and Technology). A voucher specimen (serial number: KMUST201903) was stored at the Department of Life Science and Technology.

### Extraction and purification

The air-dried powdered of *M. chinensis* twigs (15 kg) was extracted with 70% acetone/H_2_O by refluxing for 24 h (30 L × 3 times). After filtration and evaporation procedures, the extract (1286 g) was yielded and thoroughly dissolved in H_2_O. The mixture was then extracted by petroleum ether, chloroform, ethyl acetate, and *n*-butanol, respectively. The ethyl acetate extract (145 g) was separated to four fractions (Fr. A–D) by silica gel column (20 × 300 cm) eluted with dichloromethane-methanol (1:0–0:1).

Fr. B (32.0 g) was subjected to four fractions (Fr. B-1–B-4) through MCI (90% MeOH/H_2_O) and RP-18 eluting with MeOH/H_2_O (30–100%). Fr. B-2 (1.5 g) was separated by silica gel column eluted with chloromethane-methanol (40:1–2:1) to give **9** (5.4 mg) and **23** (33.5 mg). Fr. B-3 (10.0 g) was separated to four fractions (Fr. B-3-1–B-3-5) by ODS C-18 (MeOH/H_2_O, gradient 20%–100%). Fr. B-3-2 (1.3 g) was subjected to silica gel column eluted with dichloromethane-methanol (15:0–1:1) to obtain compounds **2** (3.2 mg) and** 7** (4.2 mg). Compounds **5** (20.3 mg), **17** (2.7 mg, t_R_ = 21.2 min), **26** (4.4 mg, t_R_ = 12.5 min), and **34** (4.1 mg, t_R_ = 15.8 min) were obtained from Fr. B-3-3 (2.3 g) by Sephadex LH-20 (MeOH) and semi-preparative HPLC (85% MeOH-H_2_O, 3 ml/min). Fr. B-3-4 (1.1 g) was purified by Sephadex LH-20 (CH_2_Cl_2_–MeOH, 1:1) and semi-preparative HPLC (73% MeOH–H_2_O, 3 ml/min) to yield **24** (5.8 mg, t_R_ = 14.8 min), **25** (5.7 mg, t_R_ = 16.0 min), and **33** (5.3 mg, t_R_ = 10.6 min). Separation of Fr. B-3-5 (2.0 g) with silica gel column to obtain **22** (10.0 mg). Fr B-4 (1.0 g) was chromatographed over an ODS C-18 column (MeOH/H_2_O, 30–100%) and semi-preparative HPLC (68% MeOH–H_2_O, 3 ml/min) to give **1** (4.0 mg, t_R_ = 8.0 min), **8** (1.6 mg, t_R_ = 12.5 min)**, 11** (3.2 mg, t_R_ = 17.0 min), **12** (3.1 mg, t_R_ = 19.8 min), **31** (7.9 mg, t_R_ = 22.6 min), and **32** (10.8 mg, t_R_ = 16.0 min).

Fr. C (33.0 g) was fractioned by RP-18 (MeOH/H_2_O, gradient 30–100%) to obtain subfractions Fr. C-1–C-4. Fr. C-3 (5.4 g) was applied ODS C-18 column chromatography eluted with MeOH/H_2_O (30%–100%) and silica column (CH_2_Cl_2_-MeOH, 8:1–1:1) to give **10** (2.2 mg), **13** (9.2 mg), **20** (37.1 mg), and **35** (3.1 mg). Fr. C-4 (9.4 g) was chromatographed successively over silica gel (dichloromethane-methanol 10:1–1:1), Sephadex LH-20 gel (MeOH) and semi-preparative HPLC (65% MeOH–H_2_O, 3 ml/min) to give compounds **19** (14.1 mg, t_R_ = 18.1 min), **21** (110.0 mg, t_R_ = 16.5 min), and **14** (3.4 mg, t_R_ = 13.2 min).

Fr. D (29.0 g) was fractioned via Sephadex LH-20 gel, eluting with MeOH to afford subfractions Fr. D-1–D-4. Fr. D-2 (1.2 g) was purified by silica gel column eluted with PE/EtOAc (15:1–1:1) and Sephadex LH-20 (MeOH), successively, yield compounds **3** (5.3 mg), **4** (6.2 mg), and **6** (7.3 mg). Compounds **15** (4.1 mg, t_R_ = 14.6 min), **16** (9.3 mg, t_R_ = 17.5 min), and **27** (7.2 mg, t_R_ = 23.8 min) were obtained from Fr. D-3 (4.2 g) by Sephadex LH-20 (MeOH) and semi-preparative HPLC (52% MeOH–H_2_O, 3 ml/min). Fr. D-4 (11.2 g) was separated by silica gel column eluting with petroleum ether-ethyl acetate (10:1–1:1) to give Fr. D-4-1–D-4-3. Compound **28** (7.1 mg, t_R_ = 13.5 min) was obtained from Fr. D-4-2 (334.6 mg) by semi-preparative HPLC (85% MeOH–H_2_O, 3 ml/min). Fr. D-4-3 (2.7 g) was applied to ODS column chromatography eluted with MeOH/H_2_O (30–100%) and silica column eluting with chloromethane-methanol (30:1–1:1) to give **18** (12.1 mg), **29** (8.2 mg), and **30** (4.2 mg).

#### Acacetin 7-O-[β-d-apiofuransyl-(1ʹʹʹ → 4ʹʹ)]-β-d-xylopyranoside (1)

Amorphous powder; [*α*]25 D = − 25.1 (*c* = 0.10, MeOH); IR (KBr) *v*_max_ 3495, 3374, 2917, 2866, 1660, 1604, 1583, 1497, 1428, 1377, 1239, 1025, and 834 cm^−1^; UV (MeOH): *λ*_max_ (log *ε*) 324 (2.23) nm; HRESIMS *m/z* 571.1408 [M + Na] ^+^ (calcd for C_26_H_28_O_13_Na, 571.1428); ^1^H and ^13^C NMR data (Table 1).

#### Acacetin 7-O-[4ʹʹʹ-O-acetyl-β-d-apiofuransyl-(1ʹʹʹ → 2ʹʹ)]-6ʹʹ-O-acetyl-β-d-glucoside (2)

Amorphous powder; [*α*]25 D = − 28.0 (*c* = 0.10, DMSO); IR (KBr) *v*_max_ 3430, 2921, 2850, 1728, 1615, 1587, 1489, 1365, 1300, 1182, 1080, 987, and 829 cm^−1^; UV (MeOH): *λ*_max_ (log *ε*) 327 (3.23) nm; HRESIMS *m/z* 663.1910 [M + H] ^+^ (calcd for C_31_H_34_O_16_, 663.1920); ^1^H and ^13^C NMR data (Table 1).

#### 3ʹ, 4ʹ-Dimethoxyluteolin 7-O-[β-d-apiofuransyl-(1ʹʹʹ → 4ʹʹ)]-β-d-xylopyranoside (3)

Amorphous powder; [*α*]25 D = − 35.0 (*c* = 0.10, DMSO); IR (KBr) *v*_max_ 3389, 2928, 2865, 1700, 1659, 1608, 1514, 1382, 1338, 1298, 1183, 1123, 1085, 987, and 830 cm^−1^; UV (MeOH): *λ*_max_ (log *ε*) 326 (2.37) nm; HRESIMS *m/z* 579.1717 [M + H] ^+^ (calcd for C_27_H_30_O_14_, 579.1714); ^1^H and ^13^C NMR data (Table 1).

#### 9ʹ,9ʹʹ-dimethyl clinopodic acid C (19)

White amorphous powder; [*α*]25 D =  + 100.0 (*c* = 0.10, MeOH); IR (KBr) *v*_max_ 3431, 3040, 2954, 2925, 2851, 1741, 1607, 1585, 1506, 1440, 1269, 1117, 978, 858, and 812 cm^−1^; UV (MeOH): *λ*_max_ (log *ε*) 327 (3.14) nm; HRESIMS *m/z* 589.1293 [M + Na] ^+^ (calcd for C_29_H_26_O_12_Na, 589.1322); ^1^H and ^13^C NMR data (Table 2).

#### Mosla chinensis glycoside B1 (27)

White amorphous powder; [*α*]25 D = -37.8 (*c* = 0.10, MeOH); IR *v*_max_ 3501, 3407, 3305, 2873, 1610, 1518, 1365, 1035, 826, and 714 cm^−1^; ECD (MeOH) λ_max_ (Δ*ε*) 211 (-4.82), 247 (+ 2.43) nm; UV (MeOH): *λ*_max_ (log *ε*) 292 (2.57) nm; HRESIMS, *m/z* 447.1638 [M + Na] ^+^ (calcd for C_21_H_28_O_9_Na_,_ 447.1631); ^1^H and ^13^C NMR data (as shown in Table 2).

#### 2,5-dimethoxyphenethyl 3,4,5-trimethoxybenzoate (28)

Colorless crystal; [*α*]25 D = − 12.3 (*c* = 0.10, MeOH); IR *v*_max_ 3445, 2933, 2843, 1715, 1592, 1512, 1460, 1415, 1334, 1228, 1127, 1028, 1002, 862, 807, and 764 cm^−1^; UV (MeOH) *λ*_max_ (log* ε*) 310 (2.78) nm; HRESIMS, *m/z* 377.1592 [M + H] ^+^ (calcd for C_**20**_H_24_O_7_, 377.1595); ^1^H and ^13^C NMR data (as shown in Table 2).

#### 4-(hydroxy(2-(3-hydroxy-4-(hydroxymethyl) phenyl) propoxy) methyl)-2-methy- lphenol (29)

White amorphous powder; [*α*]25 D = − 12.4 (*c* = 0.10, MeOH); IR *v*_max_ 3415, 2921, 2853, 1512, 1456, 1407, 1263, 1170, 1063, and 1023 cm^−1^; UV (MeOH): *λ*_max_ (log *ε*) 251 (2.25) nm; HRESIMS, *m/z* 319.1537 [M + H] ^+^ (calcd for C_18_H_22_O_5_, 319.1540); ^1^H and ^13^C NMR data (as shown in Table 2).

### Acid hydrolysis and determination of the absolute configuration of sugars

Based on the method reported by Wu et al. [47], the *D* glucopyranose configurations in compounds **1–3** and **27** were measured. Compounds **1–3** and **27** (1 mg per compound) were individually mixed with 3 ml HCl (2 M). Each mixture was boiled for 4 h at 100 ℃. After neutralization with NaHCO_3_, the mixture was treated by EtOAc. Subsequently, the H_2_O layer was evaporated and dissolved in DMSO (1.0 ml) before the acetic anhydride (40 μL) and 1-Methylimidazole (20 μL). After the reaction, extracted with EtOAc and analysed by GC. Monosaccharide compositions in compounds were identified by coeluting with authentic monosaccharide.

### ECD quantification

The conformational structures of compounds were achieved from Chem3D modeling and ROESY spectra. In terms of the conformations, low-energy conformers of **27** were created via CONFLEX software by using an energy window (10 kcal/mol, MMFF94S) [48]. Density functional theory (DFT) method was employed to optimize the selected conformers in MeOH at the B3LYP/6-31 G (d) level [49]. Geometry optimizations and predictions of the conformersʹ ECD spectra were conducted by TD-DFT-B3LYP/6-311G (2d, p) level using a solvent (IEFPCM solvent model for methanol) [50]. SpecDis 1.71 was hired to generate the predicted curves of ECD, and Gaussian 16 package was applied to all predictions [51]. After UV correction, compound **27** spectrum was weighted using the Boltzmann distribution.

### Anti-influenza virus assay

Based on the approach described by Dang et al. [52], anti-influenza virus assay was carried out. Briefly, prior to infection, MDCK cells (8 × 10^3^ cells/well) were cultivated for 24 h in 96-well plates, and the medium was removed. The mixture of compounds (at 3.125, 6.25, 12.5, 25, and 50 μM) and H1N1 virus was cultured at ambient temperature for 15 min and then transferred to the plates containing MDCK cells. The plates were stored at 37 °C with 5% CO_2_ for 48 h. Subsequently, the antiviral activity was quantified using microscopy. The obtained antiviral activity was verified using the CellTiter-Glo luminescent cell viability assay (Promega, #G7570). Each compound’s cytotoxicity was evaluated through incubating with uninfected MDCK cells for 48 h [53, 54].

### Western blot assay

Influenza virus-infected MDCK cells were added with compound **20** (50 *μ*M) at distinct time points (2, 5, 8, and 10 h). Cell lysates were harvested, and proteins from the supernatant were obtained [55]. After being electrophorized on a 12% SDS-PAGE electrophorized gel, protein extracts were distributed on a PVDF membrane and then cultivated for 1 h in blocking media (5% nonfat milk) at ambient temperature. Immunoblotting was performed using antibodies: anti-*β*-actin (SC-47778 (Catalog No.), Santa Cruz) and anti-nucleoprotein (EPR25683 (Abcam No.), GeneTex). The target proteins were visualized by chemiluminescence (ECL, Beyotime).

### Immunofluorescence assay

MDCK cells (1 × 10^5^ cells) were loaded into each well of 24-well plates. The plates were kept under 5% CO_2_ condition at 37 °C. As the cells increased by 50%, the cells were added and cultured with A/WSN/33/2009 (H1N1) virus (MOI = 0.1) for 2 h. After the removal of the supernatant, the cells were rinsed two times using PBS. Subsequently, the **20** was supplemented to cells and stored under conditions of 5% CO_2_ and 37 °C. At 2, 5, 8, and 10 h of incubation, the cells were fixed using PFA in PBS (4%, Beyotime Biotechnology) at refrigerated temperature for 10 min. The fixed cells were then permeabilized for 10 min at room temperature using 0.1% Triton X-100 in PBS and blocked for 1 h at 37 °C using 3% BSA in PBS. Afterward, the treated cells were first stored at 4°C overnight supplemented with nucleoprotein antibody diluted in 3% BSA (1:250, Abcam, CA, USA) and then cultured at ambient temperature for 1 h with fluorescein isothiocyanate (FITC)-labeled secondary antibody diluted in 3% BSA (1:250). The nucleus in cells was stained by DAPI for 10 min at ambient temperature. After staining, the fluorescence was examined by an inverted fluorescence microscope (Nikon A1R/A1, Shanghai, Japan) [56].

### Hemagglutination inhibition assay

*Hemagglutination inhibition* (HAI) test was utilized to evaluate the activity of **20** against HA-mediated avian RBCs hemagglutination [57]. Briefly, **20** (10, 20, 40, and 80 *μ*M) with influenza A/WSN/33/2009 (H1N1) (2^–6^ hemagglutination titer) was supplemented into 96-well plates and cultured under an ambient condition for 1 h. Afterward, 1% chicken RBCs saline solution (50 *μ*L) was loaded into each well. After incubation for 30 min at ambient temperature, the hemagglutination was examined.

### Molecular docking

The protein structure of H1N1 HA (PDB ID: 6CFG) was achieved from the RCSB protein data bank. Chemdraw3D was hired to construct **20**’s 3D structures. The docking procedures were provided by the AutoDock Software with a graphics interface (AutoGrid/AutoDock 4.2.6 and Vina). With the AutoDock tools, the deletion of all water molecules was executed, while the refined model was added with the polar hydrogen atoms and charges. Then, docking was conducted using AutoDock/Vina based on the HA information and the grid box characteristics of the studied compound in the configuration file. In the process of docking, the **20** structure and HA protein structure were regarded as rigid [58, 59].

### Nitric oxide production assay

Based on the previously reported approach [60, 61], NO production in cells was assayed. Cells were loaded into 96-well plates (8×10^4^/ well). The cells were treated by compounds (at 3.125, 6.25, 12.5, 25, and 50 *μ*M) for 1 h, supplemented with LPS (1 *μ*g/ml), and cultivated for 24 h. After treatment, a microplate reader (Thermo Fisher Scientific, Massachusetts, USA) was hired to quantify the absorbance values with the wavelength of 540 nm. The positive and negative controls were separately L-NMMA and DMSO.

## Conclusions

This work systematically explored the phytochemical characteristics of thirty-five compounds extracted from *M. chinensis* twigs. The compounds included three unreported flavonoids (**1**–**3**), one undescribed phenylpropanoid (**19**) and three new monoaromatic hydrocarbons (**27–29**), and 28 known compounds*.* Compound **27**’s absolute configuration was interpreted and visualized with the assistance of ECD calculation. Compound **20** exhibited the most significant activity against A/WSN/33/2009 (H1N1) virus (IC_50_ = 20.47 *μ*M). Further research showed that **20** could bind to influenza virus surface antigen HA1 and inhibit the early adsorption process of the influenza A/WSN/33/2009 (H1N1) virus strain. Furthermore, compounds** 9**, **22**, **23**, and **25** displayed moderate inhibitory effects on the NO expression in LPS inducing Raw 264.7 cells with IC_50_ values of 22.78, 20.47, 27.66, and 30.14 *µ*M, respectively.

The effect of *M. chinensis* on influenza virus infection, controlling the adsorption of virus and excessive inflammatory reaction in the infection process. Our findings will enrich the study of the structural diversity of *M. chinensis* and provide insights into understanding the plant’s anti-influenza function, which may launch scientific basis for the following research about the development of antiviral beverages and resources utilization of *M*. *chinensis*.

### Supplementary Information

**Additional file 1.** It includes 1D NMR, 2D NMR, HRESIMS, UV, IR, ECD, and computational data of compounds 1–3, 19, 27~28 and the GC analysis of sugar of compound 1~3, and 27.

## Data Availability

The datasets used or analysed during the current study are available from the corresponding author on reasonable request.

## References

[1] Heo JY, Song JY, Noh JY, Choi MJ, Yoon JG, Lee SN, Cheong HJ, Kim WJ (2018). Effects of influenza immunization on pneumonia in the elderly. Hum Vacc Immunother.

[2] Schanzer DL, Langley JM, Tam TWS (2006). Hospitalization attributable to influenza and other viral respiratory illnesses in Canadian children. Pediatr Infect Dis J.

[3] Li Z, Wang H, Wang FX, Li HY, Cao F, Luo DQ, Zhang Q, Chen FL (2022). Isolation of essential oil from *Mosla chinensis* Maxim by surfactant-enzyme pretreatment in high-solid system and evaluation of its biological activity. Ind Crop Prod.

[4] National Pharmacopoeia Committee. Pharmacopoeia of People’s Republic of China. Part 1. Medical Science and Technology Press: Beijing, 2015; 259–60.

[5] Cao L, Si JY, Liu Y, Sun H, Jin W, Li Z, Zhao XH, Pan RL (2009). Essential oil composition, antimicrobial and antioxidant properties of *Mosla chinensis* Maxim. Food Chem.

[6] Liu MT, Luo FY, Zeng JG (2020). Composition analysis of essential oil of *Mosla chinensis* Maxim and its antibacterial and antioxidant activity. Chin Tradit Patent Med.

[7] Lin CL, Cai JZ, Lin GY (2012). Chemical constituent study of volatile oils from the *Mosla chinensis* Maxim in Zhejiang Province. Chin Arch Tradit Chin Med.

[8] Feng Y, Liu J (2009). Effects of volatile oil from *Mosla chinensis* Maxim on bacteriostasis and immune response. Amino Acids Biotic Resour.

[9] Ge B, Lu XY, Jiang HM (2005). Study on antibacterial effect of volatile oil of *Mosla chinensis* Maxim in vitro. Chin J Tradit Veterinary Sci.

[10] Zhang XX, Wu QF, Yan YL, Zhang FL (2018). Inhibitory effects and related molecular mechanisms of total flavonoids in *Mosla chinensis* Maxim against H1N1 influenza virus. Inflamm Res.

[11] Zhang L, Yang LY, Li RT, Yu F, Zhong JD (2022). A new prenylated 3-benzoxepin derivative with anti-influenza A virus activity from *Elsholtzia penduliflora*. Nat Prod Res.

[12] Yang LY, Du JC, Li RT, Yu F, Zhong JD (2021). Bodiniosides S-Y, seven new triterpenoid saponins from *Elsholtzia bodinieri* and their anti-Influenza activities. Molecules.

[13] Qiao Y, Sun WW, Wang JF, Zhang JD (2014). Flavonoids from *Podocarpus macrophyllus* and their cardioprotective activities. J Asian Nat Prod Res.

[14] Sugimoto S, Yamano Y, Desoukey SY, Katakawa K, Matsunami K (2019). Isolation of sesquiterpene–amino acid conjugates, onopornoids A-D, and a flavonoid glucoside from *Onopordum alexandrinum*. J Nat Prod.

[15] Sinha NK, Seth KK, Pandey VB, Dasgupta B, Shah AH (1981). Flavonoids from the flowers of *Clerodendron infortunatum*. Planta Med.

[16] Seo YH, Trinh TA, Ryu SM, Kim HS, Lee J (2020). Chemical constituents from the aerial parts of *Elsholtzia ciliata* and their protective activities on glutamate-induced HT22 Cell Death. J Nat Prod.

[17] Perry NB, Foster LM (1994). Antiviral and antifungal flavonoids, plus a triterpene, from *Hebe cupressoides*. Planta Med.

[18] Hori K, Satake T, Saiki Y, Tanaka N, Murakami T, Chen CM (1988). Chemical and chemotaxonomical studies of Filices. LXXIV. The novel flavanone glycosides of *Pyrrosia linearfolia* (HOOK.) Ching. J Pharm Soc Jpn.

[19] Zhang XL, Guo YS, Wang CH, Li GQ, Xu JJ, Chung HY, Ye WC, Li YL, Wang GC (2014). Phenolic compounds from *Origanum vulgare* and their antioxidant and antiviral activities. Food Chem.

[20] Lai GF, Zhu XD, Luo SD, Wang YF (2008). Chemical constituents from *Elsholtzia rugulos*. Chin Tradit Herb Drugs.

[21] Oyama KI, Kondo T (2004). Total synthesis of apigenin 7,4 prime-di-O-*β*-glucopyranoside, a component of blue flower pigment of *Salvia* patens, and seven chiral analogues. Tetrahedeon.

[22] Besson E, Chopin J (1983). Sugar ring isomerization in C-arabinosyl flavones. Phytochemistry.

[23] Wang XF, Li H, Jiang K, Wang QQ, Zheng YH, Wei T, Tan CH (2018). Anti-inflammatory constituents from *Perilla frutescens* on lipopolysaccharide-stimulated RAW 264.7 cells. Fitoterapia.

[24] Formisano C, Rigano D, Senatore F, Bancheva S, Maggio A, Rosselli S, Bruno M (2012). Flavonoids in subtribe centaureinae (Cass.) Dumort. (Tribe Cardueae, Asteraceae): distribution and 13C-NMR spectral data. Chem Biodiversity.

[25] Kong CH, Xu XH, Hu F, Chen XH, Ling B, Tan ZW (2002). Using specific secondary metabolites as markers to evaluate allelopathic potentials of rice varieties and individual plants. Chin Sci Bull.

[26] Zhang Q, Guilhon CC, Fernandes PD, Boylan F (2014). Antinociceptive and anti-inflammatory activities of *Elsholtzia ciliata* (Thunb.) Hyl. (Lamiaceae) extracts. Planta Med.

[27] Chen XF, Ma GX, Huang Z, Wu TY, Xu XD, Zhong XM (2017). Identification of water-soluble phenolic acids from *Clerodendranthus spicatus*. Chin Tradit Herb Drugs.

[28] Tsai SF, Lee SS (2014). Neolignans as xanthine oxidase inhibitors from *Hyptis rhomboides*. Phytochemistry.

[29] Lee C, Kim J, Lee H, Lee S, Kho Y (2001). Two new constituents from *Isodon excisus* and their evaluation in an apoptosis inhibitioni assay. J Nat Prod.

[30] Su D, Tang W, Hu Y, Liu Y, Yu S, Ma S, Qu J, Yu D (2008). Lignan glycosides from *Neoalsomitra integrifoliola*. J Nat Prod.

[31] Gu QC, Yin ZK, Feng ZM, Jiang JS, Zhang X, Zhang PC, Yang YN (2020). Three 11,12-seco-tanshinone derivatives from the rhizomes of *Salvia miltiorrhiza*. J Asian Nat Prod Res.

[32] Kato H, Li W, Koike M, Wang Y, Koike K (2010). Phenolic glycosides from *Agrimonia pilosa*. Phytochemistry.

[33] Dhingra MS, Dhingra S, Kumria R, Chadha R, Singh T, Kumar A, Karan M (2014). Effect of trimethylgallic acid esters against chronic stress-induced anxiety-like behavior and oxidative stress in mice. Pharmacol Rep.

[34] Takeda Y, Tomonari M, Arimoto S, Masuda T, Otsuka H, Matsunami K, Honda G, Ito M, Takaishi Y, Kiuchi F, Khodzhimatov OK, Ashurmetov OA (2008). A new phenolic glucoside from an Uzbek medicinal plant, Origanum tyttanthum. J Nat Med.

[35] Bravo JA, Sauvain M, Gimenez A, Munoz V, Callapa J, Le L, Massiot G, Lavaud C (1999). Bioactive phenolic glycosides from *Amburana cearensis*. Phytochemistry.

[36] Koike K, Li W, Liu LJ, Hata E, Nikaido T (2005). New phenolic glycosides from the seeds of *Cucurbita moschata*. Chem Pharma Bull.

[37] Guetchueng ST, Nahar L, Ritchie KJ, Ismail FMD, Dempster NM, Nnanga EN, Sarker SD (2020). Phenolic compounds from the leaves and stem bark of *Pseudospondias microcarpa* (A. Rich.) Engl. (Anacardiaceae). Biochem Syst Ecol.

[38] Tagousop CN, Ngnokam D, Harakat D (2017). Three new flavonoid glycosides from the aerial parts of *Graptophyllum grandulosum* Turril (Acanthaceae). Phytochem Lett.

[39] Xu JZ, Zhang SS, Qu HB (2010). Chemical constituents from *Viola yedoensis*. Chin Tradit Herb Drugs.

[40] Miyazawa M, Hisama M (2003). Antimutagenic activity of flavonoids from *Chrysanthemum morifolium*. Biosci Biotechnol Biochem.

[41] Murata T, Sasaki K, Sato K, Yoshizaki F, Yamada H, Mutoh H, Umehara K, Miyase T, Warashina T, Aoshima H (2009). Matrix metalloproteinase-2 inhibitors from *Clinopodium chinense* var. parviflorum. J Nat Prod.

[42] Zhong M, Sun G, Zhang X, Sun G, Xu X, Yu S (2012). A New prenylated naphthoquinoid from the aerial parts of *Clinopodium chinense* (Benth.) O. Kuntze. Molecules.

[43] Hiipakka RA, Zhang HZ, Dai W, Dai Q, Liao S (2002). Structure-activity relationships for inhibition of human 5alpha-reductases by polyphenols. Biochem Pharmacol.

[44] Liu F, Zhong J, Zhou Y, Gao Z, Walsh PJ, Wang X, Ma S, Hou S, Liu S, Wang M, Wang M, Bian Q (2018). Cobalt-catalyzed enantioselective negishi cross-coupling of racemic *α*-Bromo esters with arylzincs. Chemistry.

[45] Mair CM, Ludwig K, Herrmann A, Sieben C (2014). Receptor binding and pH stability—how influenza A virus hemagglutinin affects host-specific virus infection. Bba-Biomembranes.

[46] Tavares LP, Teixeira MM, Garcia CC (2017). The inflammatory response triggered by influenza virus: a two edged sword. Inflamm Res.

[47] Wu ST, Li F, Wang YX, Yu BH, Ma CL, Qiu HQ, Wang GS (2022). Phenylpropanoids from *Brachybotrys paridiformis* Maxim. Ex Oliv. and their anti-HBV activities. Phytochemistry.

[48] Fujihara T, Obora Y, Tokunaga M, Sato H, Tsuji Y (2005). Dendrimer N-heterocyclic carbene complexes with rhodium(I) at the core. Chem Commun.

[49] Li JC, Dai WF, Liu D, Jiang MY, Zhang ZJ, Chen XQ, Chen CH, Li RT, Li HM (2020). Bioactive ent-isopimarane diterpenoids from *Euphorbia neriifolia*. Phytochemistry.

[50] Chen X, Cao YG, Ren YJ, Liu YL, Fan XL, He C, Li XD, Ma XY, Zheng XK, Feng WS (2022). Ionones and lignans from the fresh roots of *Rehmannia glutinosa*. Phytochemistry.

[51] Wang P, Liu F, Yang X, Liang Y, Li S, Su G, Jin DQ, Ohizumi Y, Xu J, Guo Y (2017). Clerodane diterpenoids from *Scutellaria formosana* with inhibitory effects on NO production and interactions with iNOS protein. Phytochemistry.

[52] Dang Z, Jung K, Zhu L, Lai W, Xie H, Lee KH, Huang L, Chen CH (2014). Identification and synthesis of quinolizidines with anti-influenza a virus activity. ACS Med Chem Lett.

[53] Vanderlinden E, Göktas F, Cesur Z, Froeyen M, Reed ML, Russell CJ, Cesur N, Naesens L (2010). Novel inhibitors of influenza virus fusion: structure-activity relationship and interaction with the viral hemagglutinin. J Virol.

[54] Jones JC, Turpin EA, Bultmann H, Brandt CR (2006). Inhibition of influenza virus infection by a novel antiviral peptide that targets viral attachment to cells. J Virol.

[55] Zhang YZ, Naguro I, Herr AE (2019). In situ single-cell western blot on adherent cell culture. Angew Chem Int Ed Engl.

[56] Liang XX, Zhang XJ, Zhao YX, Feng J, Zeng JC, Shi QQ, Kaunda JS, Li XL, Wang WG, Xiao WL (2022). Aspulvins A-H, aspulvinone analogues with SARS-CoV-2 M(pro) inhibitory and anti-inflammatory activities from an Endophytic Cladosporium sp. J Nat Prod.

[57] Zhang T, Lo CY, Xiao M, Cheng L, Pun Mok CK, Shaw PC (2020). Anti-influenza virus phytochemicals from *Radix Paeoniae* Alba and characterization of their neuraminidase inhibitory activities. J Ethnopharmacol.

[58] Shi WZ, Jiang LZ, Song GP, Wang S, Xiong P, Ke CW (2020). Study on the antiviral activities and hemagglutinin-based molecular mechanism of novel chlorogenin 3-O-β-chacotrioside derivatives against H5N1 subtype viruses. Viruses.

[59] Ye M, Liao Y, Wu L, Qi W, Choudhry N, Liu Y, Chen W, Song G, Chen J (2020). An oleanolic acid derivative inhibits hemagglutinin-mediated entry of influenza A virus. Viruses.

[60] Lee JW, Jin Q, Jang H, Lee D, Han SB, Kim Y, Hong JT, Lee MK, Hwang BY (2016). Jatrophane and ingenane-type diterpenoids from Euphorbia kansui inhibit the LPS-induced NO production in RAW 264.7 cells. Bioorg Med Chem Lett.

[61] Cao L, Li RT, Chen XQ, Xue Y, Liu D (2016). Neougonin A inhibits lipopolysaccharide-induced inflammatory responses via downregulation of the NF-kB signaling pathway in RAW 264.7 macrophages. Inflammation.

