# Supplementary material for: Antiviral and anti-inflammatory activities of chemical constituents from twigs of Mosla chinensis Maxim
Source: Nat Prod Bioprospect. 2024 May 1;14(1):26. doi: 10.1007/s13659-024-00448-w (PMC11063020; doi:10.1007/s13659-024-00448-w)
Supplement: Supplementary file 1 — Additional file 1. It includes 1D NMR, 2D NMR, HRESIMS, UV, IR, ECD, and computational data of compounds 1–3, 19, 27~28 and the GC analysis of sugar of compound 1~3, and 27. [file 13659_2024_448_MOESM1_ESM.docx]

**^^[[1]](#footnote-1)^^ SUPPLEMENTARY MATERIAL**

**Antiviral and anti-inflammatory activities of chemical constituents from twigs of *Mosla chinensis* Maxim**

Shi-Yan Feng^1^, Na Jiang^1^, Jia-Ying Yang^1^, Lin-Yao Yang, Jiang-Chao Du, Xuan-Qin Chen, Dan Liu, Rong-Tao Li, Jin-Dong Zhong^*^

*Faculty of Life Science and Technology, Kunming University of Science and Technology, Kunming 650500, Yunnan, PR China*

*Corresponding author. Faculty of Life Science and Technology, Kunming University of Science and Technology, 727 Jingming South Road, Chenggong District, Kunming 650500, Yunnan, P. R. China. Tel: +86-871-65920569, Fax: +86-871-65920570

**Corresponding author.

*E-mail address*: [jindongzhongkm@163.com](mailto:jindongzhongkm@163.com) (J.-D. Zhong)

**List of Contents**

[Figure S1](#_Toc44082032) ^[1](#_Toc44082032)^[H NMR spectrum of compound](#_Toc44082032) **[1](#_Toc44082032)**[(600 MHz, DMSO-](#_Toc44082032)*[d](#_Toc44082032)*_[6](#_Toc44082032)_[)](#_Toc44082032)

[Figure S2 ^13^C NMR spectrum of compound **1**(150 MHz, DMSO-*d*_6_)](#_Toc44082033)

[Figure S3 HSQC spectrum of compound **1**](#_Toc44082034)

[Figure S4 HMBC spectrum of compound **1**](#_Toc44082035)

[Figure S5 ^1^H-^1^H COSY spectrum of compound **1**](#_Toc44082035)

[Figure S6 ROSEY spectrum of compound **1**](#_Toc44082035)

[Figure S7 HRESIMS spectrum of compound **1**](#_Toc44082036)

[Figure S8 UV spectrum of compound **1**](#_Toc44082036)

[Figure S9 IR spectrum of compound **1**](#_Toc44082036)

[Figure S10 ^1^H NMR spectrum of compound **2**(600 MHz, DMSO-*d_6_*)](#_Toc44082038)

[Figure S11 ^13^C NMR spectrum of compound **2**(150 MHz, DMSO-*d_6_*)](#_Toc44082039)

[Figure S12 HSQC spectrum of compound **2**](#_Toc44082040)

[Figure S13 HMBC spectrum of compound **2**](#_Toc44082041)

[Figure S14 ^1^H-^1^H COSY spectrum of compound **2**](#_Toc44082035)

[Figure S15 ROSEY spectrum of compound **2**](#_Toc44082035)

[Figure S16 HRESIMS spectrum of compound **2**](#_Toc44082042)

[Figure S17 UV spectrum of compound **2**](#_Toc44082036)

[Figure S18 IR spectrum of compound **2**](#_Toc44082036)

[Figure S19 ^1^H NMR spectrum of compound **3** (600 MHz, DMSO-*d_6_*)](#_Toc44082044)

[Figure S20 ^13^C NMR spectrum of compound **3** (150 MHz, DMSO-*d_6_*)](#_Toc44082045)

[Figure S21 HSQC spectrum of compound **3**](#_Toc44082046)

[Figure S22 HMBC spectrum of compound **3**](#_Toc44082047)

[Figure S23 HRESIMS spectrum of compound **3**](#_Toc44082048)

[Figure S24 UV spectrum of compound **3**](#_Toc44082036)

[Figure S25 IR spectrum of compound **3**](#_Toc44082036)

[Figure S26 ^1^H NMR spectrum of compound **19** (600 MHz, DMSO-*d*_6_)](#_Toc44082050)

[Figure S27 ^13^C NMR spectrum of compound **19** (150 MHz, DMSO-*d*_6_)](#_Toc44082051)

[Figure S28 HSQC spectrum of compound **19**](#_Toc44082052)

[Figure S29 HMBC spectrum of compound **19**](#_Toc44082053)

[Figure S30 ^1^H-^1^H COSY spectrum of compound **19**](#_Toc44082035)

[Figure S31 ROSEY spectrum of compound **19**](#_Toc44082035)

[Figure S32 HRESIMS spectrum of compound **19**](#_Toc44082054)

Figure S33 UV spectrum of compound **19**

Figure S34 IR spectrum of compound **19**

[Figure S35 ^1^H NMR spectrum of compound **27** (600 MHz, DMSO-*d*_6_)](#_Toc44082056)

[Figure S36 ^13^C NMR spectrum of compound **27** (150 MHz, DMSO-*d*_6_)](#_Toc44082057)

[Figure S37 HSQC spectrum of compound **27**](#_Toc44082058)

[Figure S38 HMBC spectrum of compound **27**](#_Toc44082059)

[Figure S39 ^1^H-^1^H COSY spectrum of compound **27**](#_Toc44082035)

[Figure S40 ROSEY spectrum of compound **27**](#_Toc44082035)

[Figure S41 HRESIMS spectrum of compound **27**](#_Toc44082060)

Figure S42 UV spectrum of compound **27**

Figure S43 IR spectrum of compound **27**

[Figure S44 ^1^H NMR spectrum of compound **28** (600 MHz, DMSO-*d*_6_)](#_Toc44082062)

[Figure S45 ^13^C NMR spectrum of compound **28** (150 MHz, DMSO-*d*_6_)](#_Toc44082063)

[Figure S46 HSQC spectrum of compound **28**](#_Toc44082064)

[Figure S47 HMBC spectrum of compound **28**](#_Toc44082065)

[Figure S48 HRESIMS spectrum of compound **28**](#_Toc44082066)

Figure S49 UV spectrum of compound **28**

Figure S50 IR spectrum of compound **28**

[Figure S51 ^1^H NMR spectrum of compound **29** (600 MHz, DMSO-*d*_6_)](#_Toc44082068)

[Figure S52 ^13^C NMR spectrum of compound **29** (150 MHz, DMSO-*d*_6_)](#_Toc44082069)

[Figure S53 HSQC spectrum of compound **29**](#_Toc44082070)

[Figure S54 HMBC spectrum of compound **29**](#_Toc44082071)

[Figure S55 ROSEY spectrum of compound **29**](#_Toc44082035)

[Figure S56 HRESIMS spectrum of compound **29**](#_Toc44082072)

Figure S57 UV spectrum of compound **29**

Figure S58 IR spectrum of compound **29**

Figure S59 GC spectrum of standards

Figure S60 GC spectrum of compound **1**

Figure S61 GC spectrum of compound **2**

Figure S62 GC spectrum of compound **3**

Figure S63 GC spectrum of compound **27**

Table S1: The energy distribution and boltzmann weights of each conformer of compound **27**

Table S2-10: The list of conformers with their structures and atomic coordinates calculated ECD data for compound **27**

Figure 1 ^1^H NMR spectrum of compound **1** (600 MHz, DMSO-*d*_6_)


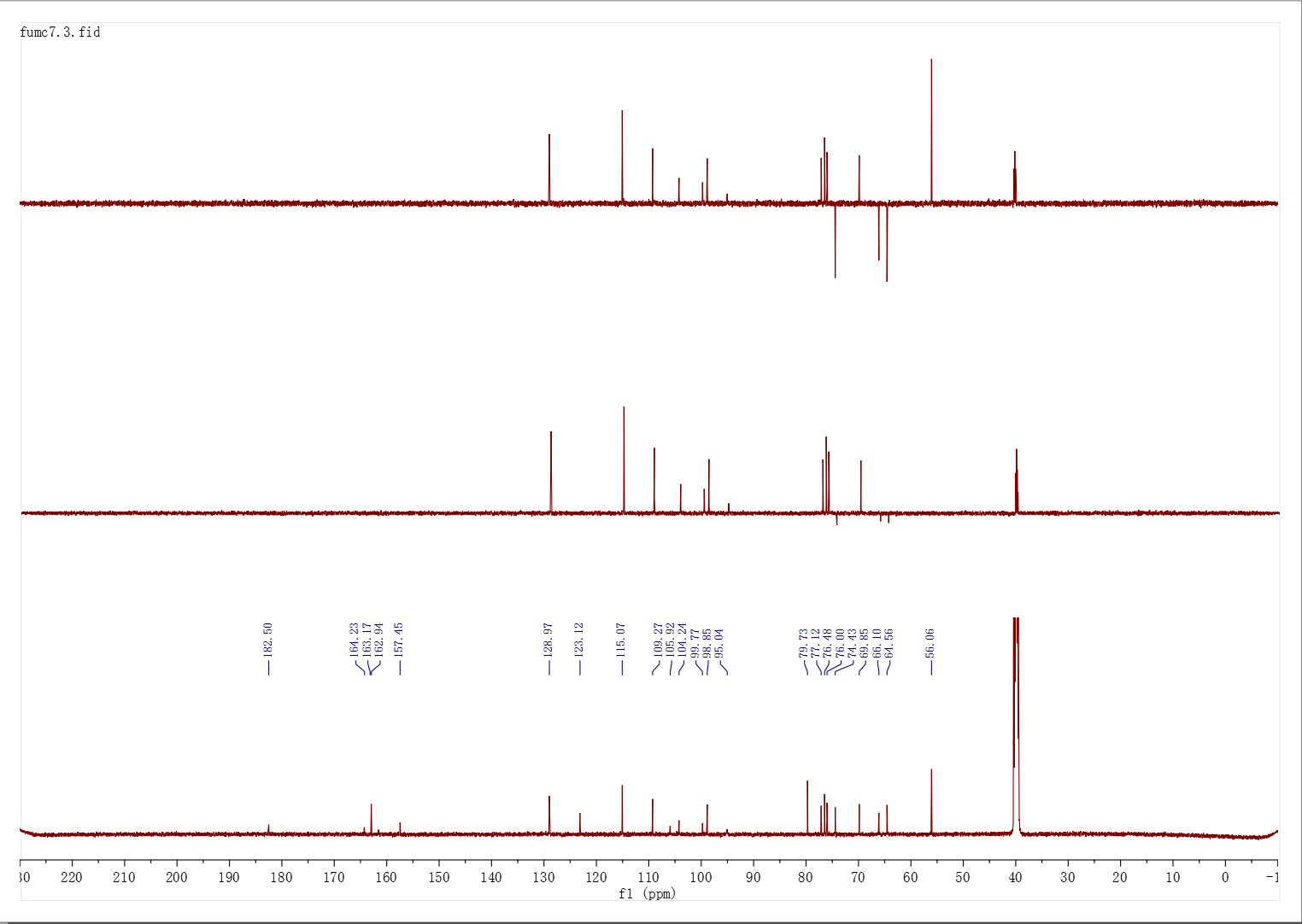


Figure 2 ^13^C NMR spectrum of compound **1** (150 MHz, DMSO-*d*_6_)

Figure 3 HSQC spectrum of compound **1**

Figure 4 HMBC spectrum of compound **1**


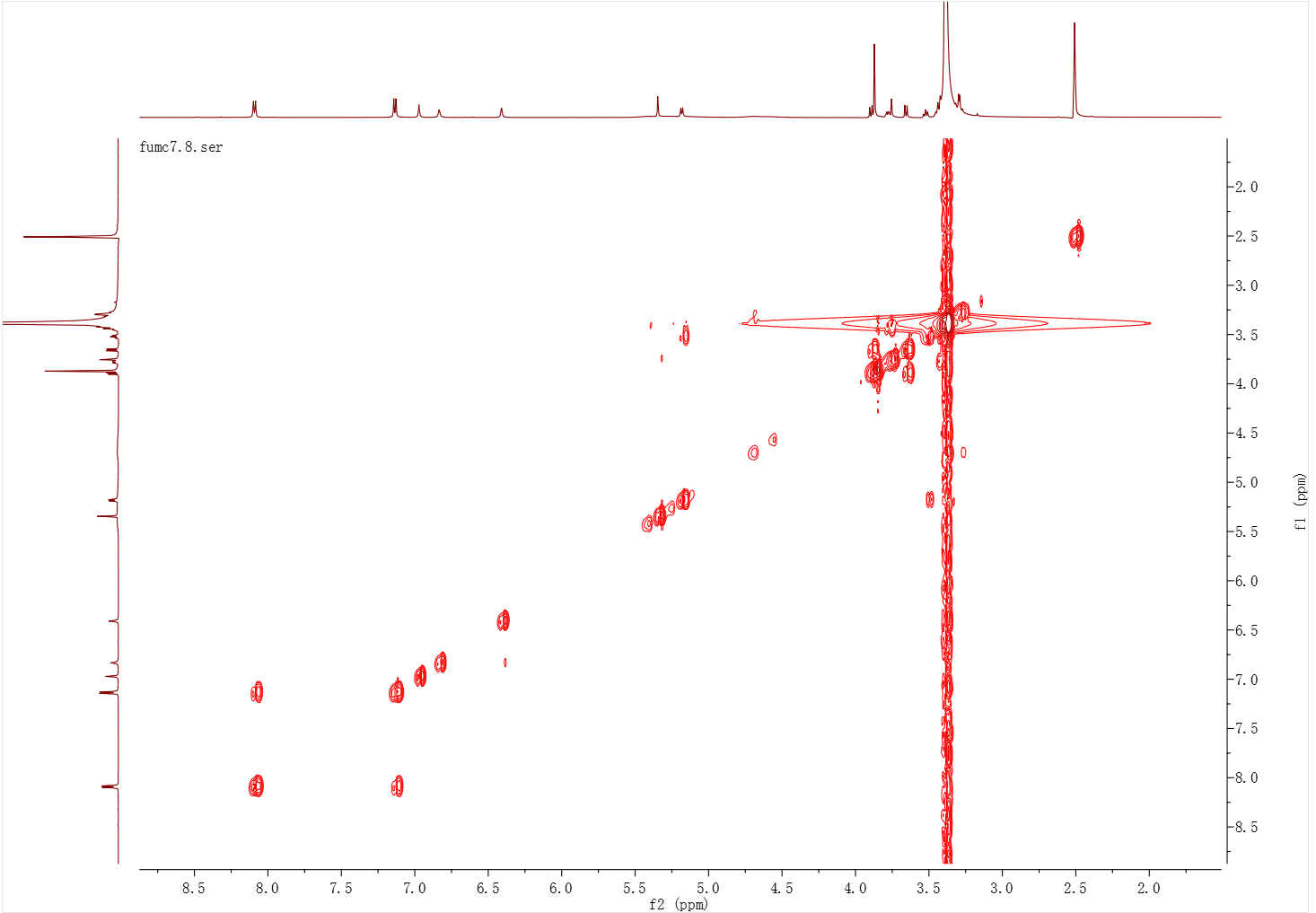


[Figure 5 ^1^H-^1^H COSY spectrum of compound **1**](#_Toc44082035)


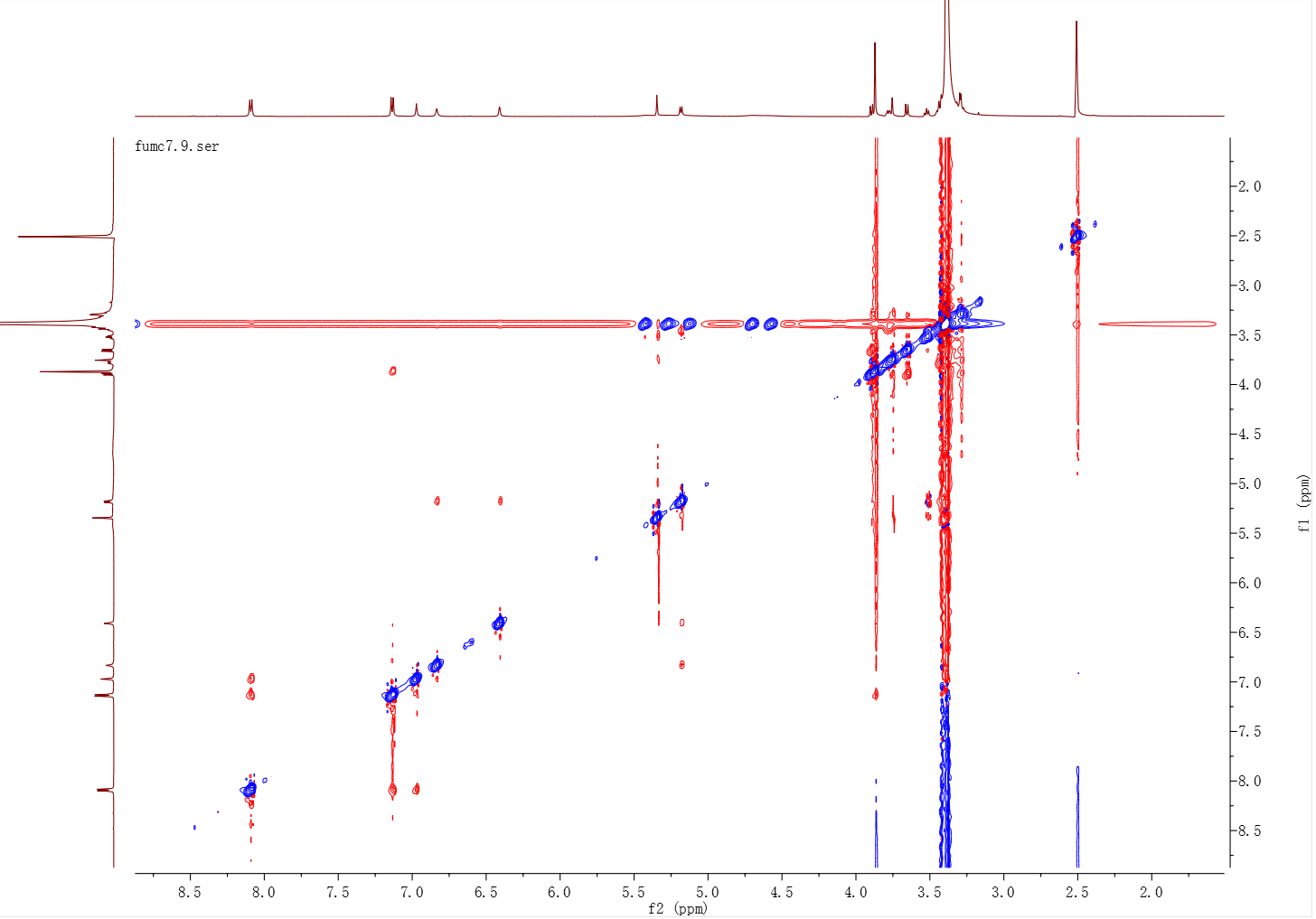


[Figure 6 ROSEY spectrum of compound **1**](#_Toc44082035)

Figure 7 HRESIMS spectrum of compound **1**


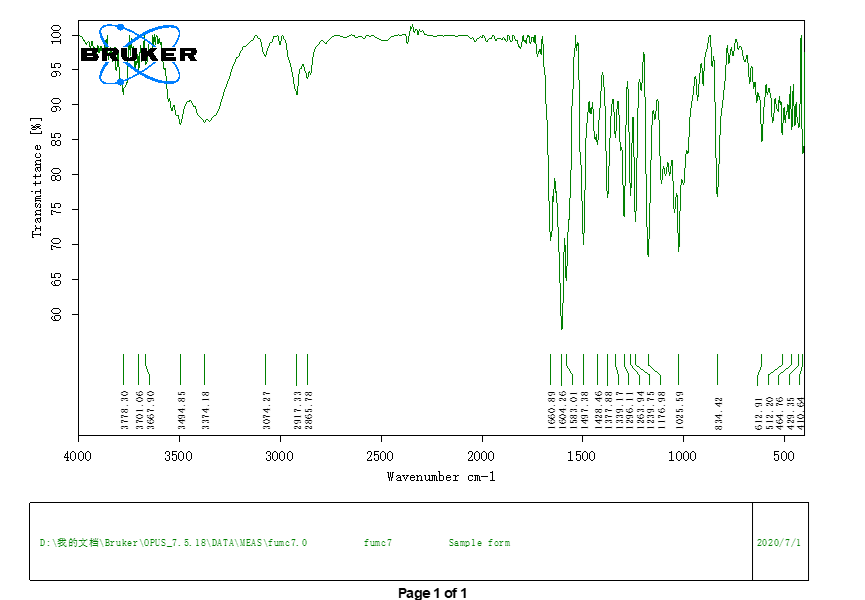


Figure 8 IR spectrum of compound **1**


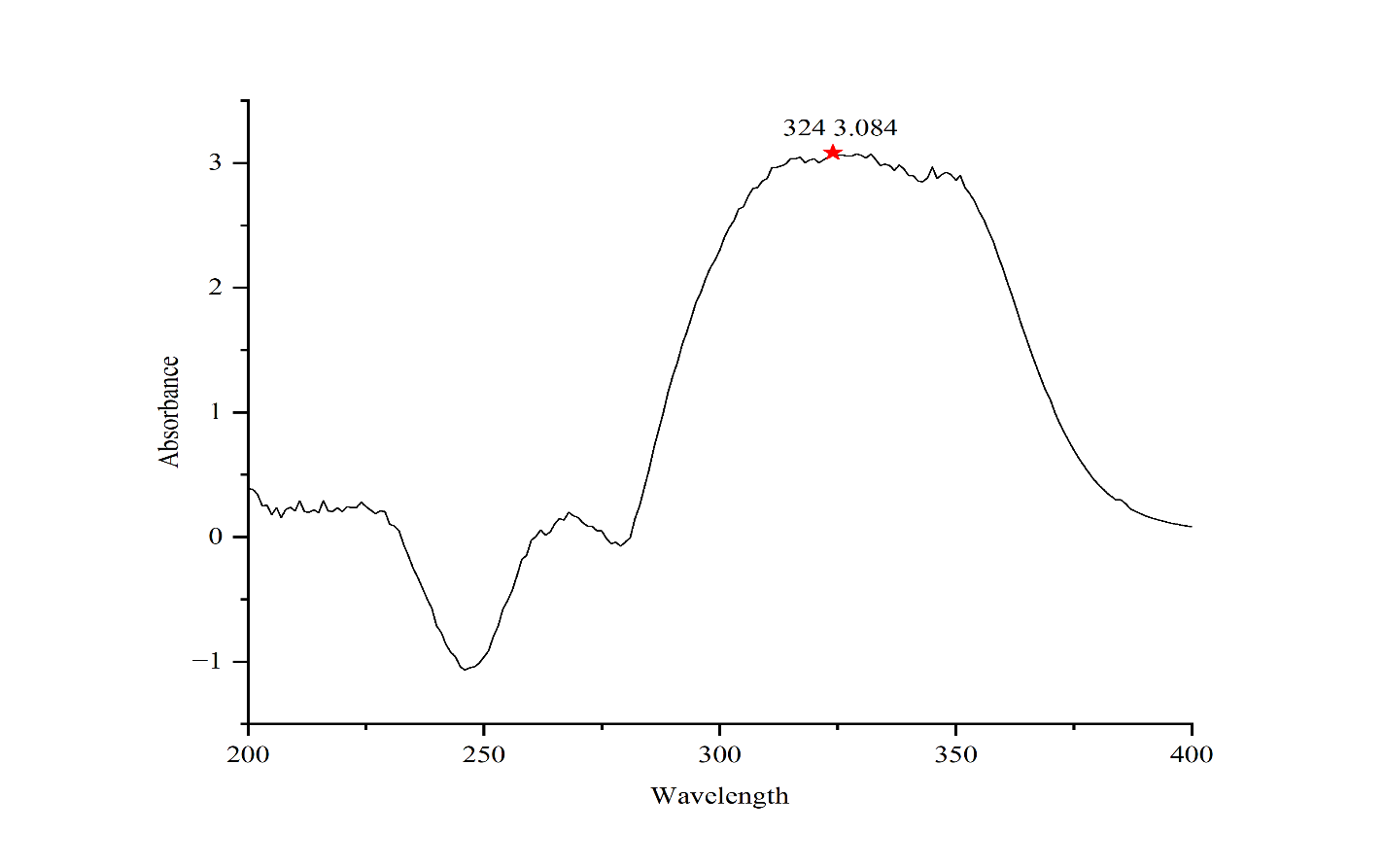


Figure 9 UV spectrum of compound **1**

Figure 10 ^1^H NMR spectrum of compound **2** (600 MHz, DMSO-*d*_6_)


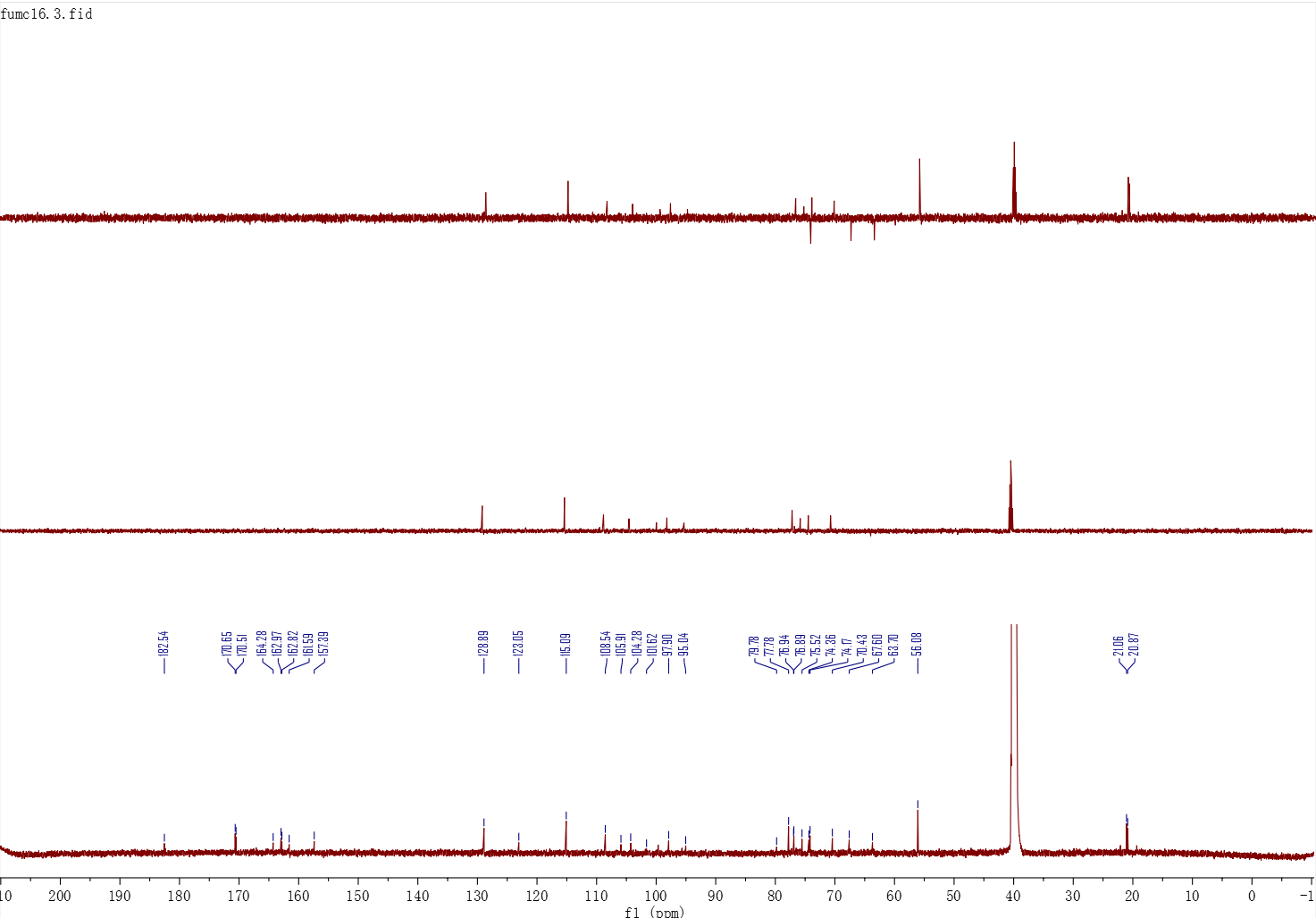


Figure 11 ^13^C NMR spectrum of compound **2** (150 MHz, DMSO-*d*_6_)


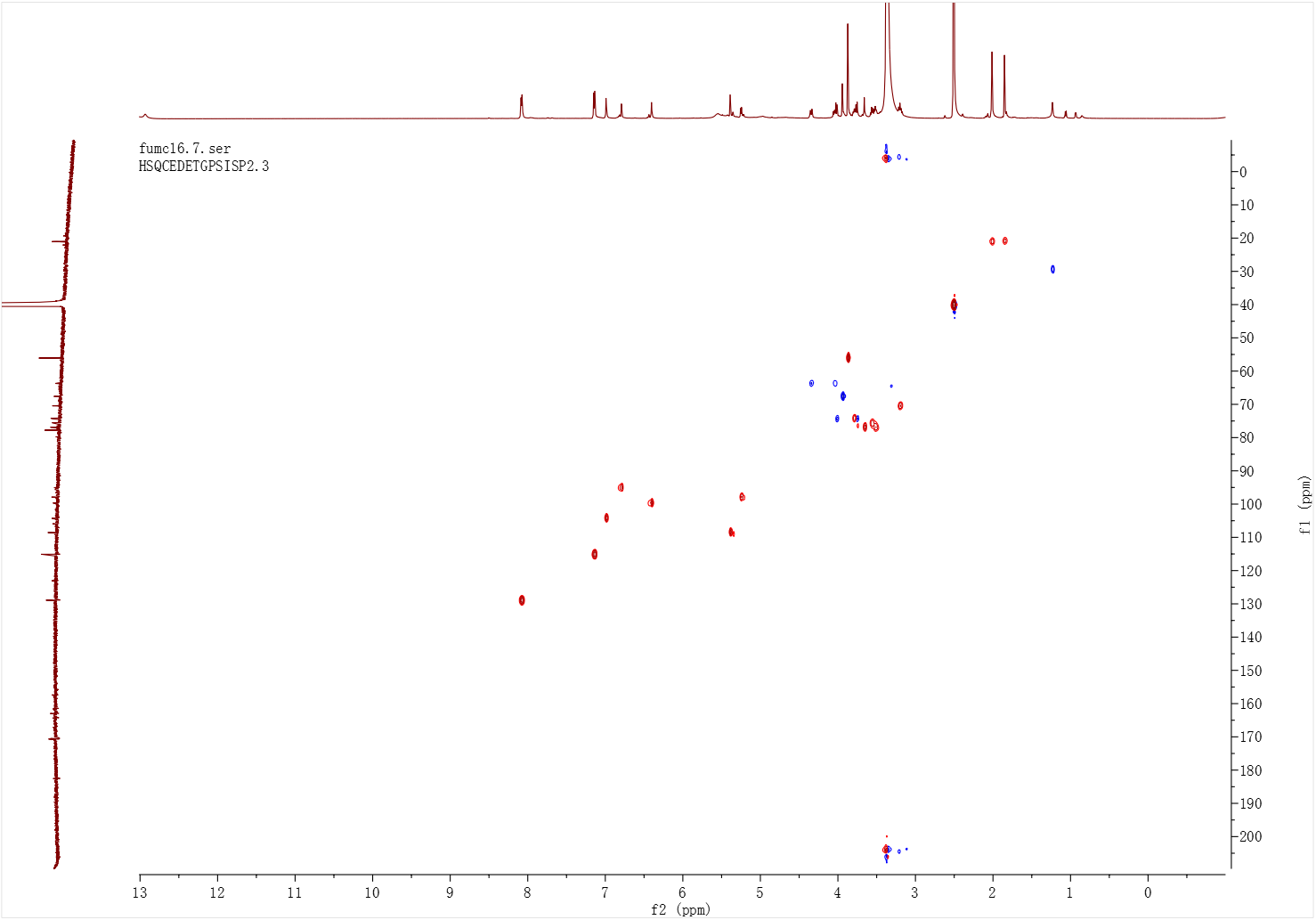


Figure 12 HSQC spectrum of compound **2**


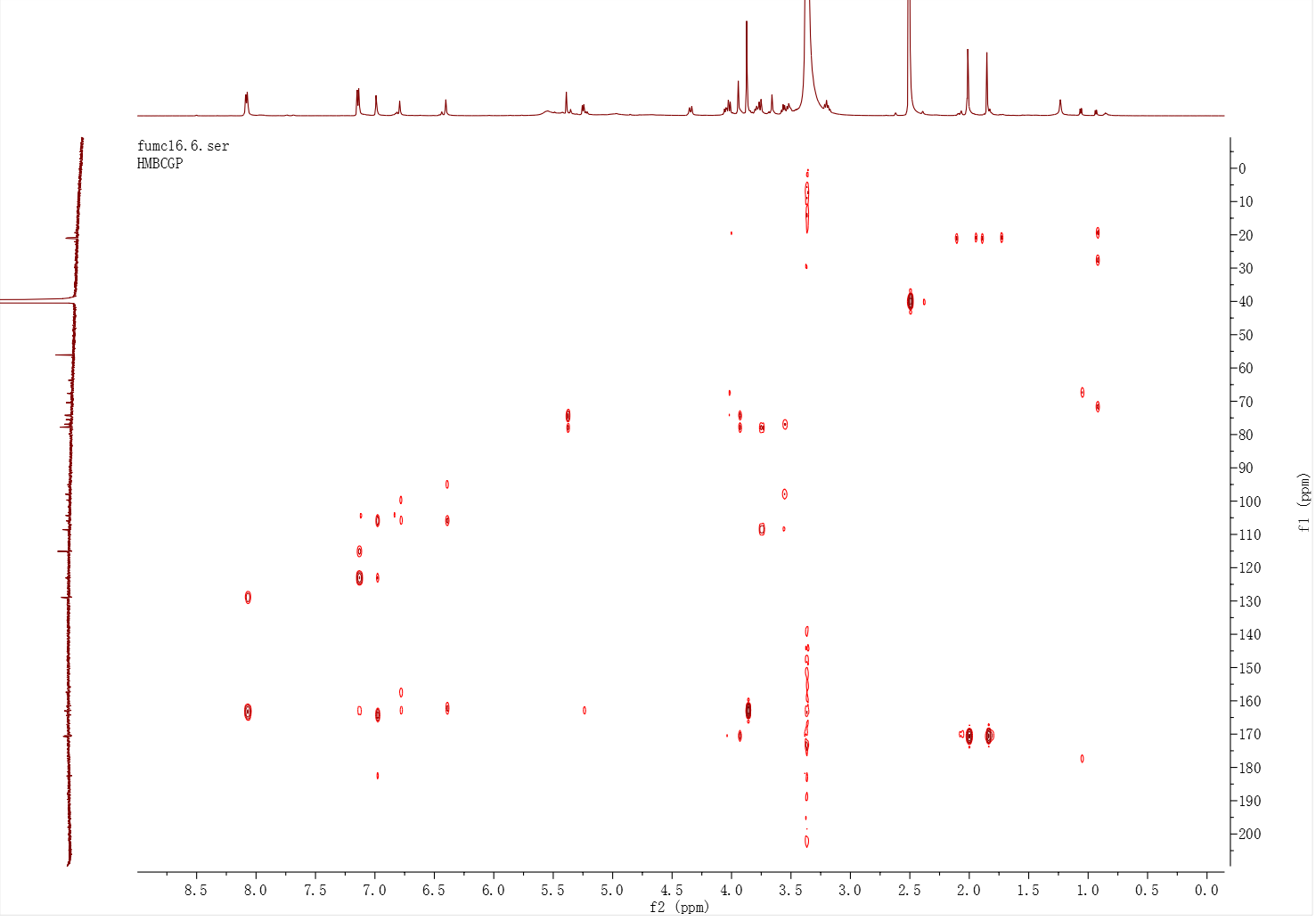


Figure 13 HMBC spectrum of compound **2**


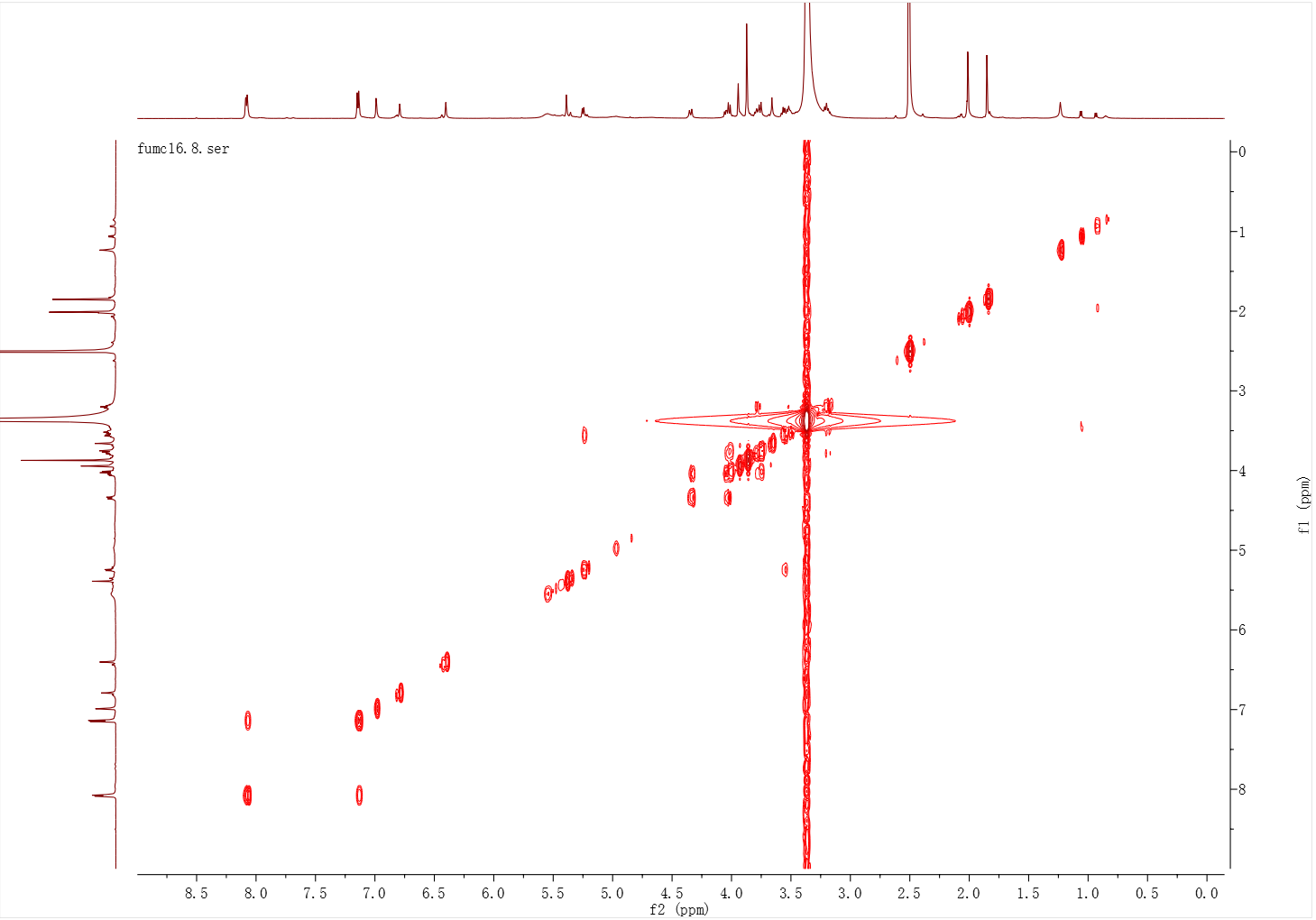


Figure 14 ^1^H–^1^H COSY spectrum of compound **2**

Figure 15 ROSEY spectrum of compound **2**

Figure 16 HRESIMS spectrum of compound **2**


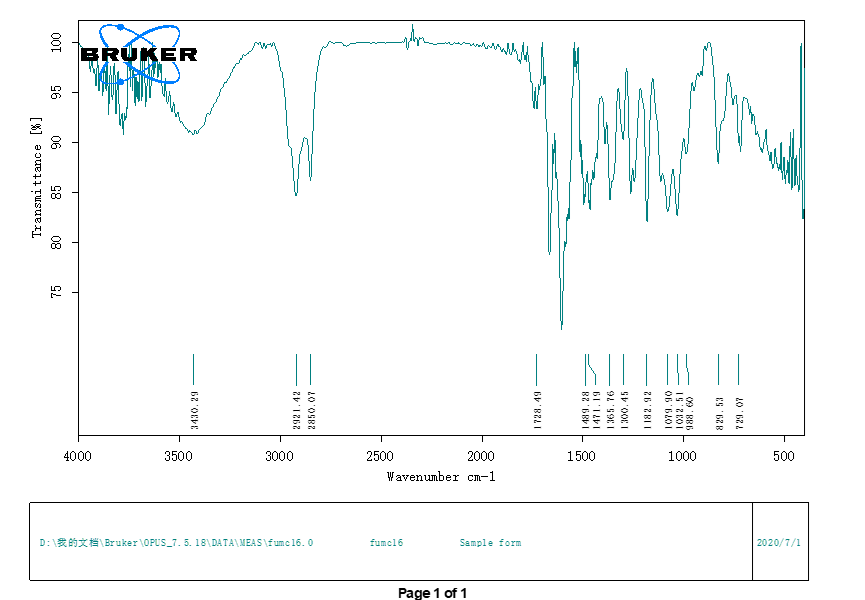


Figure 17 IR spectrum of compound **2**


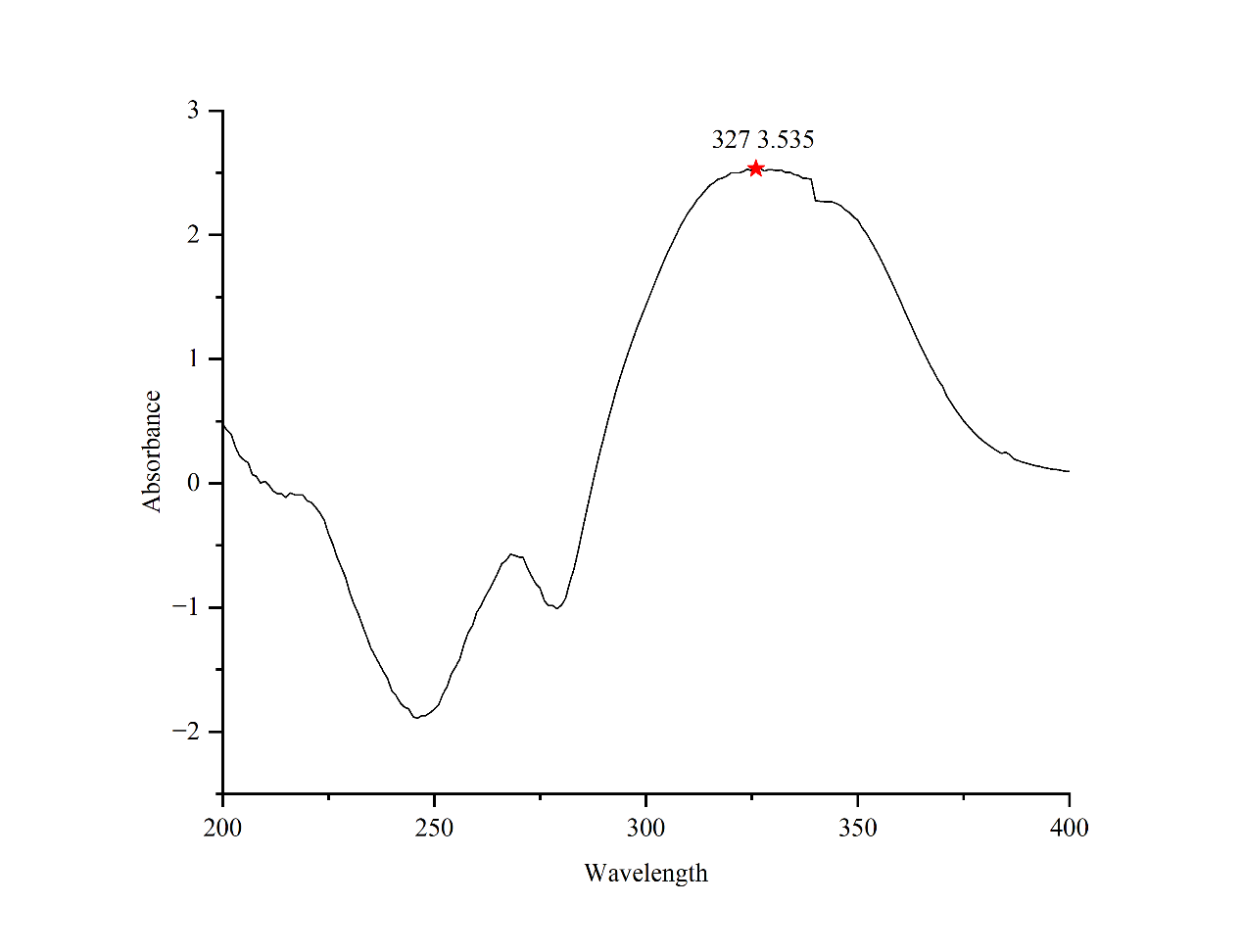


Figure 18 UV spectrum of compound **2**

Figure 19 ^1^H NMR spectrum of compound **3** (600 MHz, DMSO-*d*_6_)


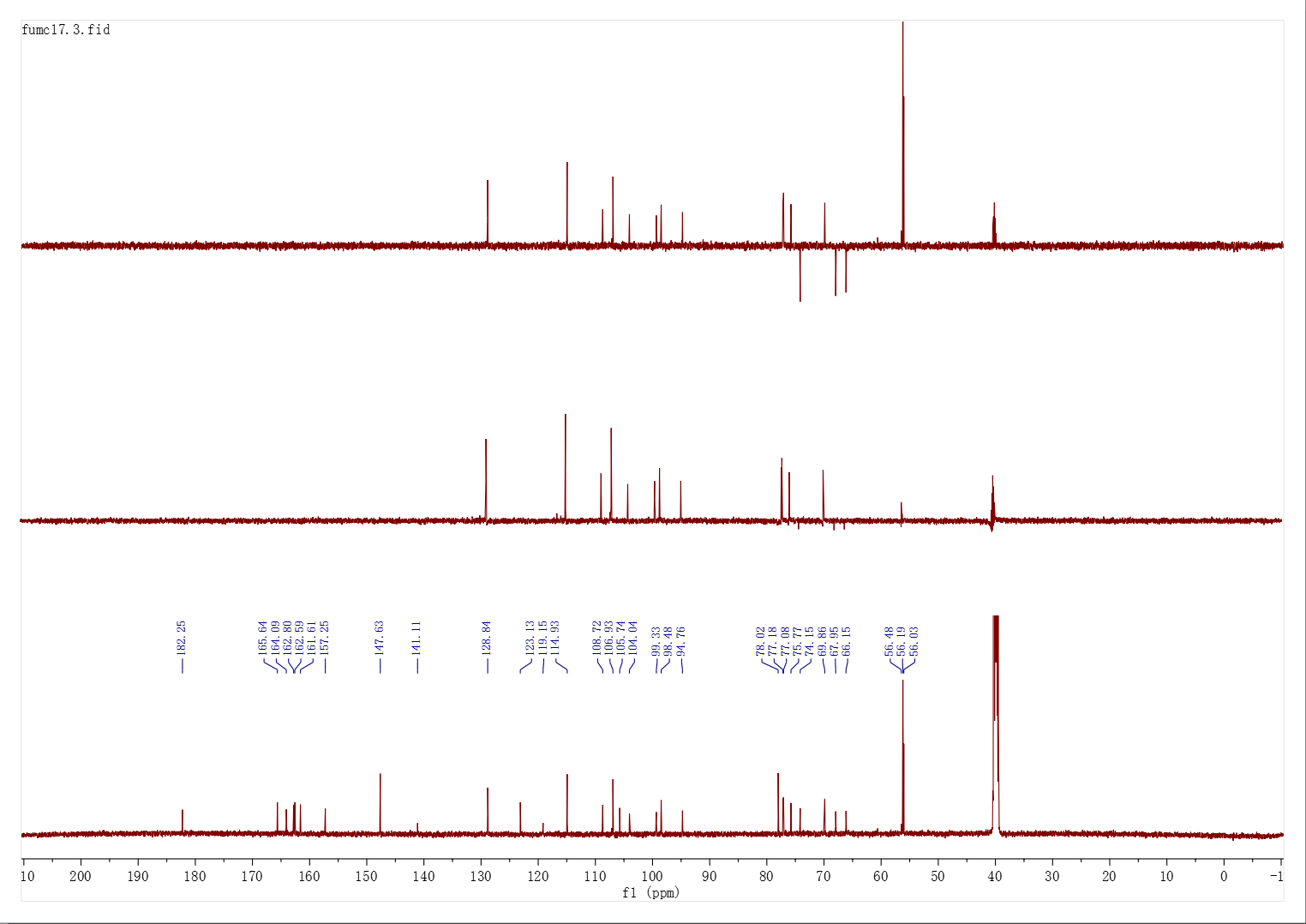


Figure 20 ^13^C NMR spectrum of compound **3** (150 MHz, DMSO-*d*_6_)

Figure 21 HSQC spectrum of compound **3**

Figure 22 HMBC spectrum of compound **3**

Figure 23 HRESIMS spectrum of compound **3**


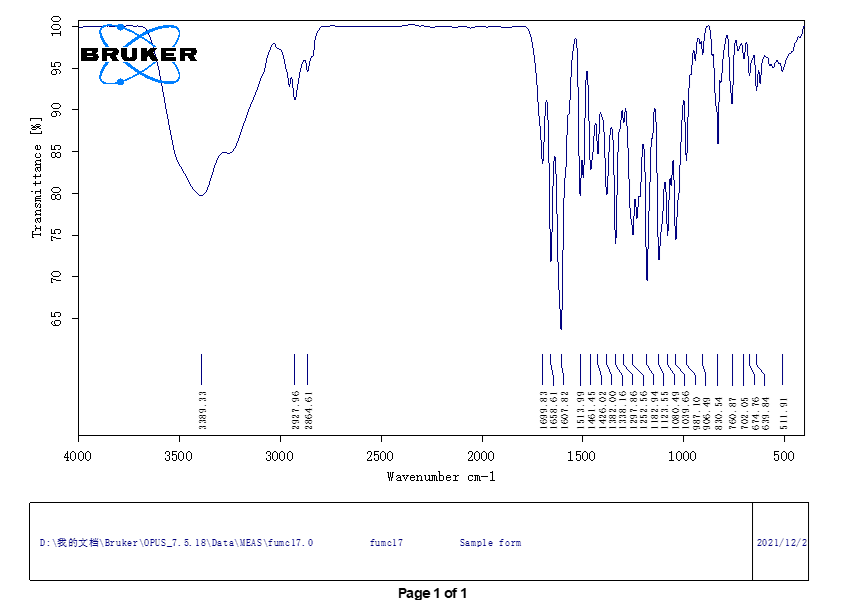


Figure 24 IR spectrum of compound **3**


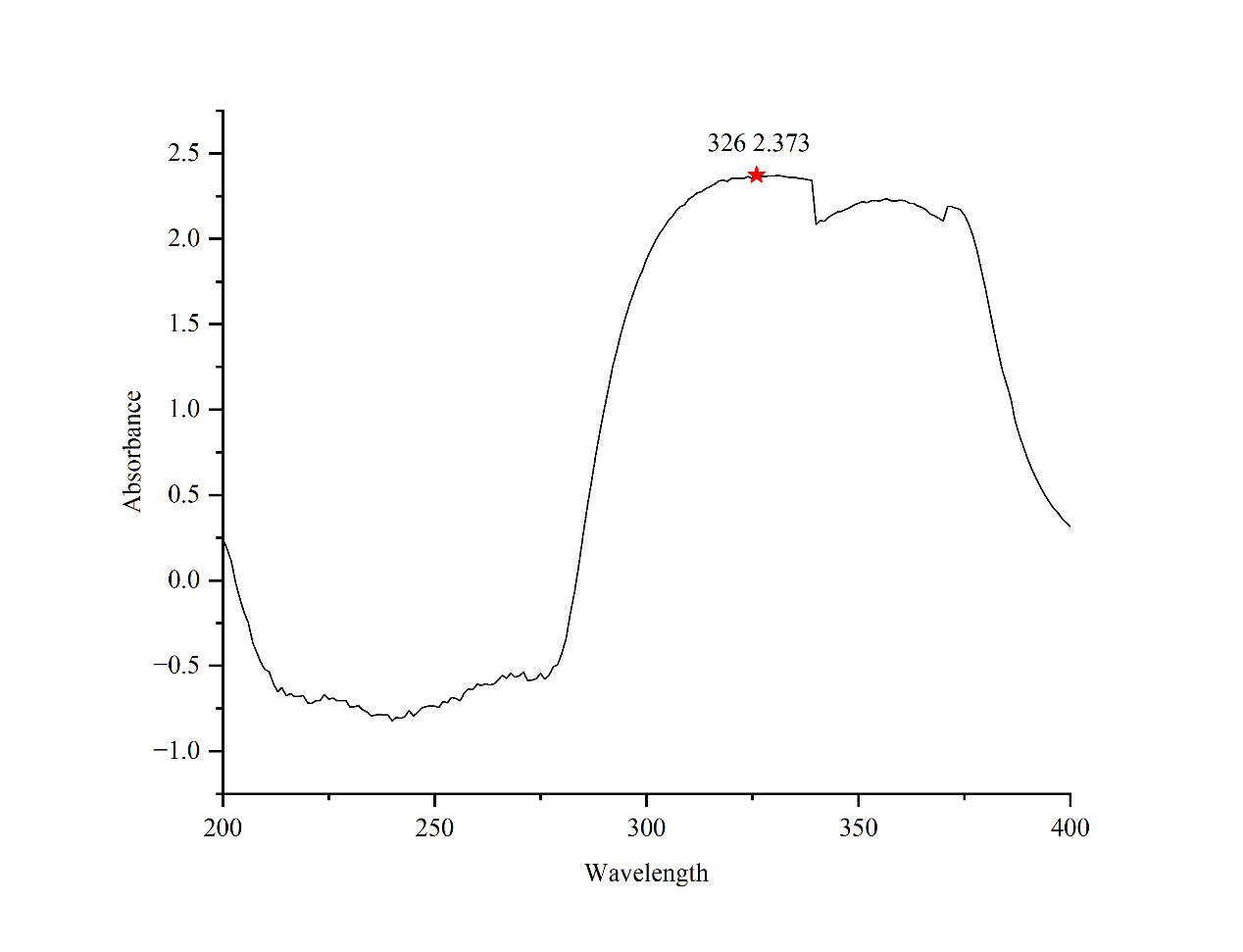


Figure 25 UV spectrum of compound **3**


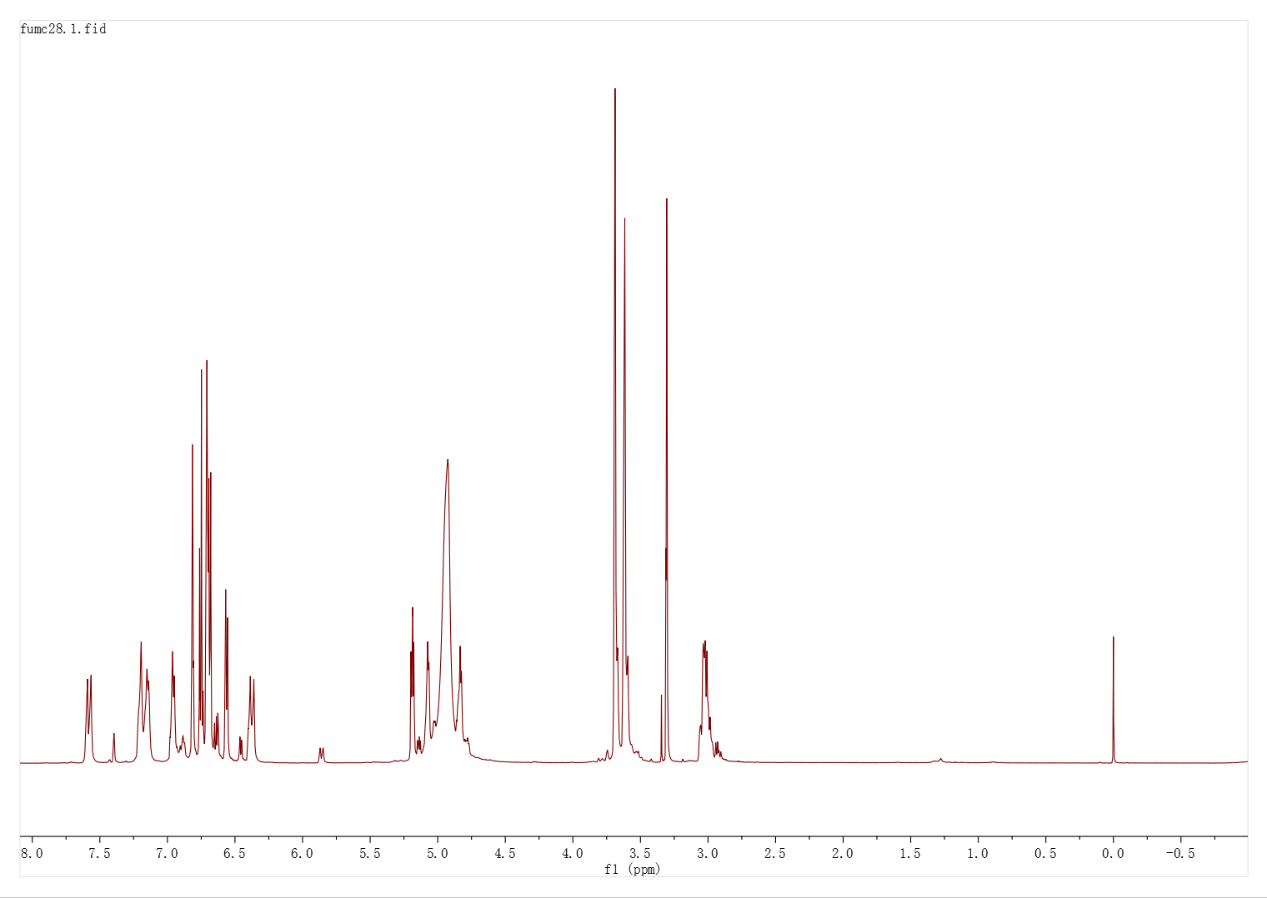


Figure 26 ^1^H NMR spectrum of compound **19** (600 MHz, CD_3_OD)


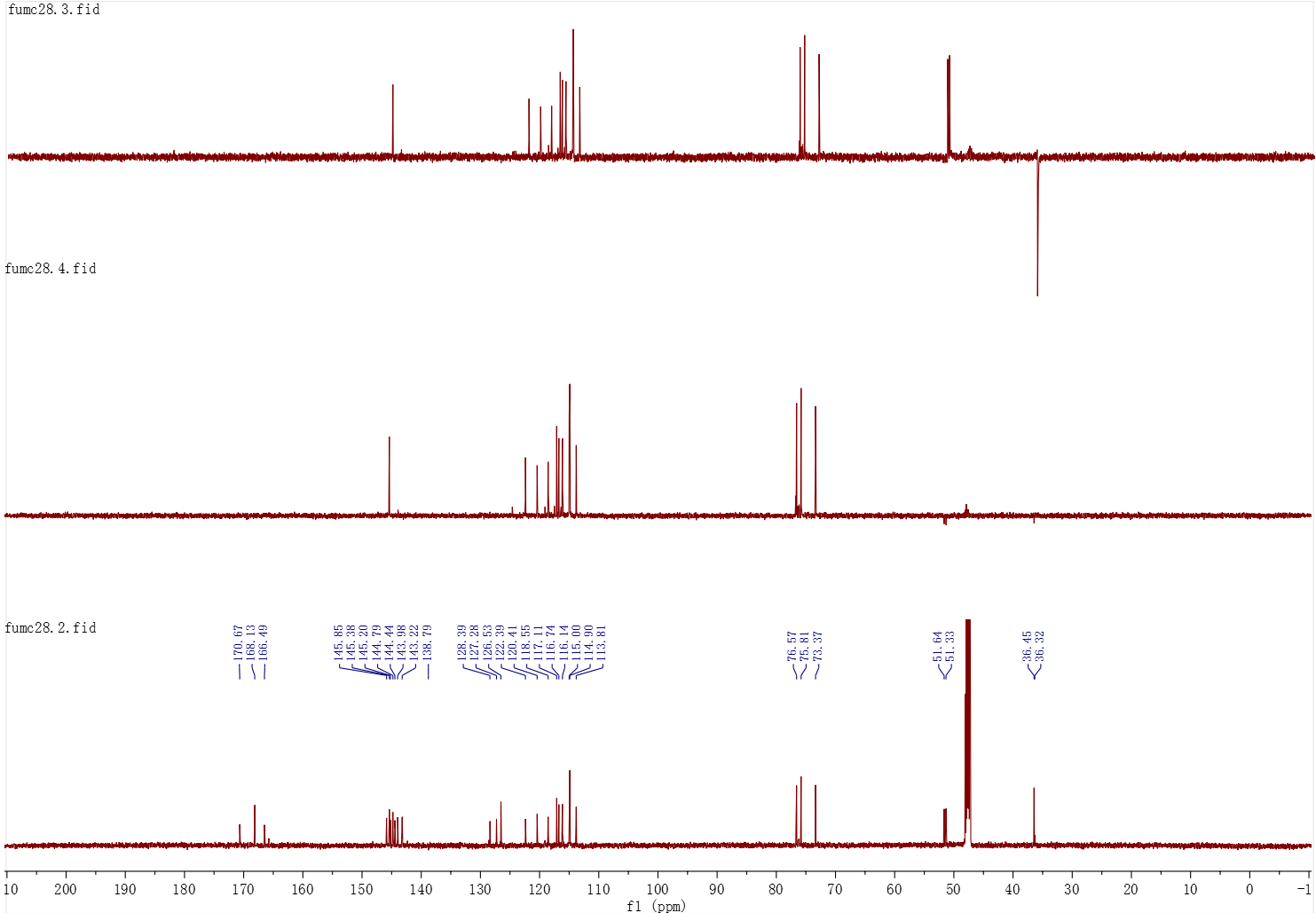


Figure 27 ^13^C NMR spectrum of compound **19** (150 MHz, CD_3_OD)


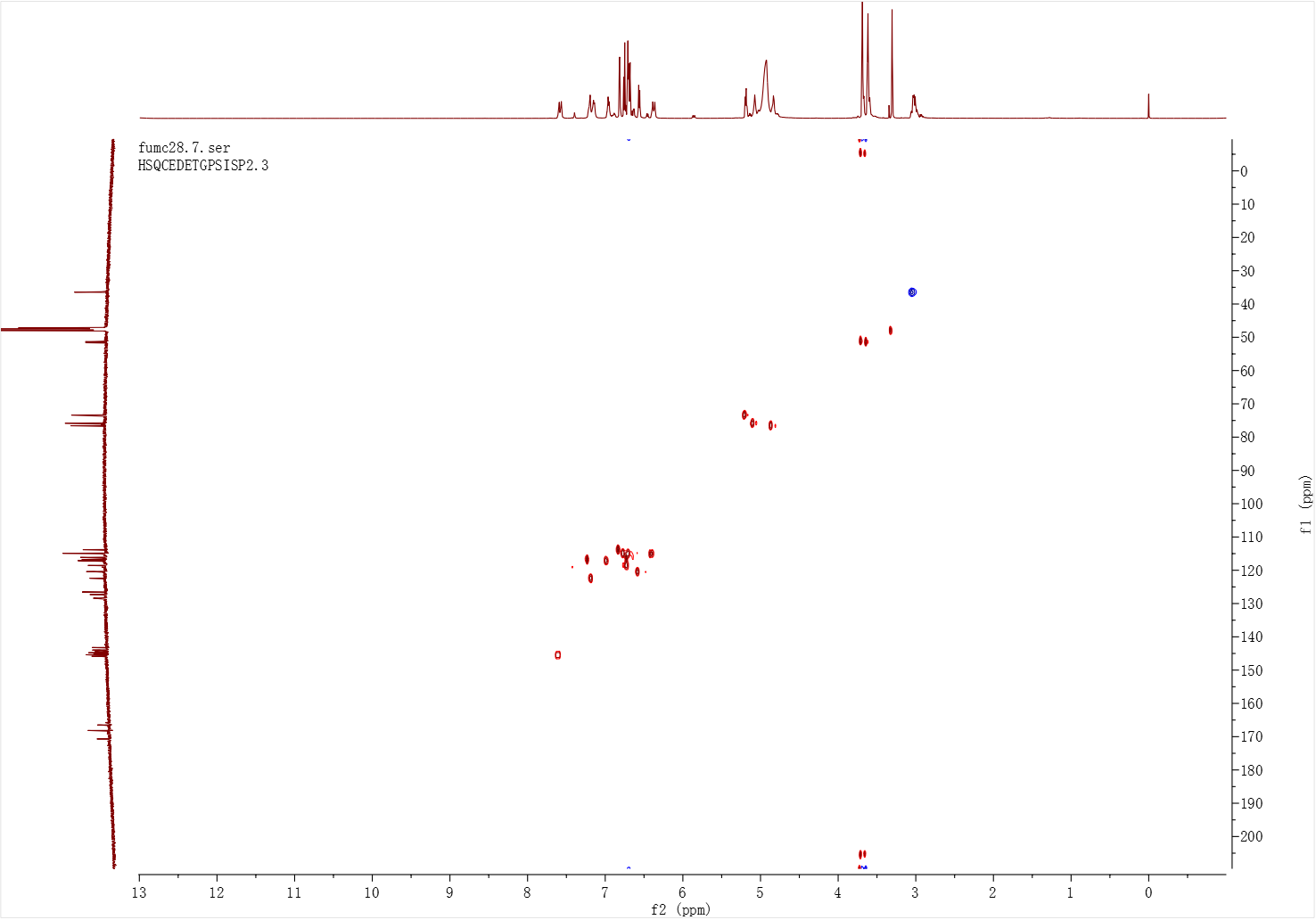


Figure 28 HSQC spectrum of compound **19**


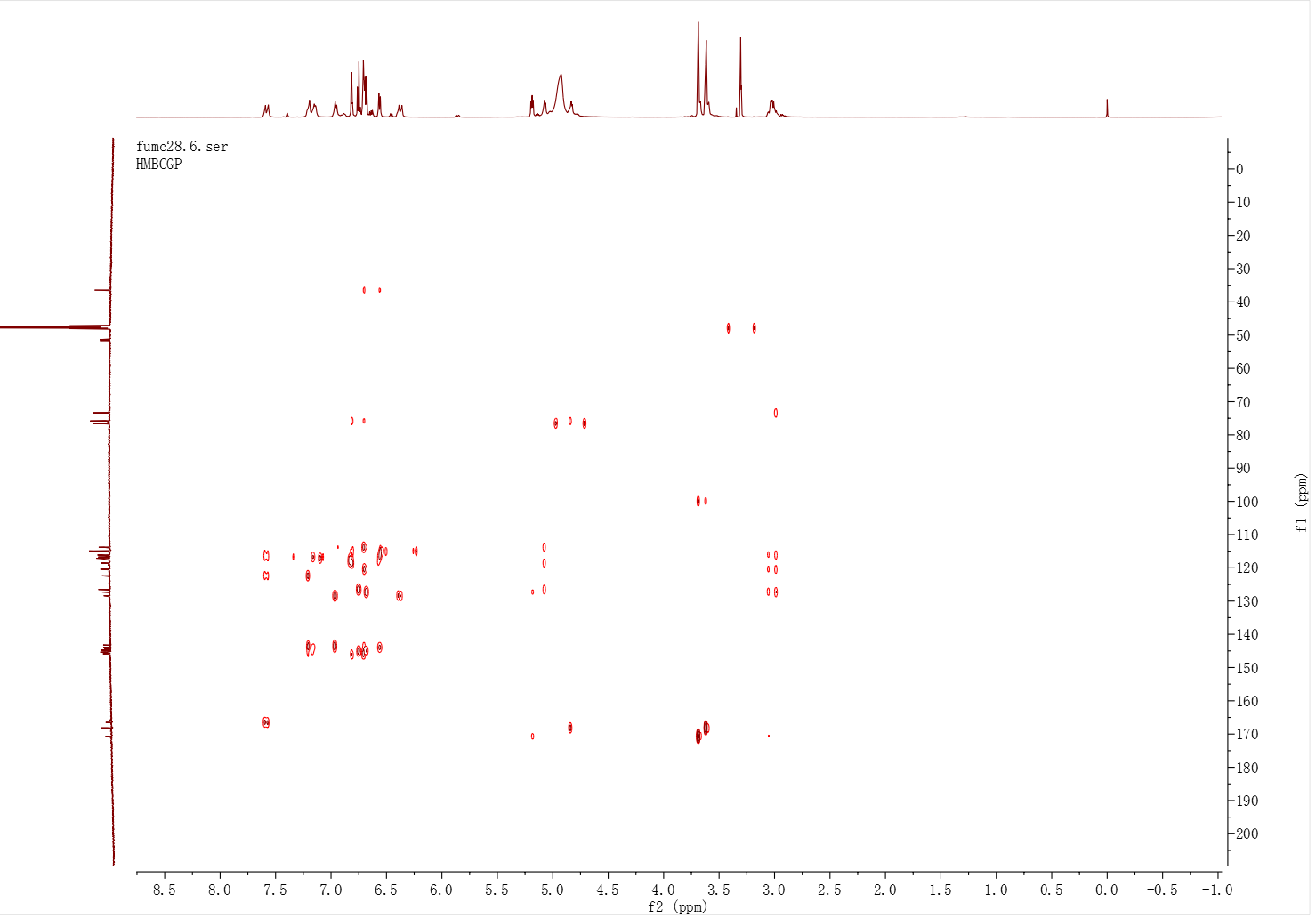


Figure 29 HMBC spectrum of compound **19**


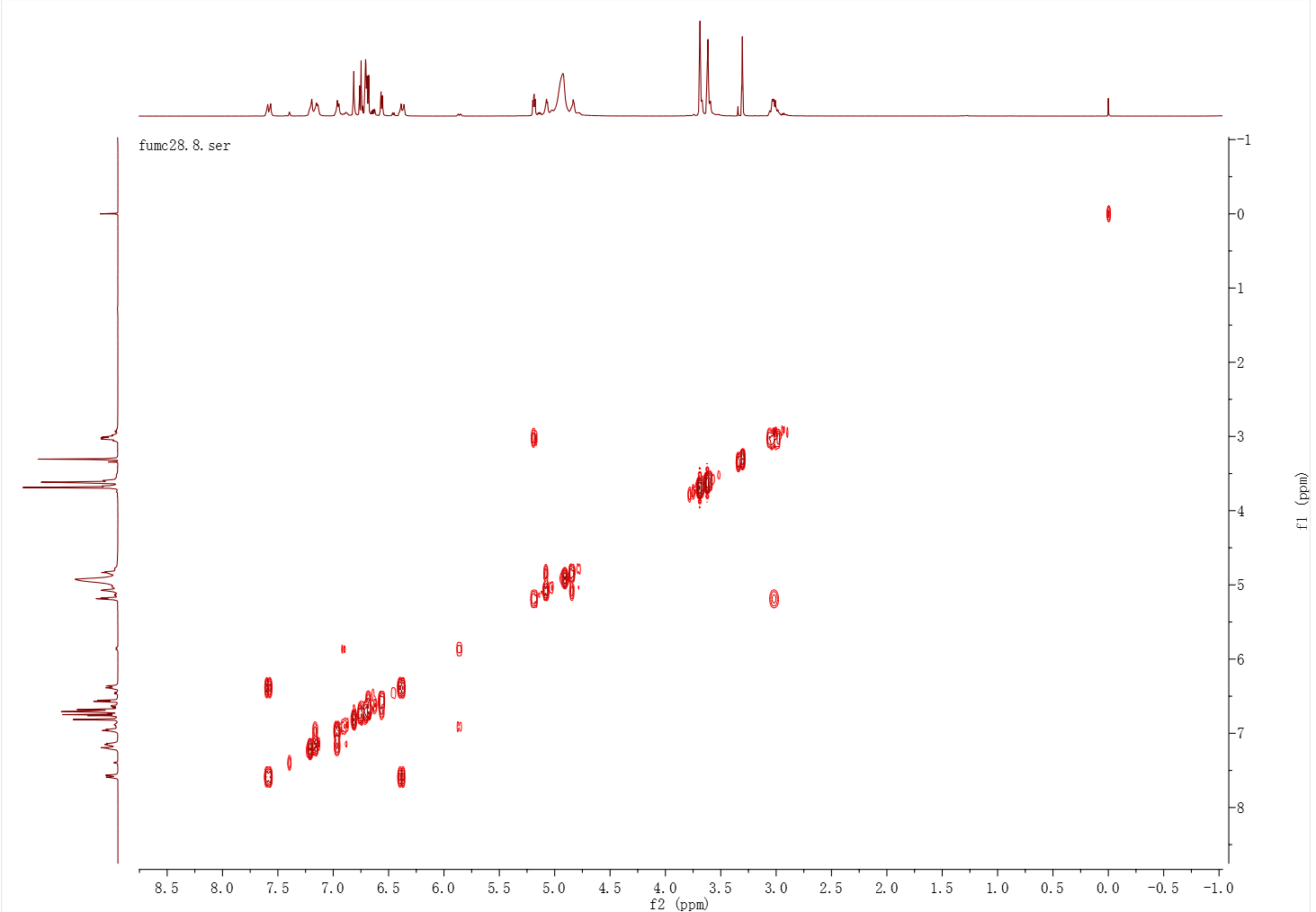


Figure 30 ^1^H-^1^H COSY spectrum of compound **19**


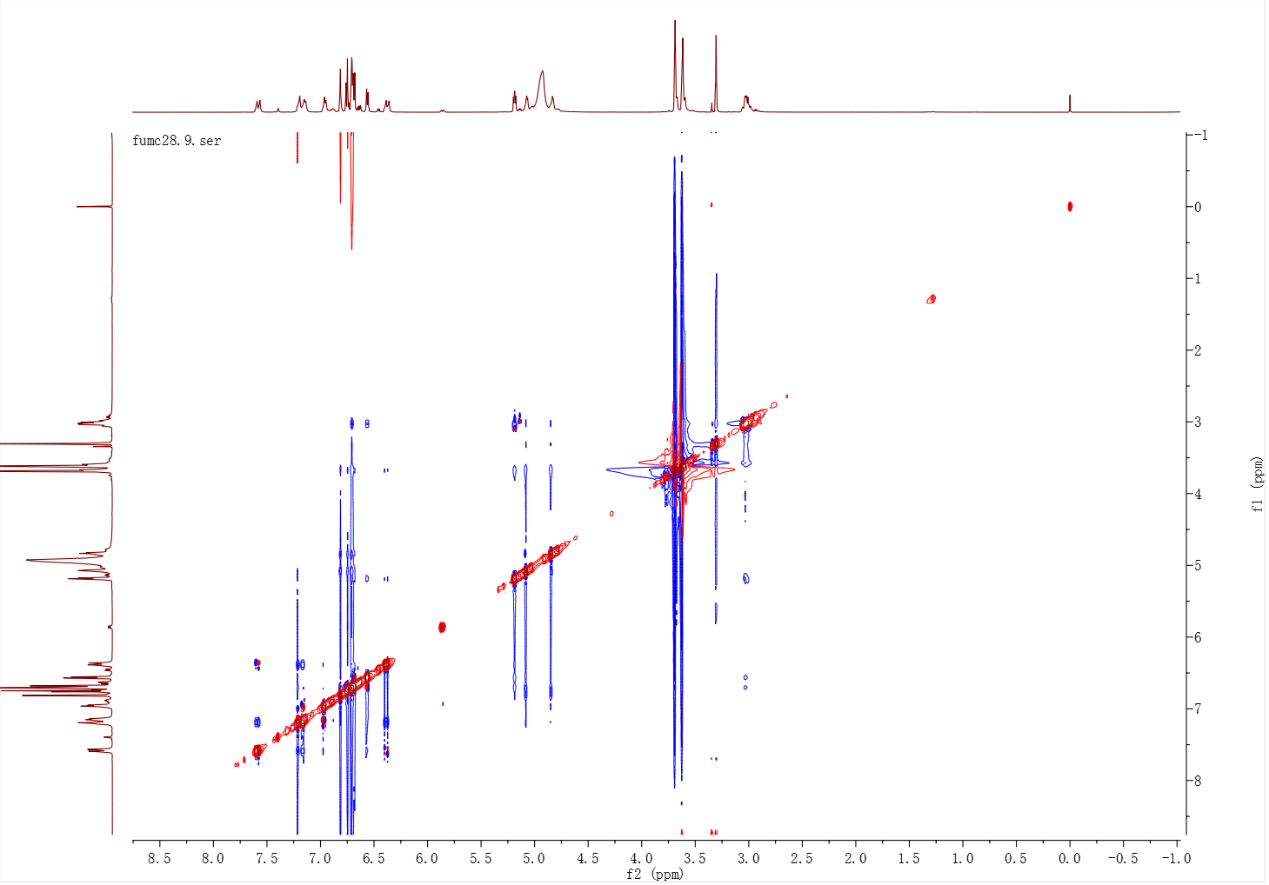


Figure 31 ROSEY spectrum of compound **19**


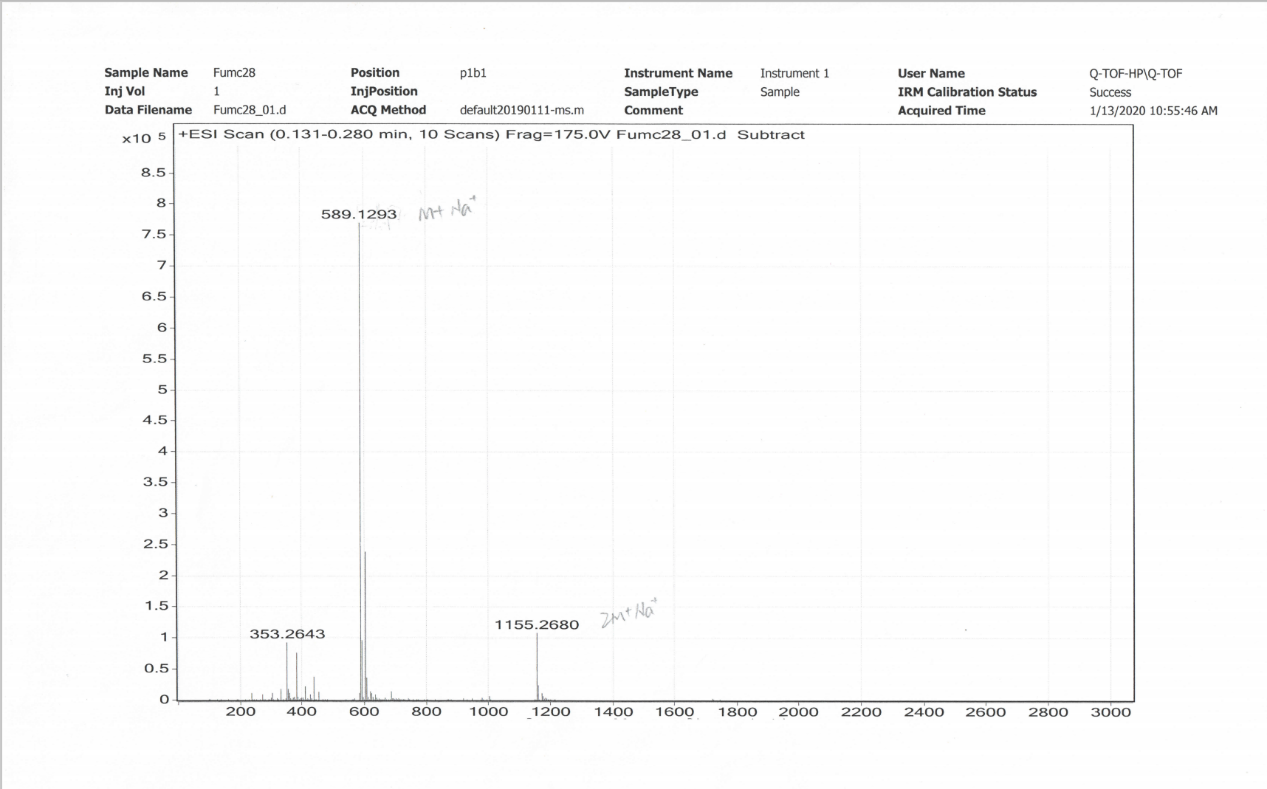


Figure 32 HRESIMS spectrum of compound **19**


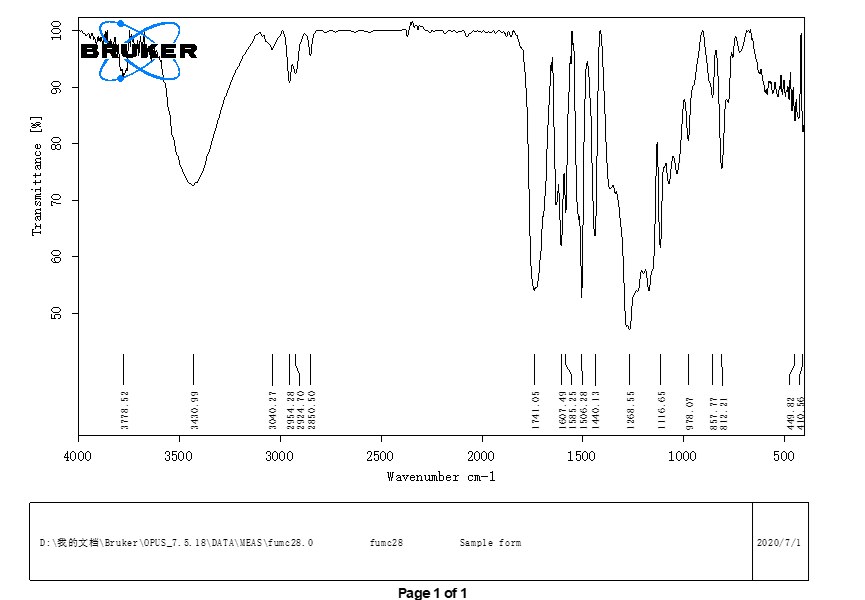


Figure 33 IR spectrum of compound **19**


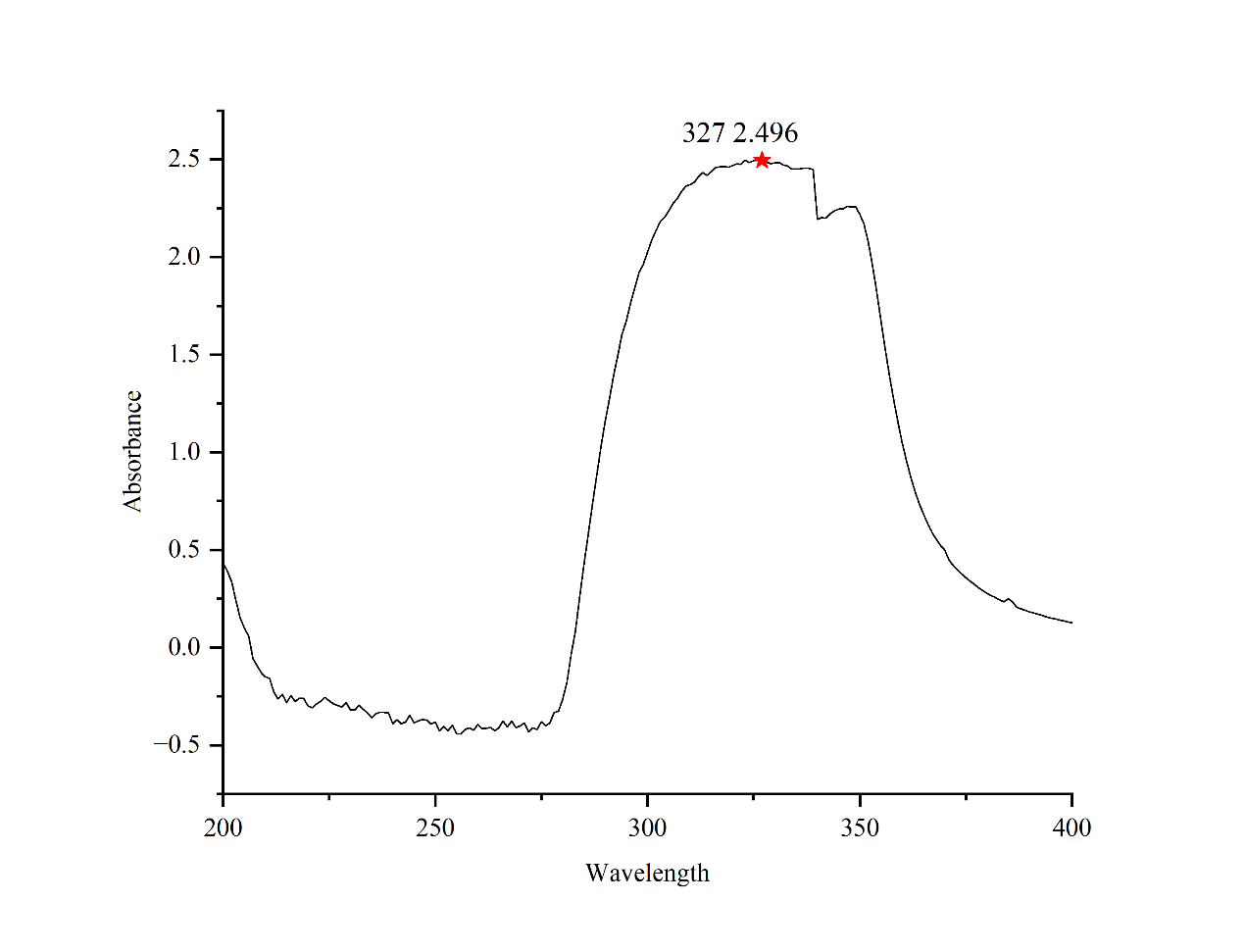


Figure 34 UV spectrum of compound **19**


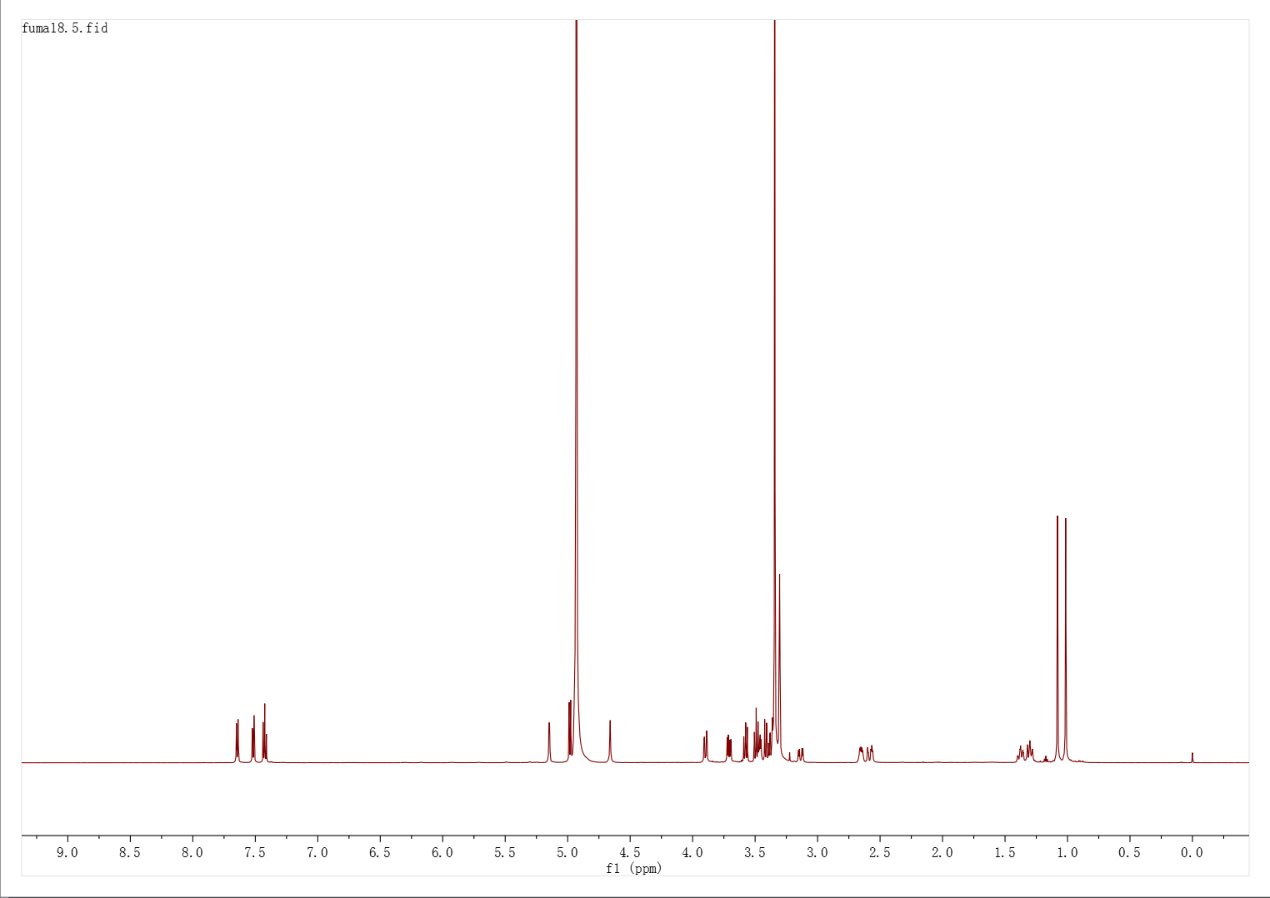


Figure 35 ^1^H NMR spectrum of compound **27** (600 MHz, CD_3_OD)


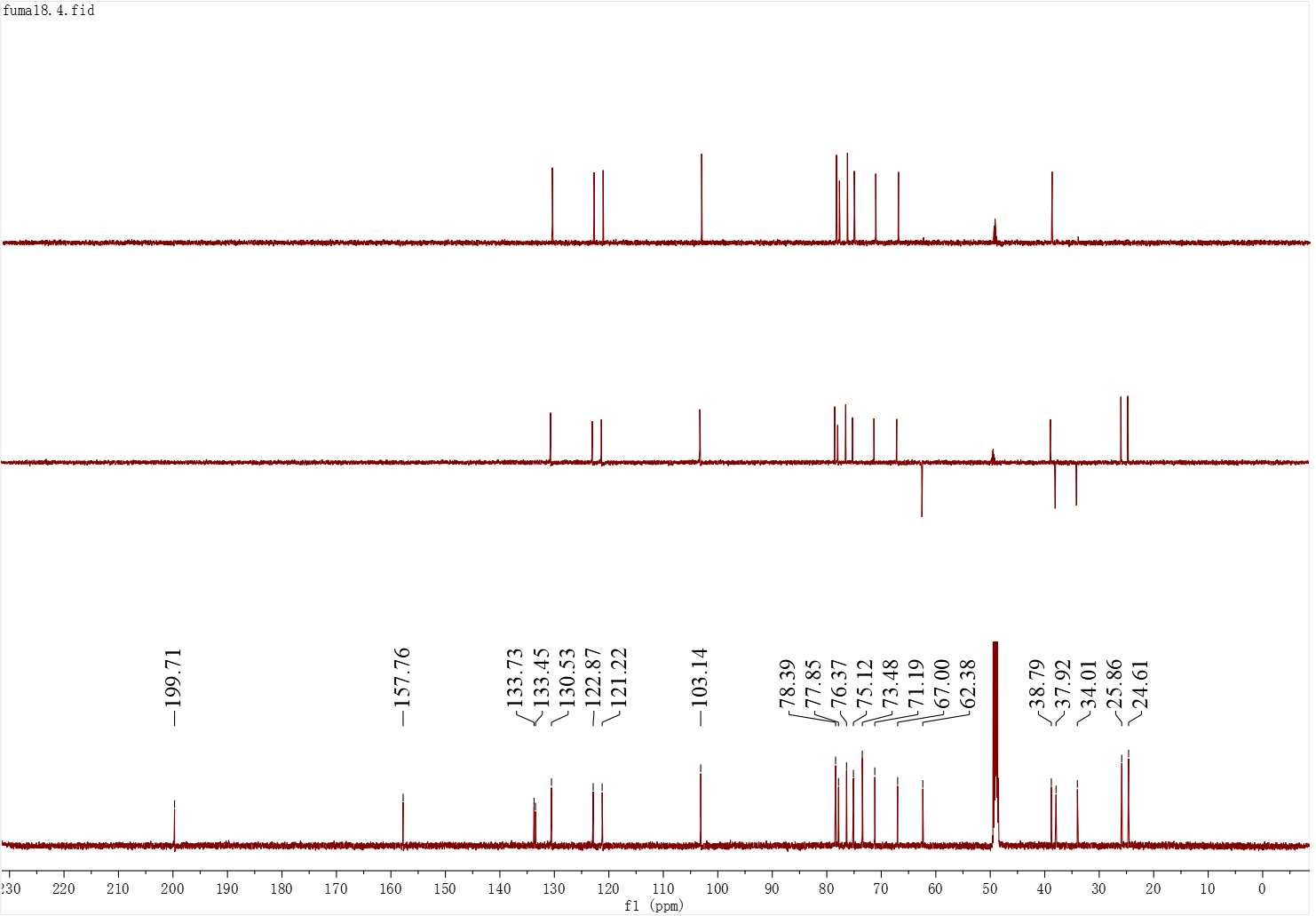


Figure 36 ^13^C NMR spectrum of compound **27** (150 MHz, CD_3_OD)


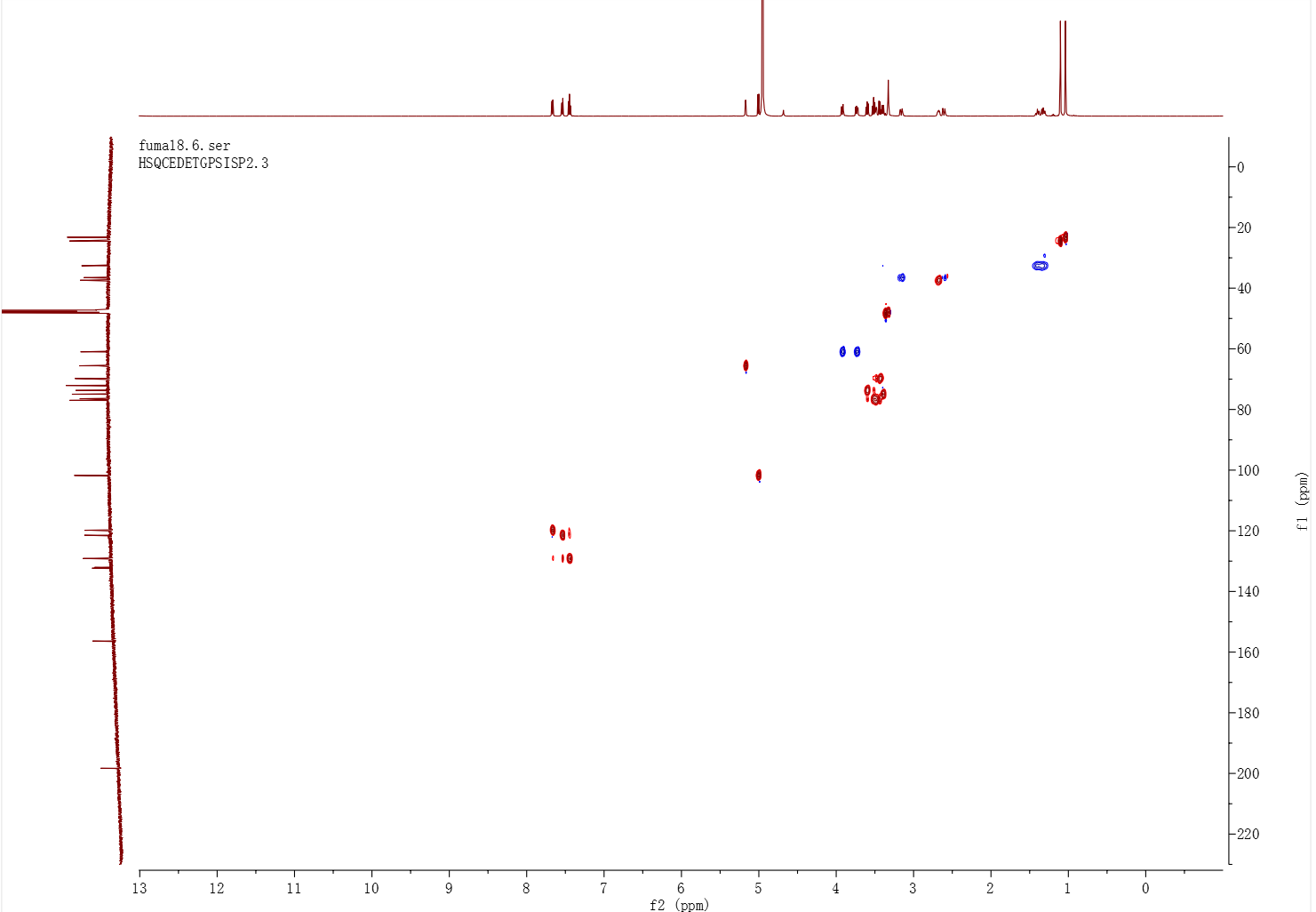


Figure 37 HSQC spectrum of compound **27**


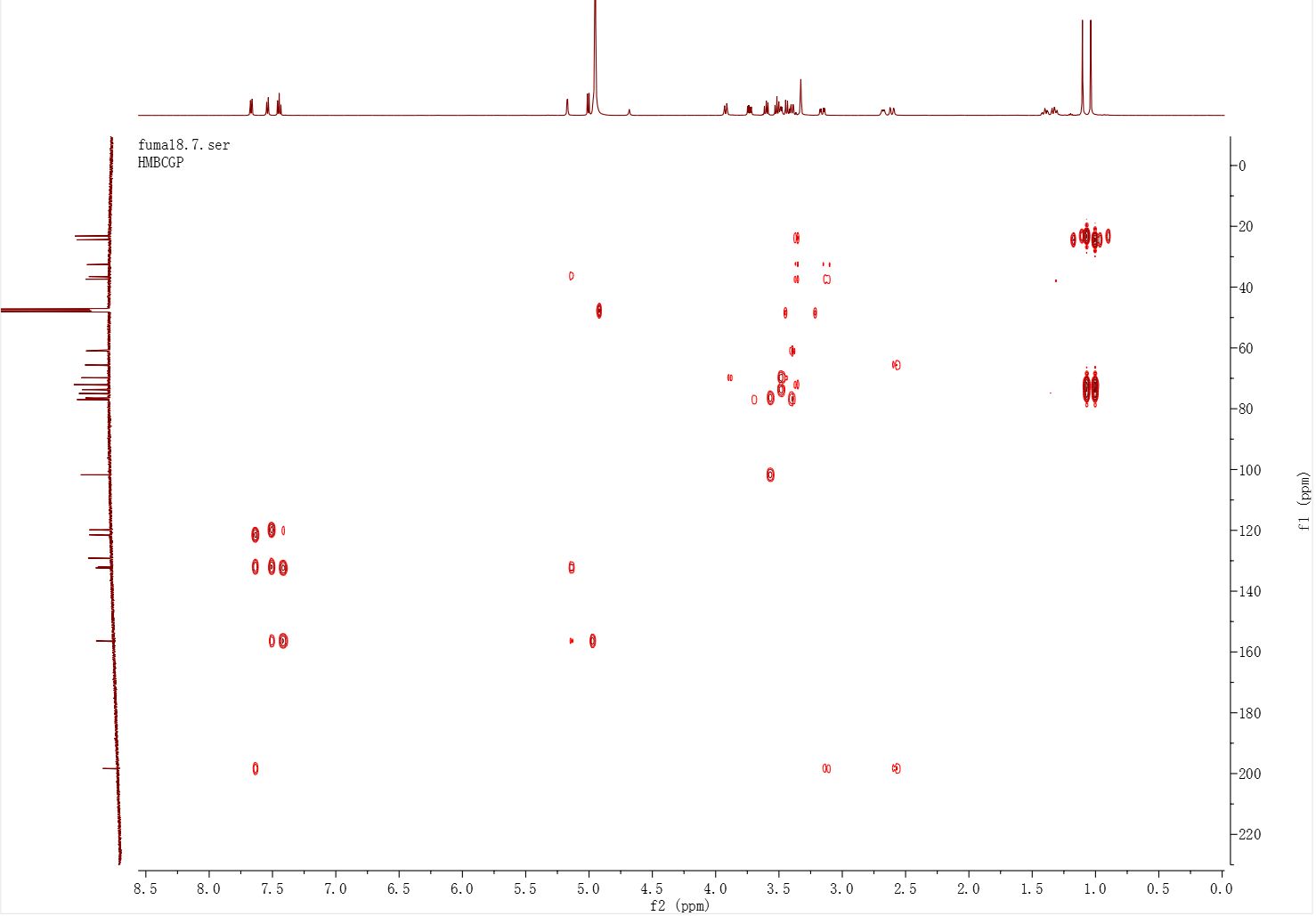


Figure 38 HMBC spectrum of compound **27**


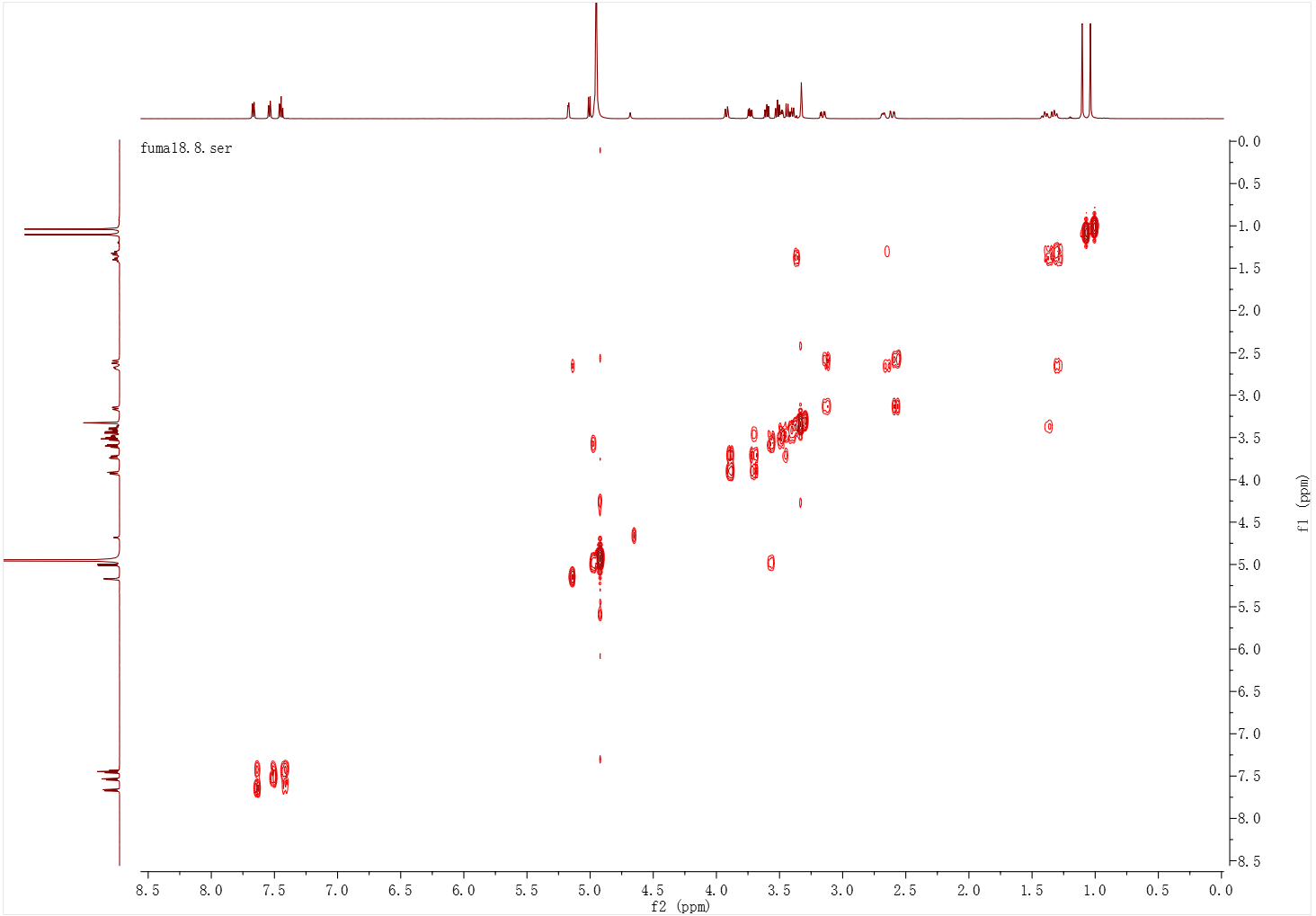


Figure 39 ^1^H-^1^H COSY spectrum of compound **27**


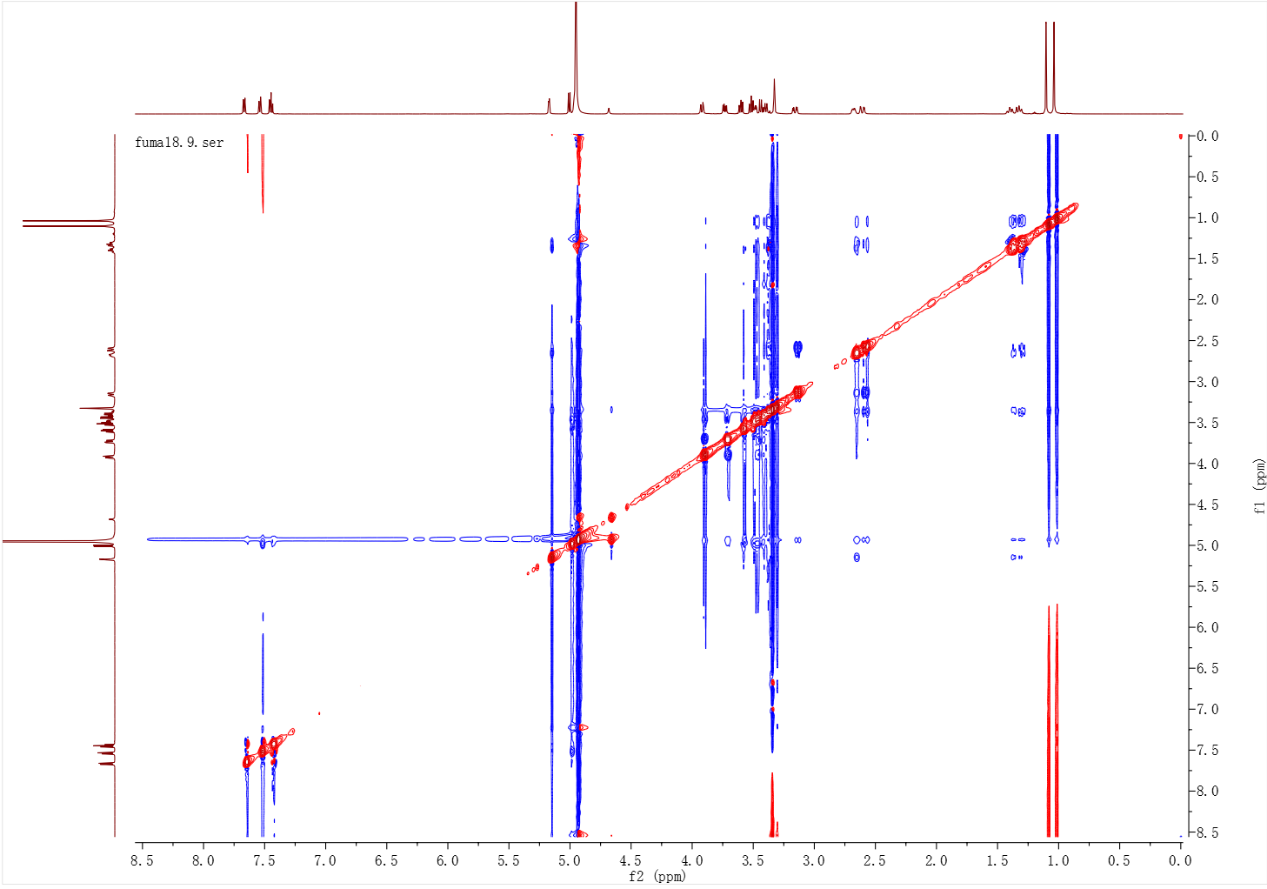


Figure 40 ROESY spectrum of compound **27**


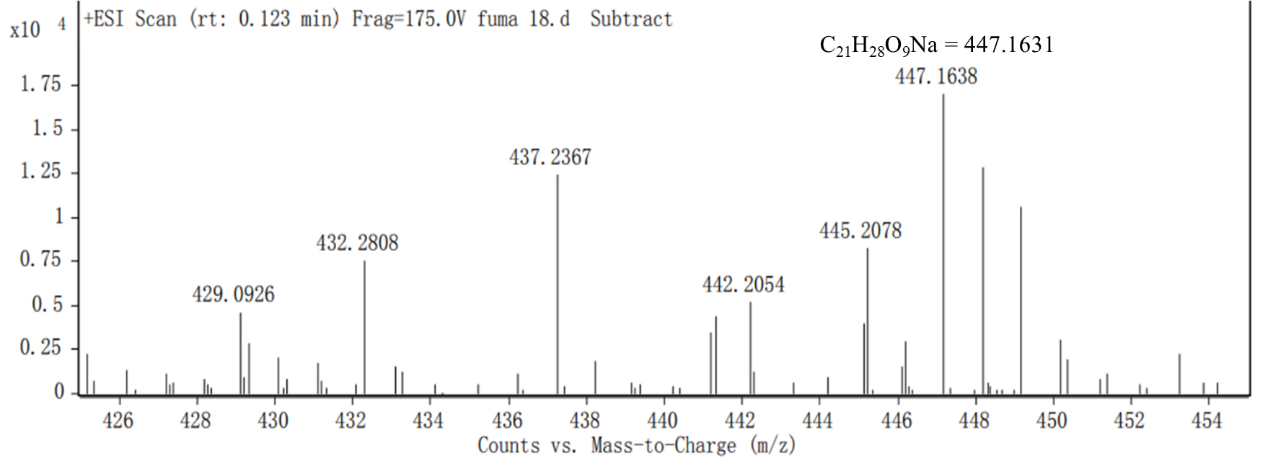


Figure 41 HRESIMS spectrum of compound 27


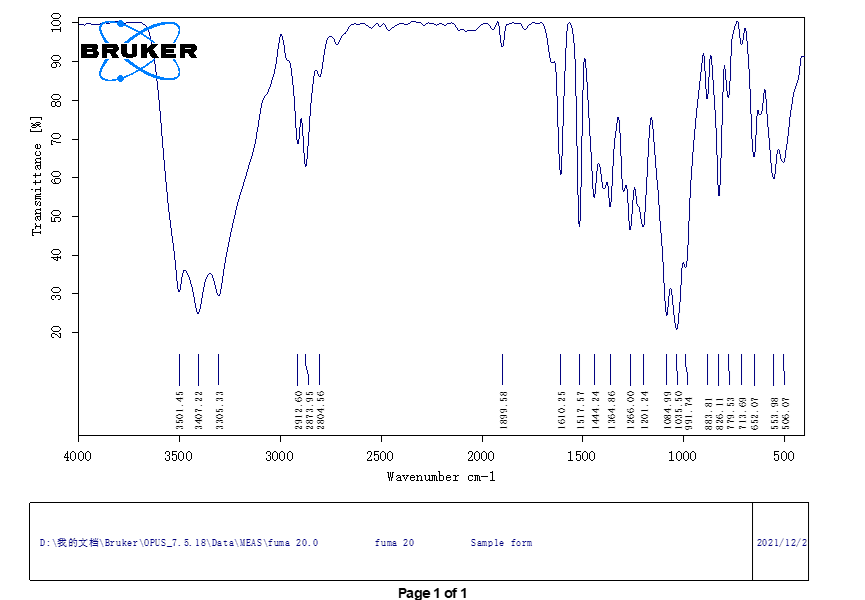


Figure 42 IR spectrum of compound **27**


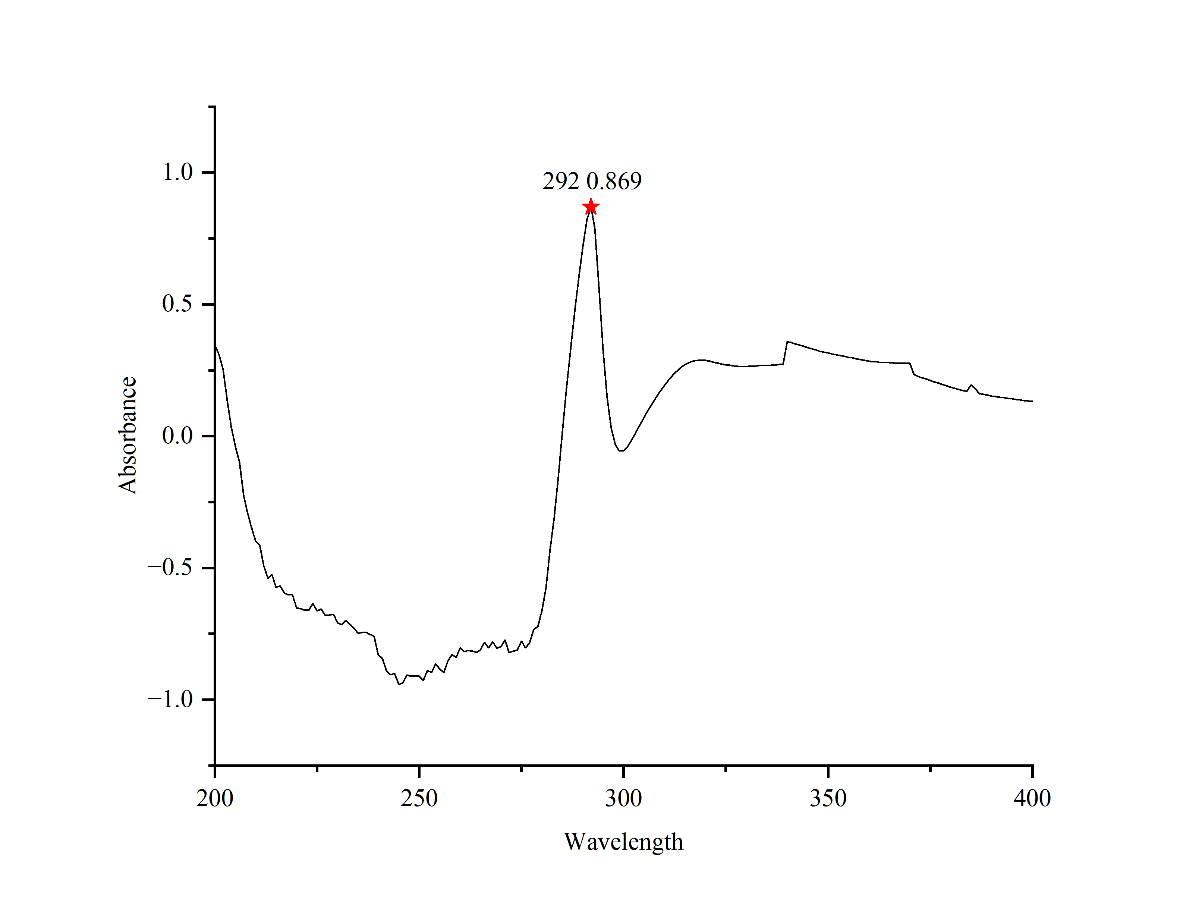


Figure 43 UV spectrum of compound **27**

Figure 44 ^1^H NMR spectrum of compound **28** (600 MHz, CD_3_OD)


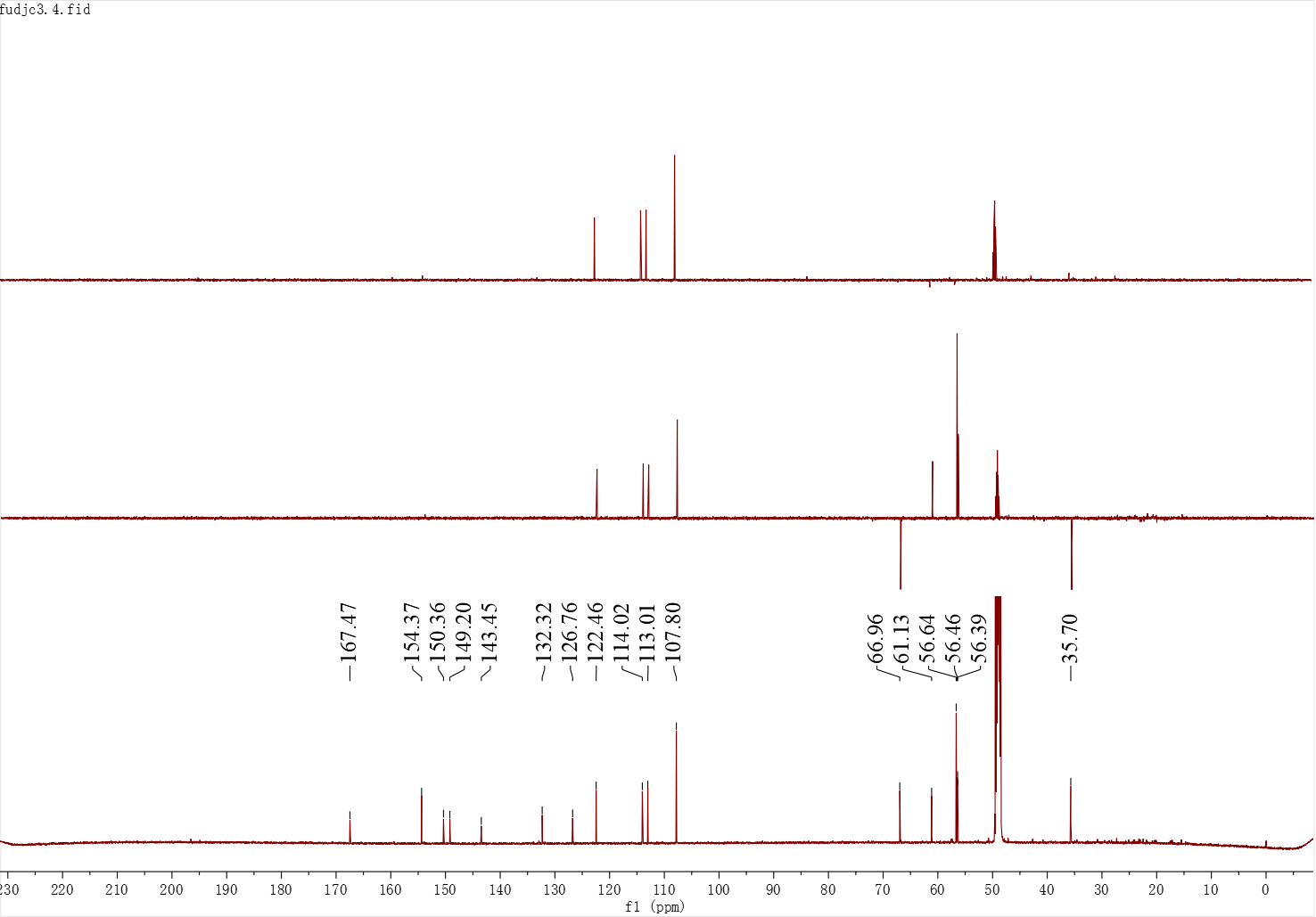


Figure 45 ^13^C NMR spectrum of compound **28** (150 MHz, CD_3_OD)

Figure 46 HSQC spectrum of compound **28**

Figure 47 HMBC spectrum of compound **28**

Figure 48 HRESIMS spectrum of compound **28**


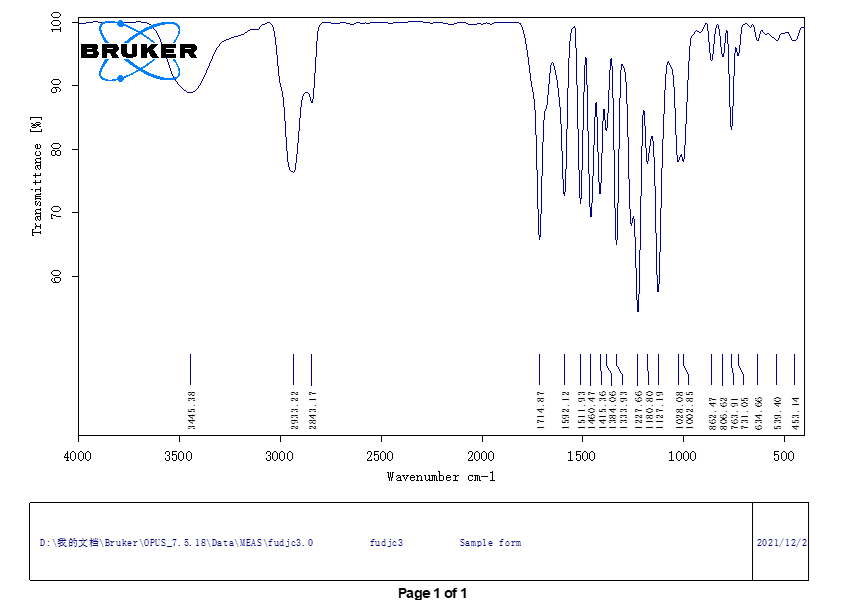


Figure 49 IR spectrum of compound **28**


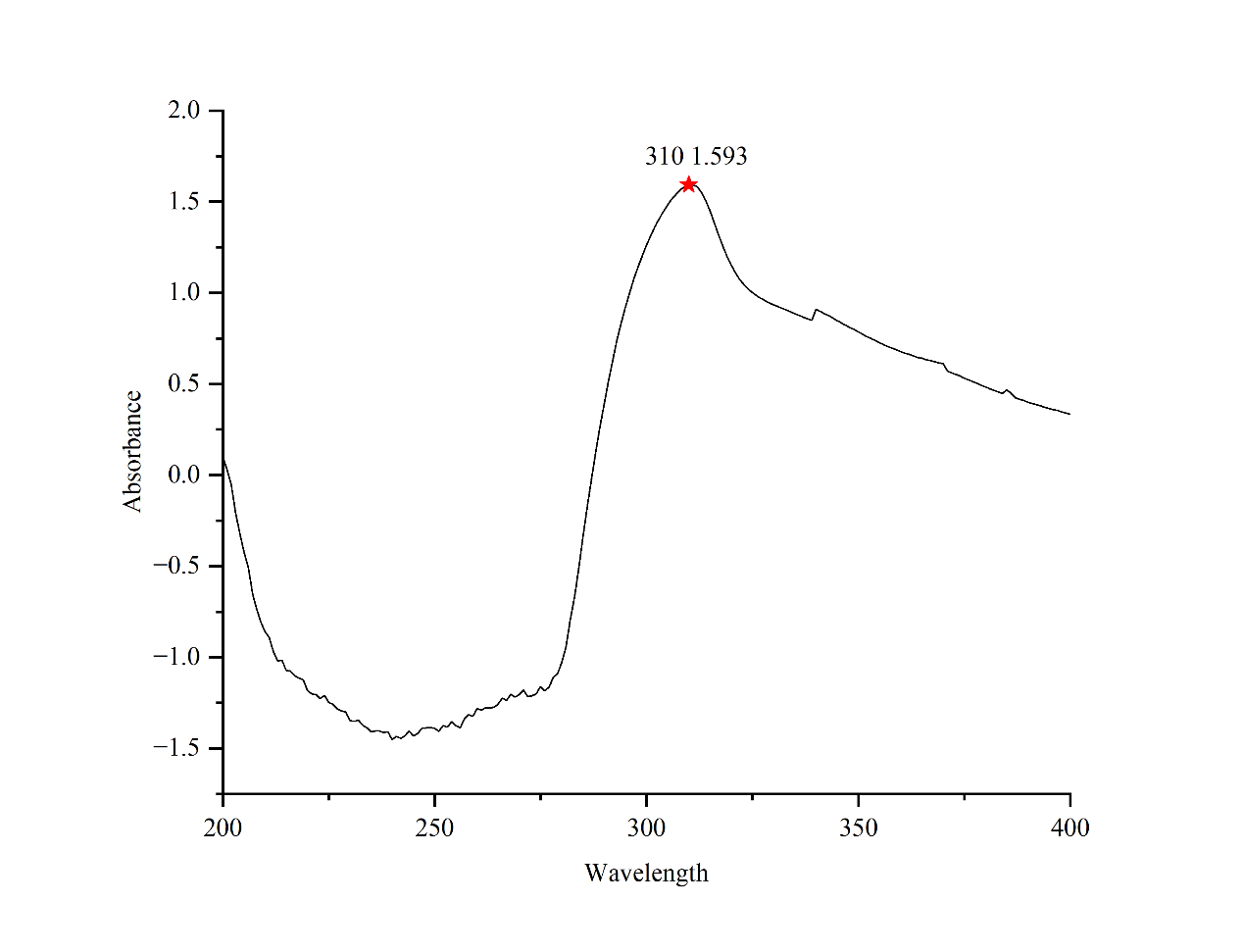


Figure 50 UV spectrum of compound **28**


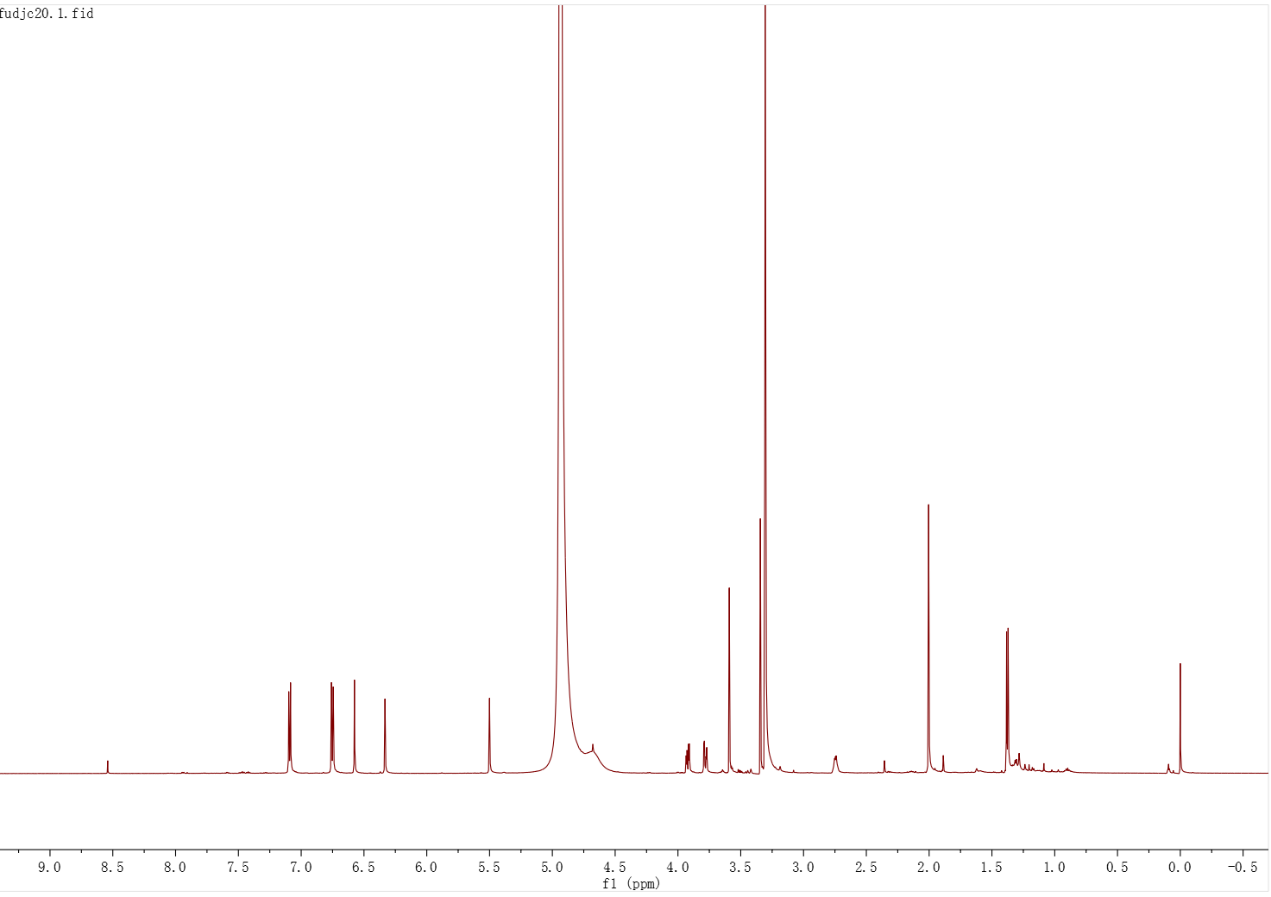


Figure 51 ^1^H NMR spectrum of compound **29** (600 MHz, CD_3_OD)


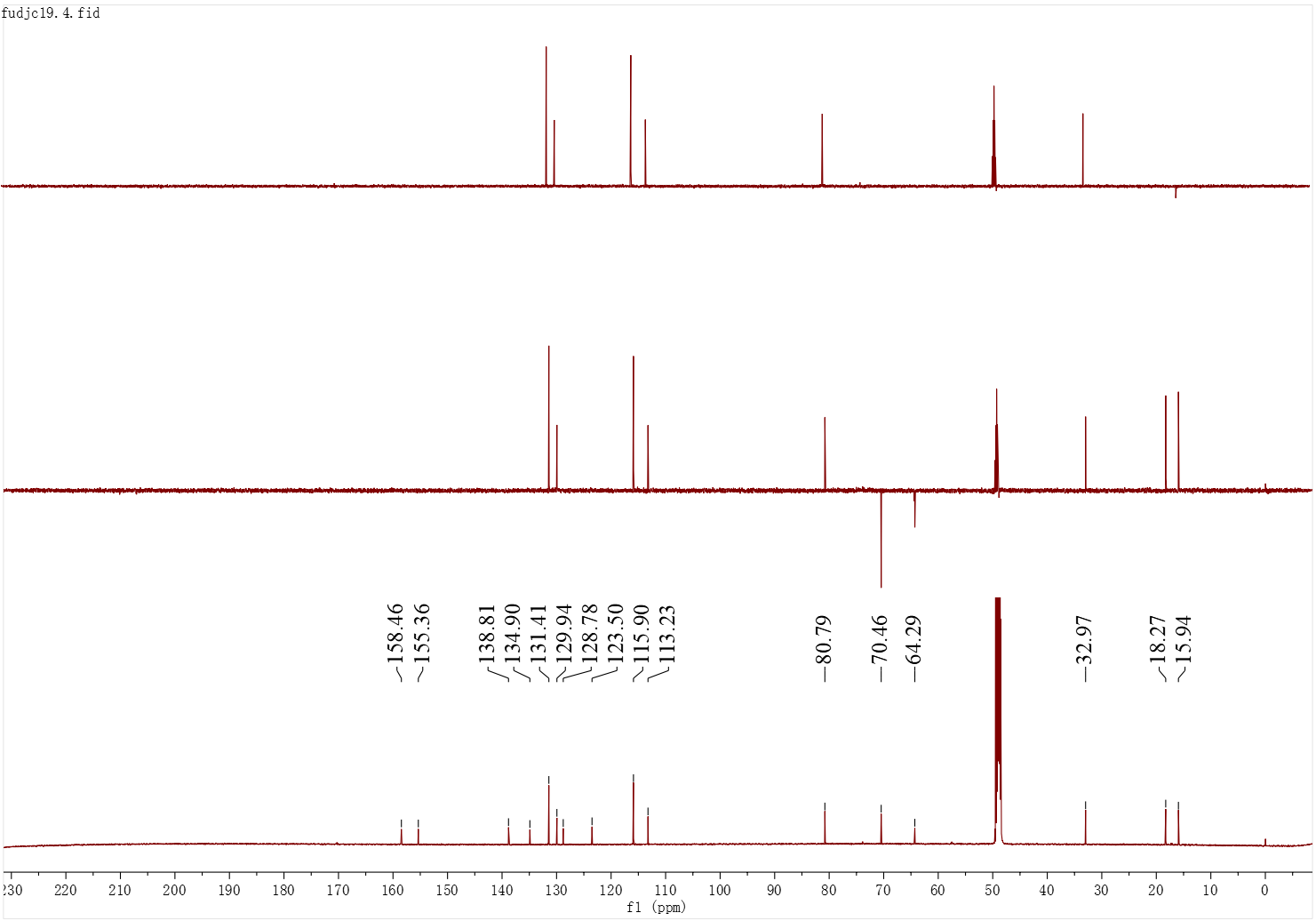


Figure 52 ^13^C NMR spectrum of compound **29** (150 MHz, CD_3_OD)


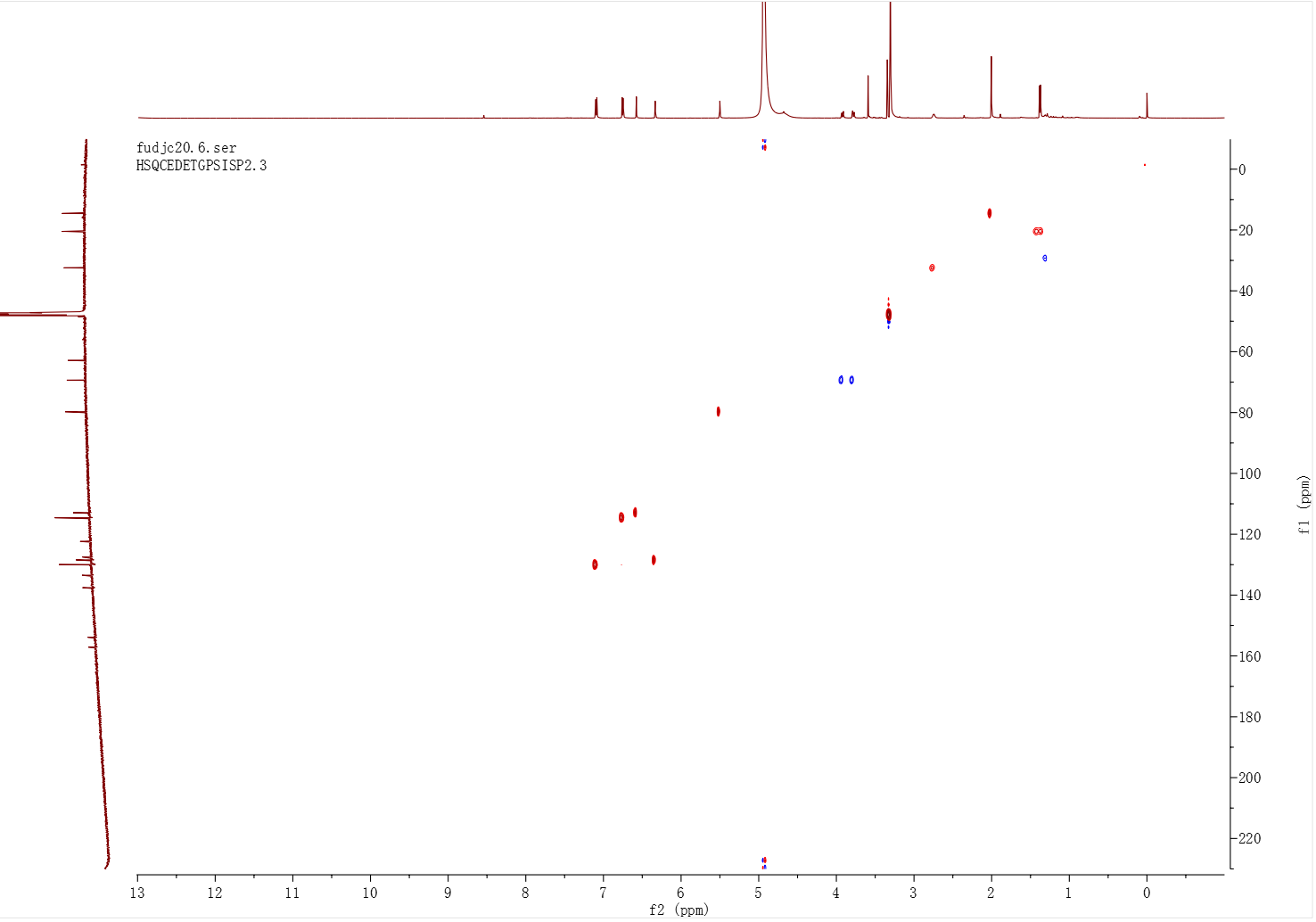


Figure 53 HSQC spectrum of compound **29**


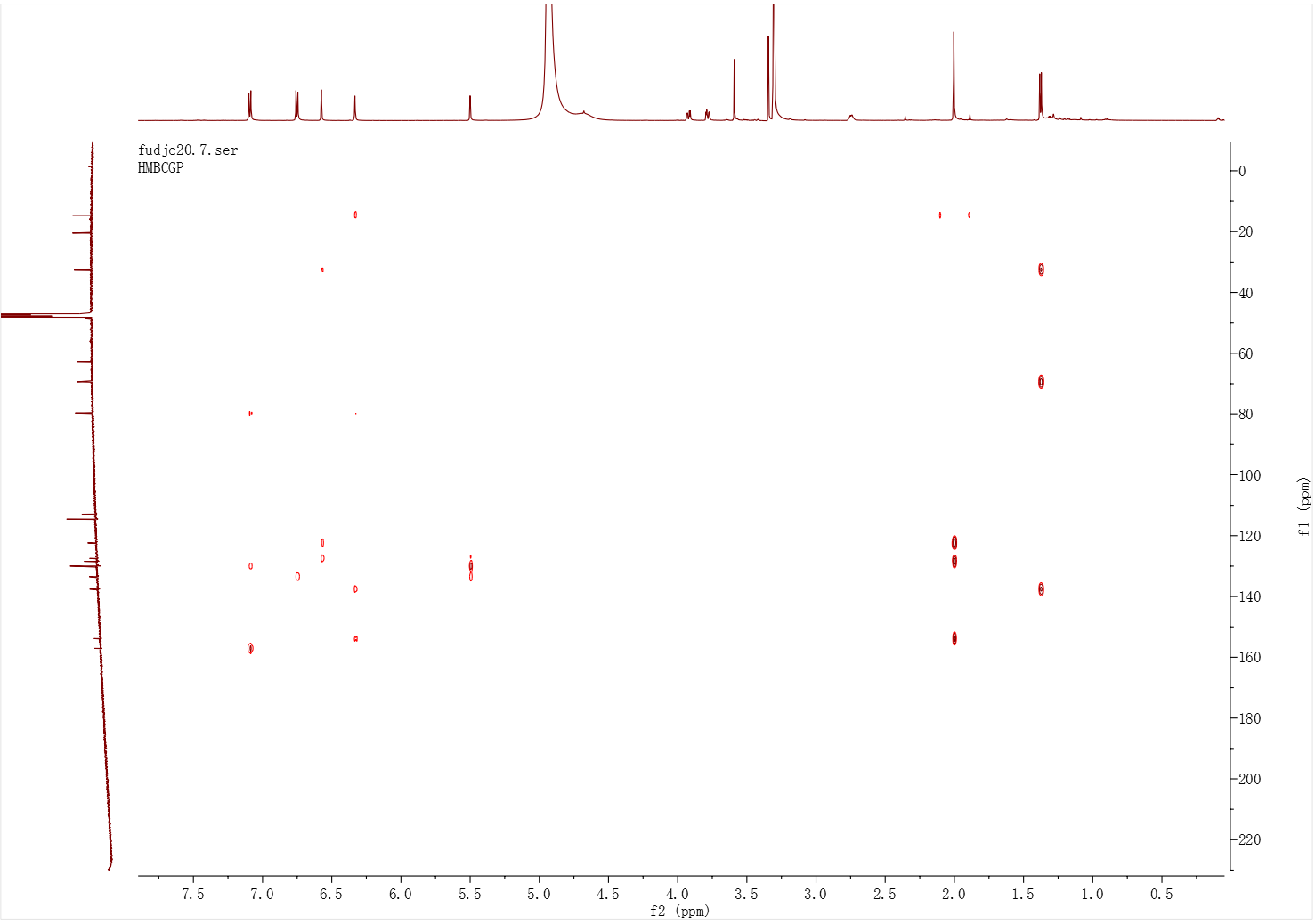


Figure 54 HMBC spectrum of compound **29**


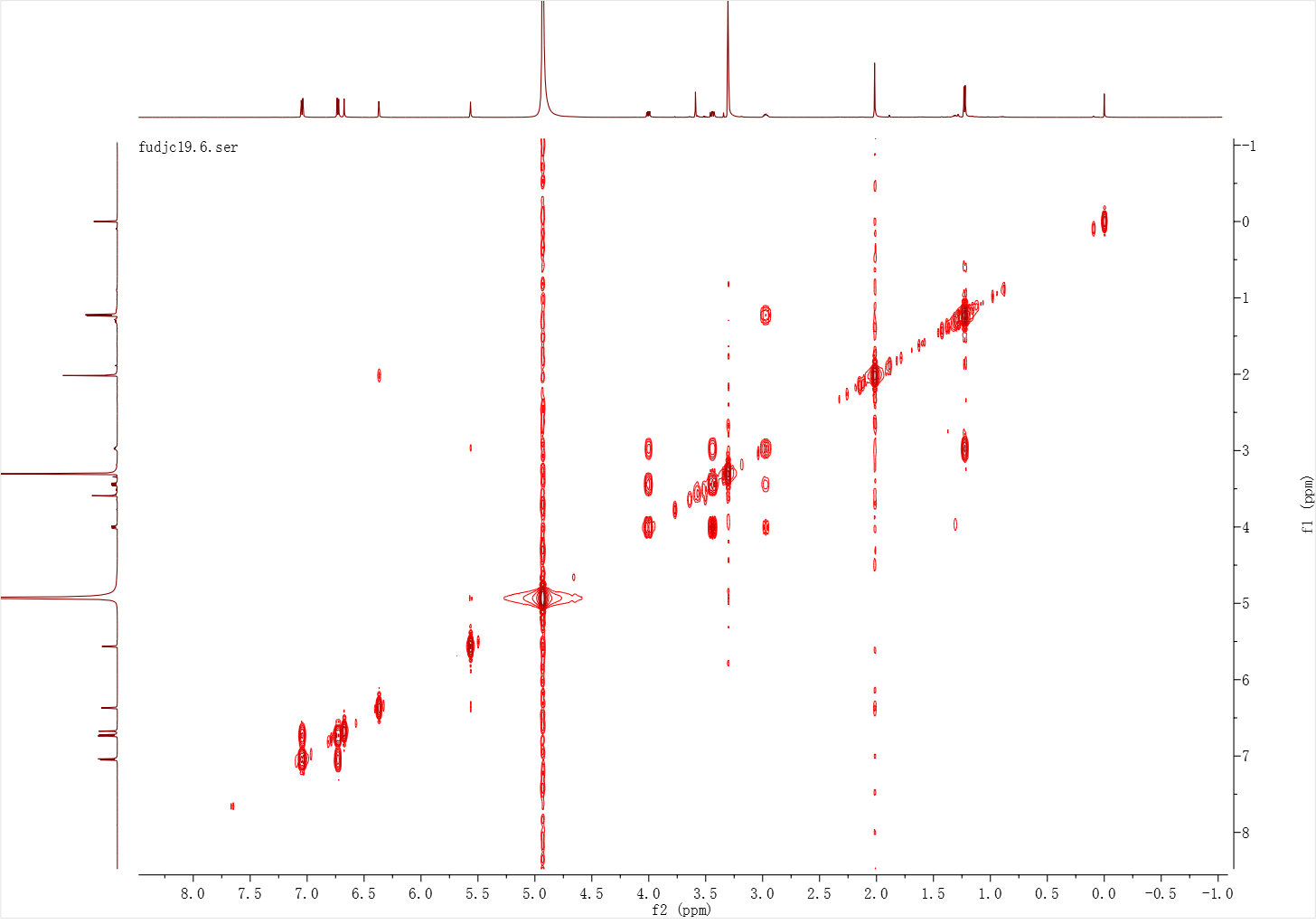


Figure 55 ROESY spectrum of compound **29**

Figure 56 HRESIMS spectrum of compound **29**


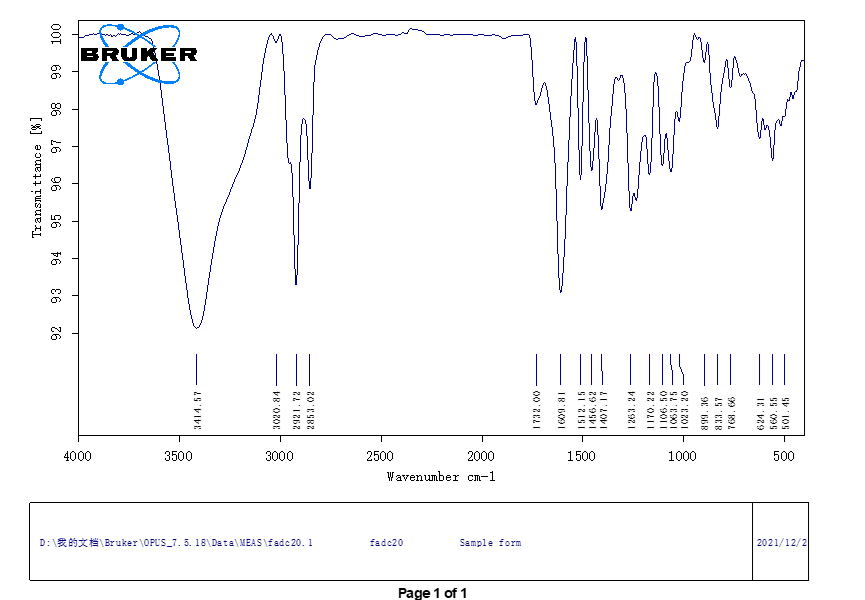


Figure 57 IR spectrum of compound **29**


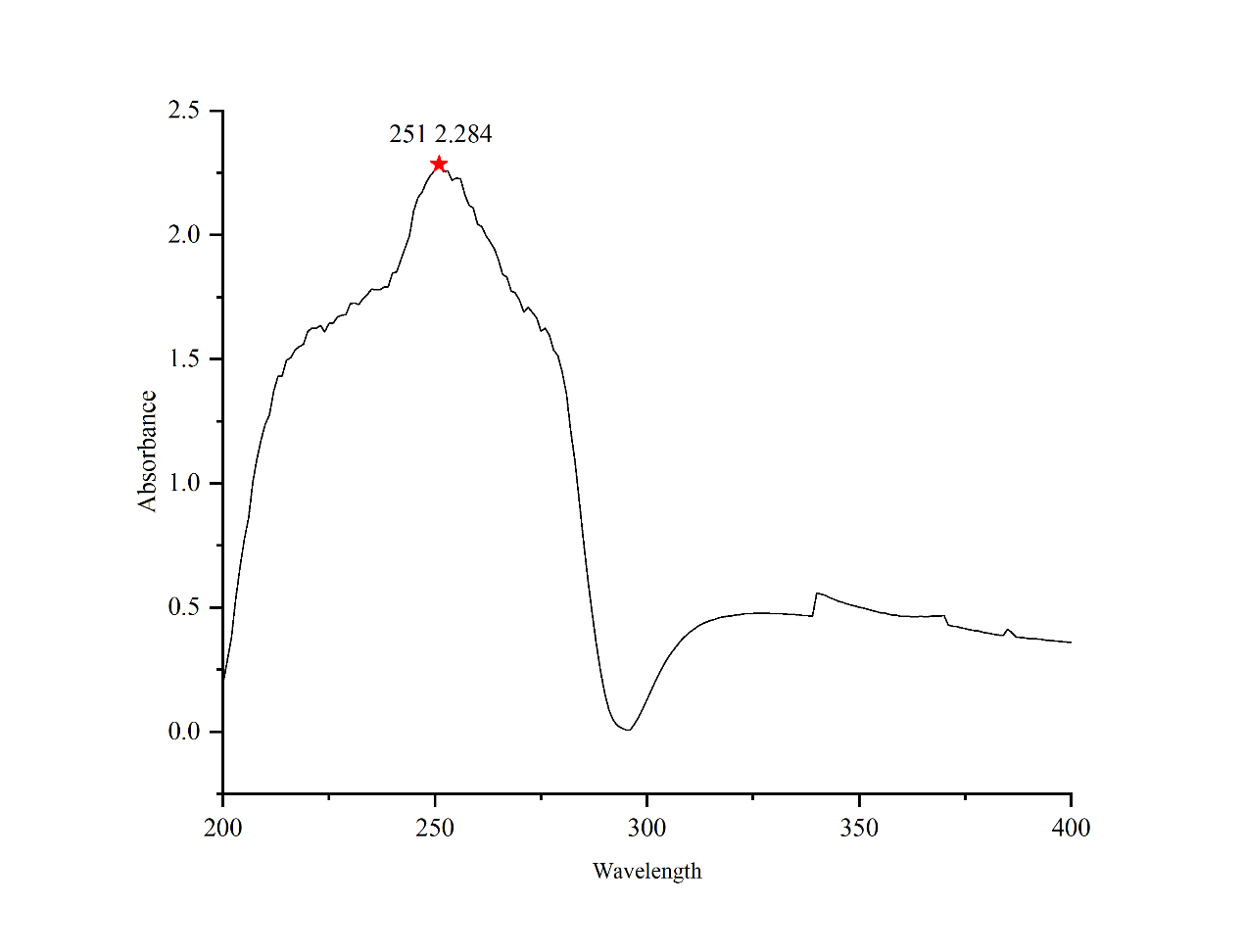


Figure 58 UV spectrum of compound **29**


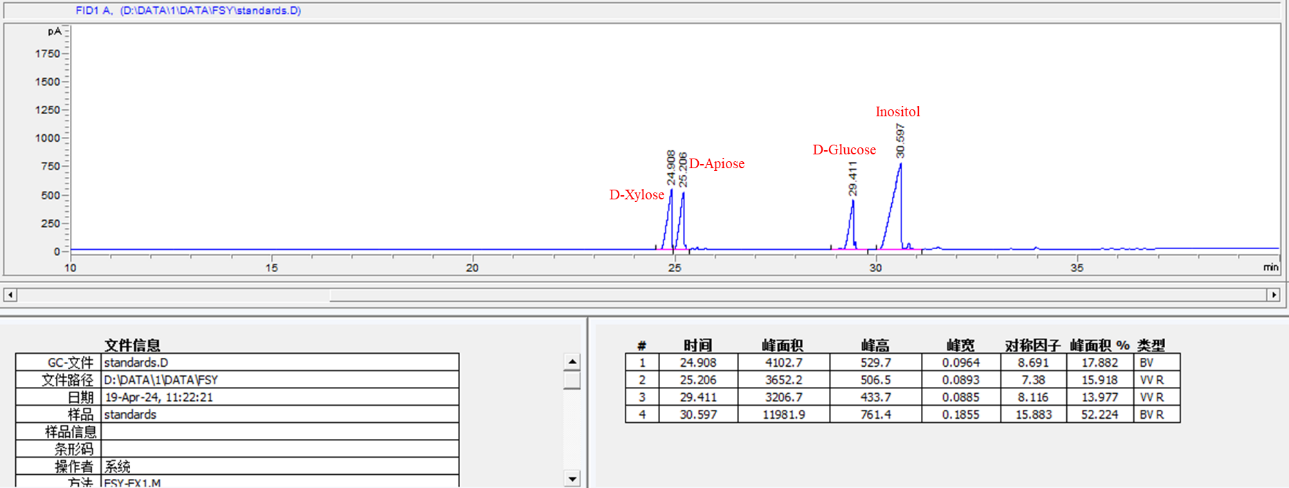


Figure 59 GC spectrum of standards


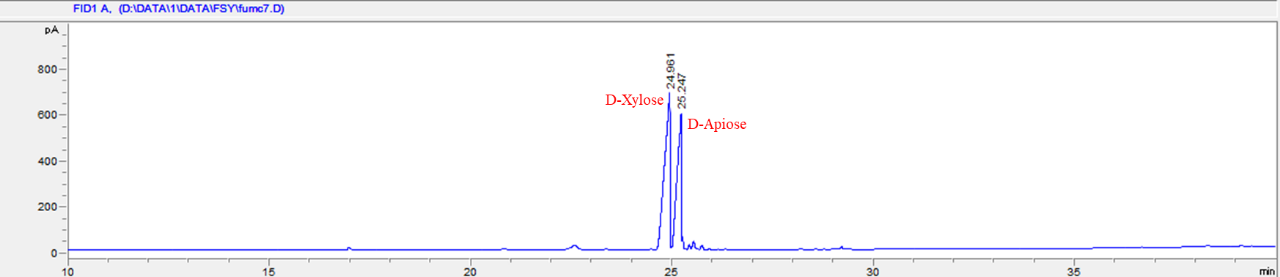


Figure 60 GC spectrum of compound **1**

**
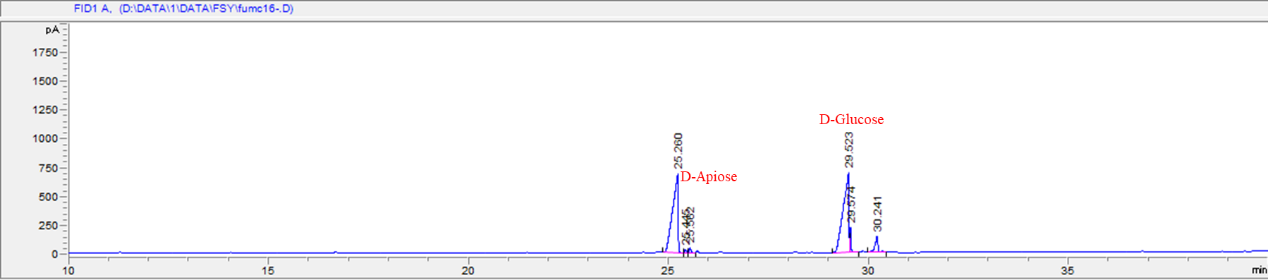
**

Figure 61 GC spectrum of compound **2**

**
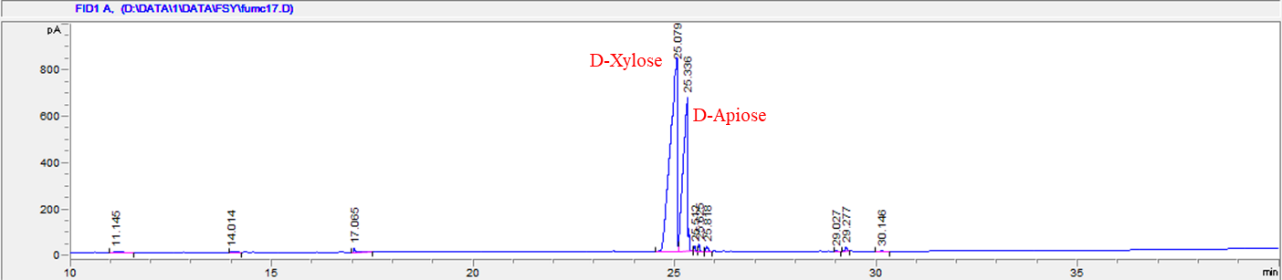
**

Figure 62 GC spectrum of compound **3**

**
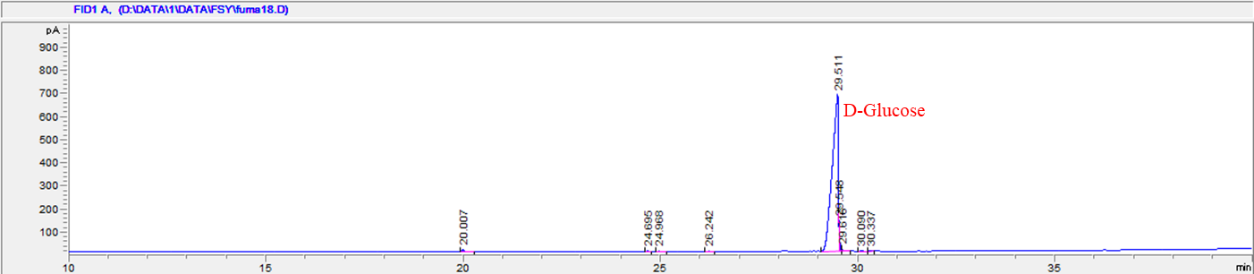
**

Figure 63 GC spectrum of compound **27**

Table S1 The energy distribution and Boltzmann weights of each conformer of compound **27**

| **State** | **GEPOP** | **SEPOP** | **Weighting factor** |
| --- | --- | --- | --- |
| **27a** | 56.04 | 55.76 | 17.66 |
| **27b** | 23.18 | 24.25 | 22.13 |
| **27c** | 4.14 | 4.56 | 7.52 |
| **27d** | 3.40 | 4.44 | 11.62 |
| **27e** | 2.41 | 3.88 | 21.26 |
| **27f** | 2.13 | 2.05 | 14.54 |
| **27g** | 1.26 | 0.87 | 1.59 |
| **27h** | 0.33 | 0.13 | 1.62 |
| **27i** | 0.15 | 0.07 | 2.06 |
| **total** | >93 | >96 | 100 |

(Notes: GEPOP = Distribution in GIBB’S Free Energy (%), SEPOP = Distribution on Steric Energy (%), read via CONFLEX. ECD Boltzmann weighting factor of each conformer was got by Specdis)

The distribution details of compound **27**:

(Notes: the conformers in the list corresponds to the results generated first after conformational search, and these conformers are the initial objects to perform calculations.)

Conformer **27a**


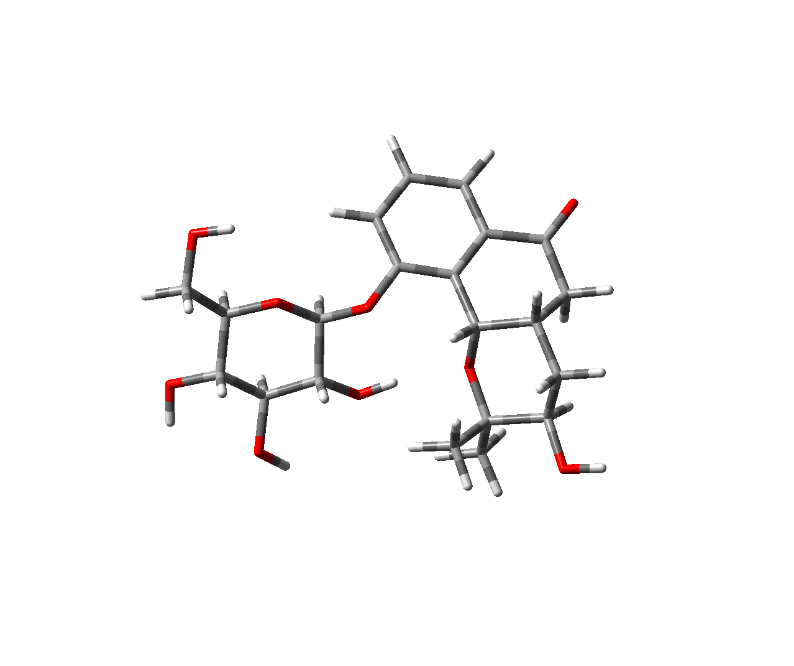


Table S2 Standard orientation of **27a**:

---------------------------------------------------------------------

Center Atomic Atomic Coordinates (Angstroms)

Number Number Type X Y Z

---------------------------------------------------------------------

1 6 0 0.300026 3.914955 -0.734012

2 6 0 -0.526170 2.890179 -0.267132

3 6 0 0.037225 1.675135 0.126499

4 6 0 1.424868 1.461600 0.098680

5 6 0 2.238456 2.508210 -0.379290

6 6 0 1.674931 3.724666 -0.793157

7 6 0 2.004418 0.156951 0.622933

8 6 0 3.488548 0.300103 0.994874

9 6 0 4.272307 0.975854 -0.142821

10 6 0 3.719714 2.343286 -0.479135

11 8 0 4.444226 3.261770 -0.843924

12 8 0 -0.736152 0.634176 0.592096

13 6 0 -1.941030 0.338032 -0.078668

14 8 0 -3.011225 0.768423 0.740137

15 6 0 -4.304103 0.474377 0.178260

16 6 0 -4.460108 -1.042086 0.010813

17 6 0 -3.325422 -1.588343 -0.855337

18 6 0 -1.970704 -1.177095 -0.288526

19 6 0 -5.318321 1.093100 1.132466

20 8 0 -5.129368 2.496093 1.250727

21 8 0 -3.485935 -3.002609 -0.906533

22 8 0 -0.965873 -1.567579 -1.210071

23 8 0 -5.722546 -1.298243 -0.589247

24 8 0 1.812870 -0.862790 -0.391069

25 6 0 2.282276 -2.211952 -0.061942

26 6 0 3.783568 -2.125881 0.304613

27 6 0 4.058360 -1.073210 1.381407

28 8 0 4.196427 -3.427140 0.726661

29 6 0 1.438197 -2.833155 1.059695

30 6 0 2.111682 -3.005133 -1.359590

31 1 0 -0.138517 4.859043 -1.042862

32 1 0 -1.599424 3.033019 -0.191697

33 1 0 2.336573 4.503745 -1.155392

34 1 0 1.441368 -0.130738 1.517599

35 1 0 3.536270 0.954484 1.875484

36 1 0 4.204118 0.371961 -1.058292

37 1 0 5.334332 1.078073 0.100029

38 1 0 -1.968017 0.851195 -1.050756

39 1 0 -4.394487 0.959924 -0.805504

40 1 0 -4.398622 -1.516378 1.003504

41 1 0 -3.424873 -1.161379 -1.865761

42 1 0 -1.828630 -1.655642 0.692068

43 1 0 -5.241669 0.594182 2.111183

44 1 0 -6.326592 0.932928 0.743575

45 1 0 -4.200461 2.618878 1.509252

46 1 0 -2.806754 -3.345380 -1.510547

47 1 0 -0.097234 -1.255549 -0.875559

48 1 0 -5.710168 -2.236752 -0.845012

49 1 0 4.322355 -1.852378 -0.614293

50 1 0 5.140554 -0.997275 1.550329

51 1 0 3.613482 -1.405579 2.327045

52 1 0 5.159403 -3.400326 0.845442

53 1 0 1.499169 -2.275898 1.998789

54 1 0 0.389506 -2.876911 0.751830

55 1 0 1.783317 -3.850968 1.257512

56 1 0 2.537760 -4.004752 -1.237818

57 1 0 1.054455 -3.103844 -1.622081

58 1 0 2.631094 -2.502042 -2.182057

--------------------------------------------------------------------

Conformer **27b**


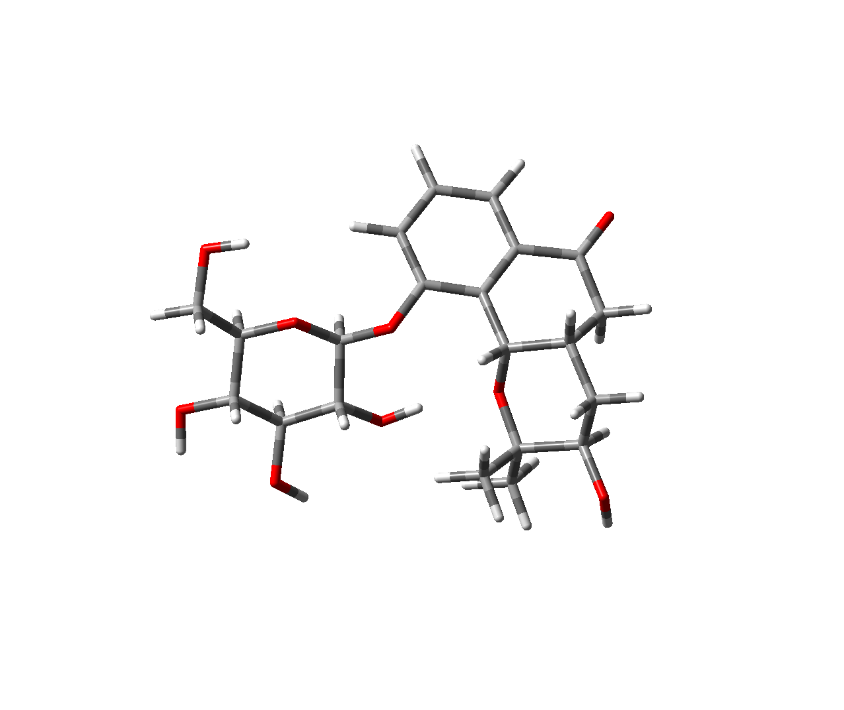


Table S3 Standard orientation of **27b**:

---------------------------------------------------------------------

Center Atomic Atomic Coordinates (Angstroms)

Number Number Type X Y Z

---------------------------------------------------------------------

1 6 0 0.293347 3.913880 -0.736120

2 6 0 -0.531437 2.888309 -0.268497

3 6 0 0.033737 1.674608 0.126609

4 6 0 1.421662 1.462690 0.098631

5 6 0 2.233775 2.510397 -0.379356

6 6 0 1.668582 3.725709 -0.794251

7 6 0 2.002633 0.158527 0.622699

8 6 0 3.486437 0.302427 0.995545

9 6 0 4.269887 0.981580 -0.140386

10 6 0 3.715344 2.347885 -0.477462

11 8 0 4.438701 3.267444 -0.841855

12 8 0 -0.738420 0.633394 0.593897

13 6 0 -1.942244 0.333982 -0.077111

14 8 0 -3.014220 0.768231 0.737261

15 6 0 -4.305572 0.471562 0.173177

16 6 0 -4.461169 -1.045726 0.012693

17 6 0 -3.324168 -1.596270 -0.847808

18 6 0 -1.971047 -1.182210 -0.279295

19 6 0 -5.322579 1.095051 1.121278

20 8 0 -5.134395 2.498687 1.232696

21 8 0 -3.484949 -3.010717 -0.892689

22 8 0 -0.963046 -1.576132 -1.195903

23 8 0 -5.721932 -1.304844 -0.589516

24 8 0 1.811323 -0.861729 -0.390828

25 6 0 2.288628 -2.207830 -0.057094

26 6 0 3.797943 -2.115224 0.305385

27 6 0 4.064774 -1.066924 1.381591

28 8 0 4.308967 -3.357178 0.791177

29 6 0 1.453036 -2.830830 1.069051

30 6 0 2.110117 -3.002236 -1.353799

31 1 0 -0.146484 4.856995 -1.046110

32 1 0 -1.604945 3.029420 -0.193545

33 1 0 2.329249 4.505635 -1.156446

34 1 0 1.439403 -0.129285 1.517122

35 1 0 3.532296 0.955844 1.876925

36 1 0 4.204543 0.378226 -1.056539

37 1 0 5.331324 1.085658 0.104109

38 1 0 -1.967578 0.842233 -1.051857

39 1 0 -4.393041 0.952219 -0.813247

40 1 0 -4.402351 -1.515166 1.007857

41 1 0 -3.420853 -1.174060 -1.860489

42 1 0 -1.831378 -1.655808 0.704040

43 1 0 -5.248591 0.601277 2.102797

44 1 0 -6.329708 0.932578 0.730381

45 1 0 -4.206788 2.623198 1.494987

46 1 0 -2.806529 -3.356465 -1.495824

47 1 0 -0.096579 -1.256538 -0.862615

48 1 0 -5.708517 -2.244415 -0.841324

49 1 0 4.333843 -1.832337 -0.611841

50 1 0 5.145625 -0.992043 1.547483

51 1 0 3.621886 -1.402122 2.327543

52 1 0 4.396291 -3.962809 0.038504

53 1 0 1.497340 -2.257692 1.999489

54 1 0 0.406601 -2.904675 0.759148

55 1 0 1.822869 -3.836160 1.286098

56 1 0 2.481154 -4.025262 -1.229446

57 1 0 1.054028 -3.063994 -1.630854

58 1 0 2.660529 -2.527844 -2.173137

---------------------------------------------------------------------

Conformer **27c**


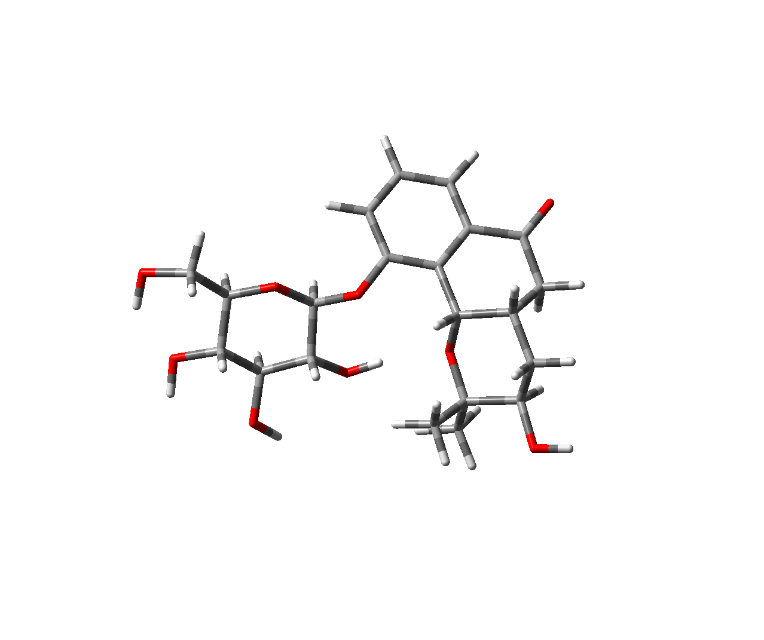


Table S4 Standard orientation of **27c**:

---------------------------------------------------------------------

Center Atomic Atomic Coordinates (Angstroms)

Number Number Type X Y Z

---------------------------------------------------------------------

1 6 0 0.478296 3.978214 -0.597163

2 6 0 -0.375755 2.965940 -0.153832

3 6 0 0.152470 1.722901 0.198913

4 6 0 1.532627 1.467405 0.153399

5 6 0 2.374727 2.502438 -0.300129

6 6 0 1.846127 3.747391 -0.673414

7 6 0 2.075351 0.130473 0.634537

8 6 0 3.566621 0.216414 0.996099

9 6 0 4.360942 0.901170 -0.128819

10 6 0 3.849314 2.295162 -0.417208

11 8 0 4.599725 3.201808 -0.758587

12 8 0 -0.649983 0.693224 0.638978

13 6 0 -1.868805 0.455377 -0.030782

14 8 0 -2.916982 0.914168 0.801819

15 6 0 -4.217568 0.671714 0.248190

16 6 0 -4.413435 -0.841402 0.059622

17 6 0 -3.317837 -1.418453 -0.831535

18 6 0 -1.946756 -1.053804 -0.271930

19 6 0 -5.236481 1.284933 1.214671

20 8 0 -6.546080 1.296193 0.668887

21 8 0 -3.523104 -2.826061 -0.888944

22 8 0 -0.962228 -1.454613 -1.210525

23 8 0 -5.702913 -1.055202 -0.515713

24 8 0 1.842870 -0.853372 -0.406119

25 6 0 2.274055 -2.225274 -0.121094

26 6 0 3.780730 -2.195491 0.231630

27 6 0 4.097321 -1.184485 1.336382

28 8 0 4.158293 -3.520712 0.610024

29 6 0 1.422766 -2.853172 0.991339

30 6 0 2.066311 -2.974533 -1.439320

31 1 0 0.067108 4.944226 -0.874275

32 1 0 -1.443275 3.138792 -0.063063

33 1 0 2.528850 4.516096 -1.018271

34 1 0 1.511269 -0.165189 1.525963

35 1 0 3.642598 0.842591 1.895018

36 1 0 4.264333 0.328133 -1.061451

37 1 0 5.427977 0.961873 0.105803

38 1 0 -1.880006 0.988214 -0.992732

39 1 0 -4.314262 1.171838 -0.727959

40 1 0 -4.353796 -1.325155 1.046609

41 1 0 -3.415620 -0.979462 -1.836732

42 1 0 -1.809706 -1.556344 0.697354

43 1 0 -4.955852 2.324872 1.408446

44 1 0 -5.193921 0.741855 2.172587

45 1 0 -6.712329 0.391925 0.342026

46 1 0 -2.852427 -3.188044 -1.491248

47 1 0 -0.081704 -1.175511 -0.877787

48 1 0 -5.786307 -2.011722 -0.670234

49 1 0 4.318517 -1.910487 -0.684353

50 1 0 5.182784 -1.146433 1.496783

51 1 0 3.651140 -1.531264 2.276218

52 1 0 5.123017 -3.526832 0.716965

53 1 0 1.508933 -2.324732 1.945022

54 1 0 0.370340 -2.857860 0.693374

55 1 0 1.739914 -3.885714 1.156953

56 1 0 2.462421 -3.989837 -1.351115

57 1 0 1.004046 -3.033018 -1.693429

58 1 0 2.593051 -2.463787 -2.252366

---------------------------------------------------------------------

Conformer **27d**


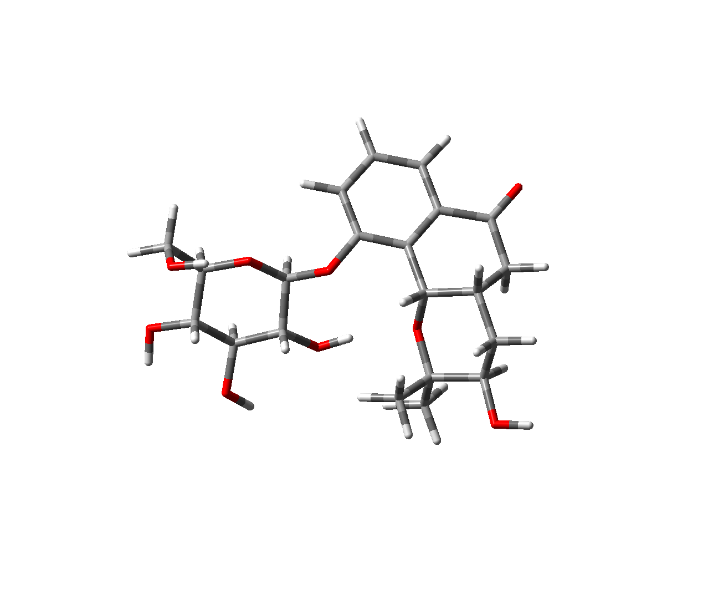


Table S5 Standard orientation of **27d**:

---------------------------------------------------------------------

Center Atomic Atomic Coordinates (Angstroms)

Number Number Type X Y Z

---------------------------------------------------------------------

1 6 0 0.542235 3.974149 -0.713776

2 6 0 -0.351390 2.990712 -0.283784

3 6 0 0.133029 1.740440 0.104516

4 6 0 1.506862 1.449536 0.107883

5 6 0 2.389501 2.455742 -0.333185

6 6 0 1.905407 3.707689 -0.741788

7 6 0 1.999272 0.106955 0.625975

8 6 0 3.479644 0.160981 1.035076

9 6 0 4.327455 0.807978 -0.073155

10 6 0 3.861330 2.209542 -0.399192

11 8 0 4.645278 3.091451 -0.729871

12 8 0 -0.709764 0.738611 0.533616

13 6 0 -1.911384 0.520766 -0.173413

14 8 0 -2.973404 1.020292 0.615772

15 6 0 -4.278332 0.787605 0.052482

16 6 0 -4.501474 -0.717866 -0.149355

17 6 0 -3.383600 -1.310186 -1.003807

18 6 0 -2.023669 -0.987540 -0.397362

19 6 0 -5.262096 1.418591 1.033798

20 8 0 -5.156086 0.852544 2.333606

21 8 0 -3.621034 -2.712472 -1.087940

22 8 0 -1.018065 -1.427274 -1.295891

23 8 0 -5.763818 -0.891977 -0.780930

24 8 0 1.775535 -0.886447 -0.407436

25 6 0 2.161961 -2.264242 -0.088638

26 6 0 3.656638 -2.266989 0.313126

27 6 0 3.963043 -1.247365 1.412909

28 8 0 3.987271 -3.595416 0.723018

29 6 0 1.259138 -2.853433 1.004166

30 6 0 1.977799 -3.027982 -1.402028

31 1 0 0.165184 4.945793 -1.018414

32 1 0 -1.416558 3.191656 -0.230858

33 1 0 2.618445 4.453507 -1.075245

34 1 0 1.399070 -0.160590 1.502631

35 1 0 3.542320 0.798971 1.926713

36 1 0 4.246715 0.222771 -0.999703

37 1 0 5.387507 0.845626 0.195563

38 1 0 -1.877195 1.039789 -1.142219

39 1 0 -4.364486 1.294930 -0.920806

40 1 0 -4.490186 -1.205270 0.836356

41 1 0 -3.434564 -0.859399 -2.007749

42 1 0 -1.936412 -1.482850 0.581352

43 1 0 -6.282673 1.239313 0.687077

44 1 0 -5.087501 2.504257 1.057656

45 1 0 -4.221876 0.938707 2.588603

46 1 0 -2.939672 -3.081925 -1.673553

47 1 0 -0.142314 -1.166721 -0.936787

48 1 0 -5.802344 -1.827711 -1.043377

49 1 0 4.231206 -2.009833 -0.588635

50 1 0 5.043357 -1.233980 1.608169

51 1 0 3.478050 -1.568409 2.342557

52 1 0 4.948013 -3.625623 0.858001

53 1 0 1.543822 -3.891109 1.194749

54 1 0 1.328363 -2.313073 1.952514

55 1 0 0.217135 -2.835718 0.671994

56 1 0 2.344196 -4.051676 -1.286367

57 1 0 0.923136 -3.063139 -1.689850

58 1 0 2.543734 -2.543287 -2.204609

---------------------------------------------------------------------

Conformer **27e**


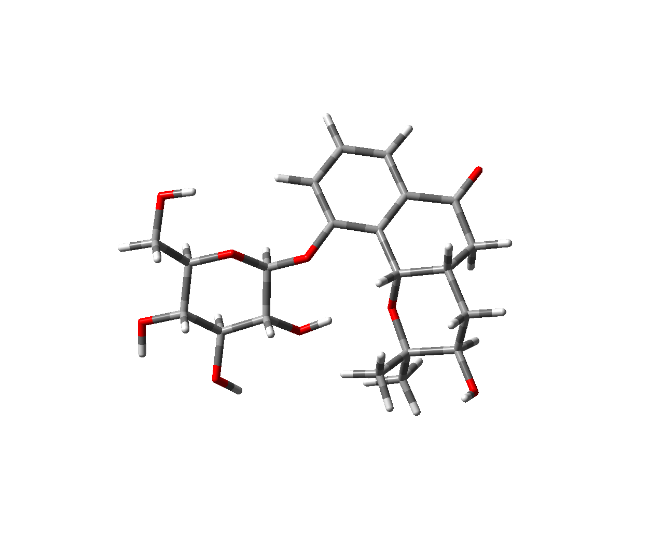


Table S6 Standard orientation of **27e**:

---------------------------------------------------------------------

Center Atomic Atomic Coordinates (Angstroms)

Number Number Type X Y Z

---------------------------------------------------------------------

1 6 0 0.296358 3.915956 -0.725029

2 6 0 -0.528473 2.890086 -0.258177

3 6 0 0.036207 1.674637 0.132367

4 6 0 1.423842 1.462075 0.102028

5 6 0 2.235989 2.509218 -0.377193

6 6 0 1.671173 3.726079 -0.787978

7 6 0 2.005201 0.156687 0.622574

8 6 0 3.490266 0.301067 0.990128

9 6 0 4.269919 0.975432 -0.150764

10 6 0 3.716745 2.343709 -0.483308

11 8 0 4.440377 3.262049 -0.849825

12 8 0 -0.736111 0.631959 0.596075

13 6 0 -1.940230 0.336096 -0.076402

14 8 0 -3.011419 0.766501 0.740979

15 6 0 -4.303673 0.473729 0.176921

16 6 0 -4.460384 -1.042526 0.008461

17 6 0 -3.324397 -1.589223 -0.855724

18 6 0 -1.970293 -1.179002 -0.286470

19 6 0 -5.319006 1.092604 1.129852

20 8 0 -5.129688 2.495460 1.248990

21 8 0 -3.485830 -3.003382 -0.907507

22 8 0 -0.964266 -1.571064 -1.206148

23 8 0 -5.721755 -1.297513 -0.594265

24 8 0 1.811810 -0.860238 -0.393330

25 6 0 2.287308 -2.209339 -0.070037

26 6 0 3.793401 -2.125862 0.304869

27 6 0 4.067411 -1.069288 1.376372

28 8 0 4.317404 -3.401925 0.673728

29 6 0 1.438131 -2.839229 1.044128

30 6 0 2.125386 -2.997063 -1.371090

31 1 0 -0.143197 4.860345 -1.031486

32 1 0 -1.601704 3.032030 -0.180871

33 1 0 2.331693 4.505624 -1.151257

34 1 0 1.444599 -0.133333 1.518309

35 1 0 3.540499 0.956405 1.869841

36 1 0 4.196536 0.371621 -1.065993

37 1 0 5.333026 1.076422 0.087443

38 1 0 -1.965681 0.849511 -1.048424

39 1 0 -4.392115 0.959819 -0.806734

40 1 0 -4.401208 -1.517415 1.001016

41 1 0 -3.421693 -1.161999 -1.866210

42 1 0 -1.830601 -1.657485 0.694559

43 1 0 -5.244096 0.593236 2.108467

44 1 0 -6.326754 0.933068 0.739351

45 1 0 -4.201623 2.617851 1.510658

46 1 0 -2.808057 -3.346334 -1.512986

47 1 0 -0.096561 -1.252437 -0.875377

48 1 0 -5.709350 -2.235800 -0.850867

49 1 0 4.337245 -1.862632 -0.608806

50 1 0 5.148631 -0.997438 1.541686

51 1 0 3.622566 -1.390202 2.328747

52 1 0 4.033531 -3.589596 1.583319

53 1 0 1.508455 -2.303643 1.995714

54 1 0 0.387797 -2.861606 0.740990

55 1 0 1.755061 -3.872502 1.216152

56 1 0 2.567517 -3.990657 -1.255157

57 1 0 1.069589 -3.110936 -1.633558

58 1 0 2.636442 -2.481751 -2.191286

---------------------------------------------------------------------

Conformer **27f**


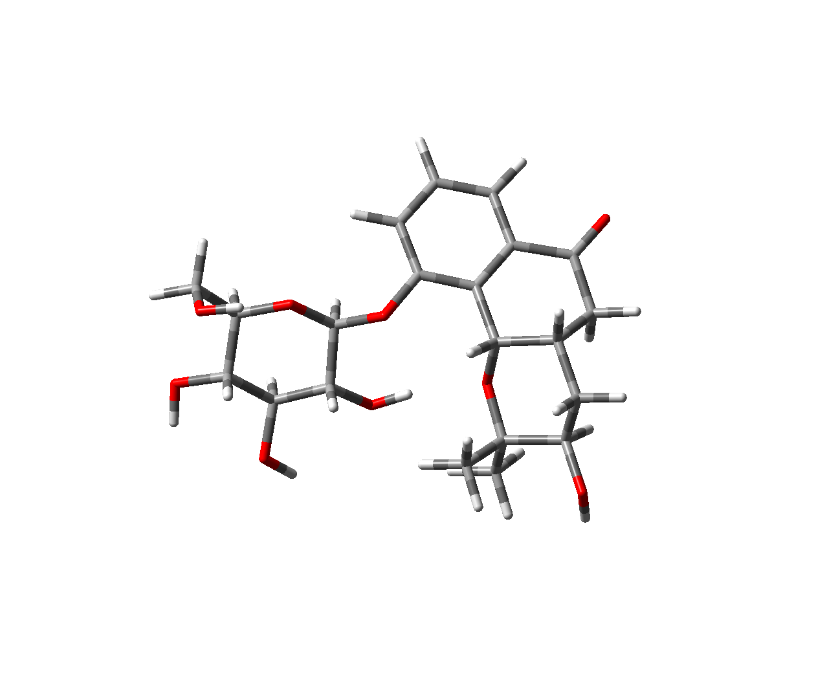


Table S7 Standard orientation of **27f**:

---------------------------------------------------------------------

Center Atomic Atomic Coordinates (Angstroms)

Number Number Type X Y Z

---------------------------------------------------------------------

1 6 0 0.542800 3.975171 -0.717520

2 6 0 -0.351741 2.992837 -0.286809

3 6 0 0.131744 1.742695 0.102897

4 6 0 1.505364 1.450668 0.106322

5 6 0 2.388930 2.455916 -0.334818

6 6 0 1.905842 3.707830 -0.744653

7 6 0 1.996261 0.107781 0.624773

8 6 0 3.475453 0.160588 1.037618

9 6 0 4.326016 0.807740 -0.068363

10 6 0 3.860627 2.208568 -0.398590

11 8 0 4.645363 3.089105 -0.731088

12 8 0 -0.711562 0.742016 0.533728

13 6 0 -1.912609 0.521921 -0.173425

14 8 0 -2.975614 1.020985 0.614645

15 6 0 -4.279844 0.785824 0.050759

16 6 0 -4.500827 -0.720151 -0.149940

17 6 0 -3.381458 -1.312077 -1.002809

18 6 0 -2.022683 -0.986930 -0.395257

19 6 0 -5.265102 1.416122 1.031041

20 8 0 -5.159004 0.851222 2.331326

21 8 0 -3.617318 -2.714692 -1.085561

22 8 0 -1.014827 -1.425969 -1.291647

23 8 0 -5.762363 -0.896348 -0.782451

24 8 0 1.774190 -0.885466 -0.409031

25 6 0 2.164114 -2.261572 -0.083990

26 6 0 3.665369 -2.262569 0.321645

27 6 0 3.963266 -1.244695 1.418705

28 8 0 4.087702 -3.537911 0.806317

29 6 0 1.262875 -2.851516 1.008746

30 6 0 1.976430 -3.025011 -1.397991

31 1 0 0.166598 4.946825 -1.023176

32 1 0 -1.416774 3.194650 -0.234366

33 1 0 2.619465 4.453069 -1.078153

34 1 0 1.393710 -0.159510 1.499780

35 1 0 3.535694 0.798987 1.929082

36 1 0 4.249055 0.221363 -0.994615

37 1 0 5.385154 0.846244 0.203743

38 1 0 -1.878700 1.039734 -1.142897

39 1 0 -4.366154 1.292204 -0.923002

40 1 0 -4.489788 -1.206626 0.836243

41 1 0 -3.432032 -0.862419 -2.007272

42 1 0 -1.935821 -1.480616 0.584298

43 1 0 -6.285216 1.235045 0.683898

44 1 0 -5.092181 2.502077 1.054138

45 1 0 -4.225100 0.939093 2.586869

46 1 0 -2.936344 -3.083926 -1.671733

47 1 0 -0.140348 -1.161444 -0.932089

48 1 0 -5.799596 -1.832386 -1.043995

49 1 0 4.243645 -2.001931 -0.576320

50 1 0 5.041862 -1.234910 1.613421

51 1 0 3.476918 -1.565565 2.348116

52 1 0 4.156222 -4.141031 0.049725

53 1 0 1.316006 -2.297103 1.950022

54 1 0 0.222696 -2.858127 0.670470

55 1 0 1.566776 -3.880318 1.217451

56 1 0 2.285465 -4.069365 -1.281304

57 1 0 0.926419 -3.021971 -1.703734

58 1 0 2.574897 -2.570423 -2.194537

---------------------------------------------------------------------

Conformer **27g**


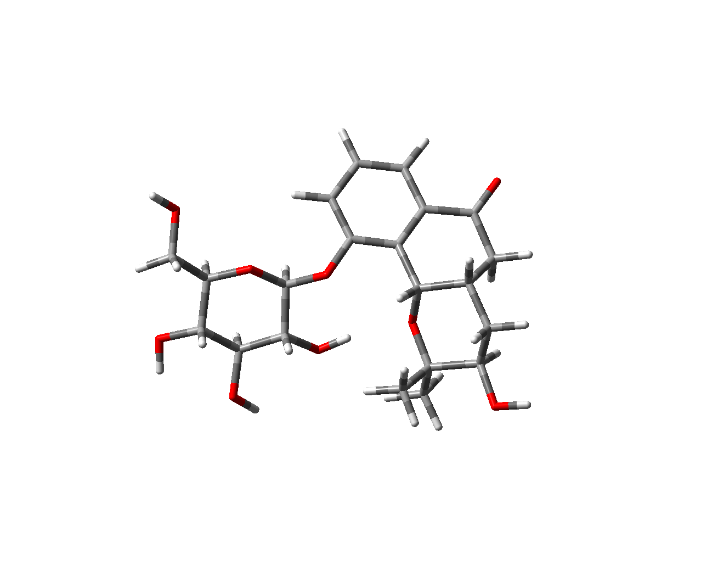


Table S8 Standard orientation of **27g**:

---------------------------------------------------------------------

Center Atomic Atomic Coordinates (Angstroms)

Number Number Type X Y Z

---------------------------------------------------------------------

1 6 0 0.216322 3.900649 -0.665162

2 6 0 -0.578616 2.847544 -0.206981

3 6 0 0.021055 1.641748 0.161140

4 6 0 1.413409 1.464742 0.118556

5 6 0 2.194391 2.539261 -0.351984

6 6 0 1.594960 3.746763 -0.741534

7 6 0 2.030749 0.168701 0.622032

8 6 0 3.517302 0.343575 0.972273

9 6 0 4.266605 1.053617 -0.167711

10 6 0 3.677571 2.413167 -0.472489

11 8 0 4.375431 3.355259 -0.829270

12 8 0 -0.721433 0.572556 0.610711

13 6 0 -1.928069 0.271004 -0.056127

14 8 0 -2.996750 0.743478 0.736008

15 6 0 -4.284048 0.456285 0.165517

16 6 0 -4.461028 -1.066523 0.052206

17 6 0 -3.323946 -1.659413 -0.780417

18 6 0 -1.968516 -1.248844 -0.217486

19 6 0 -5.333251 1.105604 1.049430

20 8 0 -5.210574 2.518670 0.923304

21 8 0 -3.509511 -3.072095 -0.784633

22 8 0 -0.960005 -1.677229 -1.119302

23 8 0 -5.715614 -1.338490 -0.557666

24 8 0 1.844061 -0.847831 -0.395427

25 6 0 2.352628 -2.186924 -0.086093

26 6 0 3.858770 -2.066284 0.248606

27 6 0 4.128583 -1.019193 1.331889

28 8 0 4.315256 -3.360659 0.645931

29 6 0 1.548854 -2.837318 1.048870

30 6 0 2.173454 -2.975360 -1.385584

31 1 0 -0.249250 4.838006 -0.954483

32 1 0 -1.654314 2.958334 -0.115127

33 1 0 2.232361 4.547912 -1.099133

34 1 0 1.488951 -0.137560 1.523875

35 1 0 3.562886 0.987758 1.860510

36 1 0 4.196989 0.462226 -1.091251

37 1 0 5.329906 1.176427 0.059572

38 1 0 -1.938231 0.751318 -1.046082

39 1 0 -4.350571 0.898716 -0.840752

40 1 0 -4.418246 -1.503076 1.063017

41 1 0 -3.408006 -1.266194 -1.805830

42 1 0 -1.837569 -1.698436 0.778266

43 1 0 -5.175950 0.778101 2.088176

44 1 0 -6.321207 0.754494 0.724064

45 1 0 -5.896339 2.919736 1.479526

46 1 0 -2.845792 -3.447009 -1.386484

47 1 0 -0.099123 -1.320483 -0.812239

48 1 0 -5.696090 -2.282905 -0.791202

49 1 0 4.369707 -1.767781 -0.678474

50 1 0 5.211732 -0.917747 1.480522

51 1 0 3.711231 -1.373399 2.282131

52 1 0 5.279172 -3.308382 0.747129

53 1 0 1.617161 -2.286004 1.990992

54 1 0 0.495072 -2.904295 0.763644

55 1 0 1.922987 -3.847932 1.230597

56 1 0 2.627497 -3.964540 -1.280643

57 1 0 1.113608 -3.099871 -1.625727

58 1 0 2.661467 -2.453470 -2.215543

---------------------------------------------------------------------

Conformer **27h**


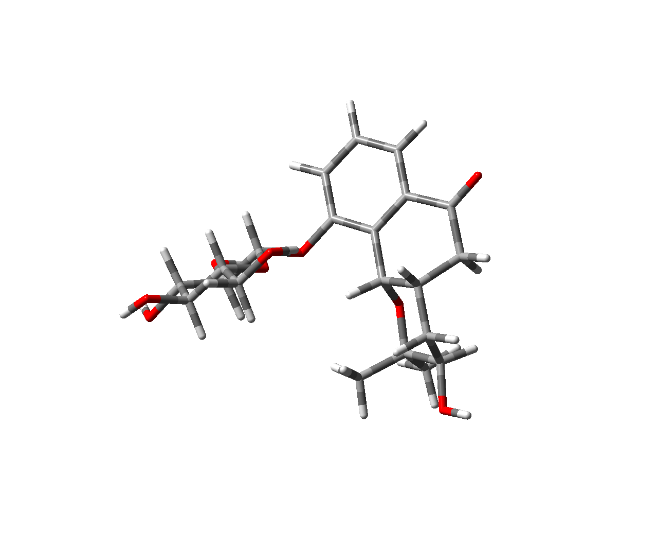


Table S9 Standard orientation of **27h**:

---------------------------------------------------------------------

Center Atomic Atomic Coordinates (Angstroms)

Number Number Type X Y Z

---------------------------------------------------------------------

1 6 0 0.826427 3.965414 -1.095253

2 6 0 -0.112244 2.967313 -1.367298

3 6 0 0.137019 1.653268 -0.970497

4 6 0 1.324985 1.298785 -0.315331

5 6 0 2.265589 2.316378 -0.058129

6 6 0 2.007861 3.641770 -0.438454

7 6 0 1.574211 -0.139442 0.090631

8 6 0 2.587229 -0.263835 1.237502

9 6 0 3.851924 0.552666 0.921893

10 6 0 3.552461 2.010306 0.637786

11 8 0 4.342387 2.898477 0.937898

12 8 0 -0.780592 0.654247 -1.275490

13 6 0 -2.023320 0.707566 -0.620011

14 8 0 -1.846268 0.207053 0.697729

15 6 0 -3.056161 0.203921 1.475704

16 6 0 -4.079062 -0.725055 0.812755

17 6 0 -4.312331 -0.285729 -0.632227

18 6 0 -2.994879 -0.183932 -1.397113

19 6 0 -2.645370 -0.229909 2.877208

20 8 0 -1.699605 0.668676 3.439467

21 8 0 -5.196496 -1.232675 -1.220361

22 8 0 -3.294242 0.353517 -2.677608

23 8 0 -5.286394 -0.671023 1.559938

24 8 0 2.056013 -0.821811 -1.080819

25 6 0 2.233340 -2.255501 -0.949177

26 6 0 3.250573 -2.501622 0.192285

27 6 0 2.874568 -1.756387 1.481041

28 8 0 3.330306 -3.912106 0.412627

29 6 0 0.891502 -2.966838 -0.716047

30 6 0 2.826373 -2.687574 -2.291780

31 1 0 0.631493 4.987590 -1.405328

32 1 0 -1.032491 3.199239 -1.894951

33 1 0 2.753342 4.397800 -0.216989

34 1 0 0.619148 -0.574424 0.401568

35 1 0 2.124412 0.153762 2.142227

36 1 0 4.334480 0.153335 0.019097

37 1 0 4.586362 0.500514 1.731525

38 1 0 -2.396971 1.741852 -0.578170

39 1 0 -3.465827 1.224598 1.521705

40 1 0 -3.673162 -1.749311 0.802731

41 1 0 -4.781684 0.711080 -0.617595

42 1 0 -2.543900 -1.183201 -1.479124

43 1 0 -2.247449 -1.255915 2.832731

44 1 0 -3.521752 -0.229882 3.529631

45 1 0 -0.984017 0.745641 2.785921

46 1 0 -5.360797 -0.937096 -2.131374

47 1 0 -2.485639 0.299225 -3.212880

48 1 0 -5.954199 -1.134073 1.025467

49 1 0 4.223643 -2.132395 -0.164516

50 1 0 3.683645 -1.871770 2.214784

51 1 0 1.986233 -2.231625 1.914993

52 1 0 4.036950 -4.064589 1.060499

53 1 0 0.448349 -2.741648 0.258175

54 1 0 0.181698 -2.662883 -1.492550

55 1 0 1.028656 -4.049849 -0.771089

56 1 0 3.086861 -3.749443 -2.266029

57 1 0 2.106594 -2.520680 -3.100033

58 1 0 3.730963 -2.110048 -2.510398

---------------------------------------------------------------------

Conformer **27i**


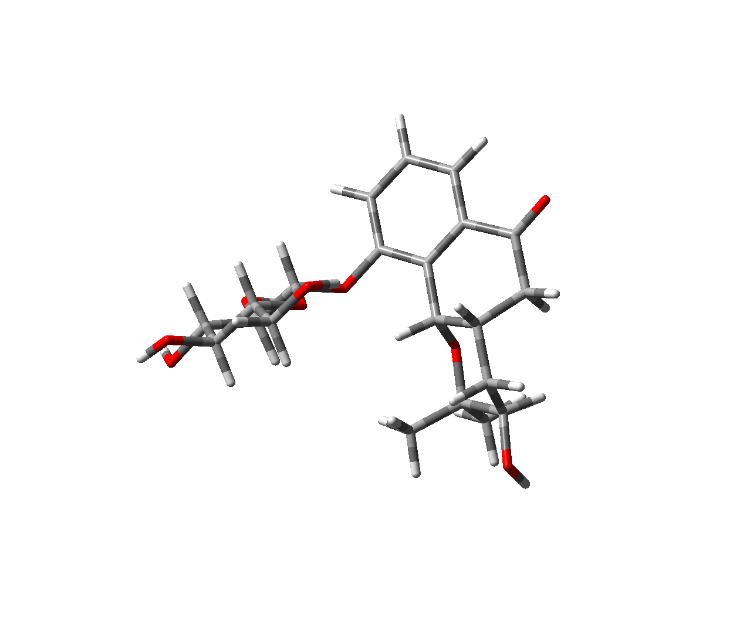


Table S10 Standard orientation of **27i**:

---------------------------------------------------------------------

Center Atomic Atomic Coordinates (Angstroms)

Number Number Type X Y Z

---------------------------------------------------------------------

1 6 0 0.824625 3.960462 -1.111765

2 6 0 -0.113395 2.960493 -1.379306

3 6 0 0.136700 1.648529 -0.976246

4 6 0 1.324761 1.298040 -0.319095

5 6 0 2.264925 2.317231 -0.067127

6 6 0 2.006371 3.640666 -0.453640

7 6 0 1.573843 -0.138188 0.094031

8 6 0 2.589694 -0.258133 1.238735

9 6 0 3.853107 0.558080 0.917182

10 6 0 3.552597 2.014513 0.628683

11 8 0 4.342258 2.904255 0.924826

12 8 0 -0.779272 0.646759 -1.276981

13 6 0 -2.024015 0.703138 -0.625572

14 8 0 -1.849870 0.214059 0.696793

15 6 0 -3.062305 0.215183 1.470847

16 6 0 -4.080594 -0.722384 0.812984

17 6 0 -4.310627 -0.296134 -0.636432

18 6 0 -2.991196 -0.196968 -1.398259

19 6 0 -2.655254 -0.204512 2.877729

20 8 0 -1.713851 0.701829 3.434804

21 8 0 -5.190332 -1.250576 -1.219154

22 8 0 -3.288224 0.329521 -2.683852

23 8 0 -5.290397 -0.665228 1.555912

24 8 0 2.050200 -0.827399 -1.075392

25 6 0 2.232102 -2.259652 -0.930052

26 6 0 3.258572 -2.497209 0.214383

27 6 0 2.882230 -1.746911 1.494080

28 8 0 3.376325 -3.878208 0.565235

29 6 0 0.895930 -2.975925 -0.684346

30 6 0 2.817411 -2.693999 -2.276398

31 1 0 0.629039 4.981048 -1.426631

32 1 0 -1.033573 3.189499 -1.908343

33 1 0 2.751502 4.398160 -0.236033

34 1 0 0.618935 -0.569833 0.410118

35 1 0 2.128540 0.163790 2.142248

36 1 0 4.333667 0.155566 0.014609

37 1 0 4.589494 0.508759 1.725187

38 1 0 -2.399990 1.736918 -0.593462

39 1 0 -3.474601 1.235243 1.505913

40 1 0 -3.671788 -1.745532 0.813176

41 1 0 -4.782854 0.699392 -0.631658

42 1 0 -2.537337 -1.195658 -1.471027

43 1 0 -2.254499 -1.229826 2.844230

44 1 0 -3.533901 -0.200716 3.527093

45 1 0 -0.995980 0.774192 2.783244

46 1 0 -5.353724 -0.962561 -2.132741

47 1 0 -2.477535 0.274407 -3.215873

48 1 0 -5.955317 -1.134489 1.023263

49 1 0 4.230190 -2.126208 -0.143939

50 1 0 3.693091 -1.860511 2.223309

51 1 0 1.995651 -2.221085 1.933559

52 1 0 3.832256 -4.333827 -0.159743

53 1 0 0.445769 -2.724383 0.280174

54 1 0 0.186093 -2.705941 -1.473174

55 1 0 1.044179 -4.058913 -0.701036

56 1 0 3.040061 -3.766629 -2.281665

57 1 0 2.104340 -2.494792 -3.082945

58 1 0 3.740695 -2.144081 -2.487887

---------------------------------------------------------------------

1. [↑](#footnote-ref-1)
